# Profile and dynamics of infectious diseases: a population-based observational study using multi-source big data

**DOI:** 10.1186/s12879-022-07313-6

**Published:** 2022-04-04

**Authors:** Lin Zhao, Hai-Tao Wang, Run-Ze Ye, Zhen-Wei Li, Wen-Jing Wang, Jia-Te Wei, Wan-Yu Du, Chao-Nan Yin, Shan-Shan Wang, Jin-Yue Liu, Xiao-Kang Ji, Yong-Chao Wang, Xiao-Ming Cui, Xue-Yuan Liu, Chun-Yu Li, Chang Qi, Li-Li Liu, Xiu-Jun Li, Fu-Zhong Xue, Wu-Chun Cao

**Affiliations:** 1 Institute of EcoHealth, School of Public Health, Cheeloo College of Medicine, Shandong University, Jinan, China; 2 Department of Occupational Health and Occupational Medicine, School of Public Health, Cheeloo College of Medicine, Shandong University, Jinan, China; 3 Institute for Medical Dataology, School of Public Health, Cheeloo College of Medicine, Shandong University, 12550 Erhuan Donglu, Jinan, 250002 China; 4 State Key Laboratory of Pathogen and Biosecurity, Beijing Institute of Microbiology and Epidemiology, 20 Dong-da Street, Fengtai District, Beijing, 100071 China

**Keywords:** Infectious disease profile, Big data, Incidence density, Epidemiological characteristics, Spatiotemporal dynamics

## Abstract

**Background:**

The current surveillance system only focuses on notifiable infectious diseases in China. The arrival of the big-data era provides us a chance to elaborate on the full spectrum of infectious diseases.

**Methods:**

In this population-based observational study, we used multiple health-related data extracted from the Shandong Multi-Center Healthcare Big Data Platform from January 2013 to June 2017 to estimate the incidence density and describe the epidemiological characteristics and dynamics of various infectious diseases in a population of 3,987,573 individuals in Shandong province, China.

**Results:**

In total, 106,289 cases of 130 infectious diseases were diagnosed among the population, with an incidence density (ID) of 694.86 per 100,000 person-years. Besides 73,801 cases of 35 notifiable infectious diseases, 32,488 cases of 95 non-notifiable infectious diseases were identified. The overall ID continuously increased from 364.81 per 100,000 person-years in 2013 to 1071.80 per 100,000 person-years in 2017 (*χ*^2^ test for trend, *P* < 0.0001). Urban areas had a significantly higher ID than rural areas, with a relative risk of 1.25 (95% CI 1.23–1.27). Adolescents aged 10–19 years had the highest ID of varicella, women aged 20–39 years had significantly higher IDs of syphilis and trichomoniasis, and people aged ≥ 60 years had significantly higher IDs of zoster and viral conjunctivitis (all *P *< 0.05).

**Conclusions:**

Infectious diseases remain a substantial public health problem, and non-notifiable diseases should not be neglected. Multi-source-based big data are beneficial to better understand the profile and dynamics of infectious diseases.

**Supplementary Information:**

The online version contains supplementary material available at 10.1186/s12879-022-07313-6.

## Introduction

Infectious diseases remain a leading cause of human morbidity and mortality in the world [1]. The epidemiological distribution and trends of infectious diseases, especially of non-notifiable infections, have rarely been investigated in mainland China [2], making it difficult to refine targeted prevention and control strategies. Currently, China is mainly relying on a traditional surveillance system, the China Information System for Disease Control and Prevention, to monitor notifiable infectious diseases [3]. Other infectious diseases are usually unreported; therefore, the burden of infectious diseases has been underestimated.

Along with the rapid development of computer science and information technology, massive datasets are available for infectious diseases, which provide a new approach to monitor all types of infectious diseases [4]. In this study, we used a dataset derived from Shandong Multi-Center Healthcare Big Data Platform (SMCHBDP) to describe the full spectrum of infectious diseases in a population of 3,987,573 individuals in Shandong province, China, and characterize the incidence density, population, and spatiotemporal distributions of all types of infectious diseases to guide evidence-based decision-making for the prevention and control of infectious diseases.

## Methods

### Integration and management of multi-source data

SMCHBDP is a hybrid system developed by the Health Commission of Shandong Province in 2017. Multiple data of health-related sources involving electronic health records, electronic medical records, the resident medical insurance payment system, and death registry were integrated through identity card numbers (Additional file 1: Fig. S1). Information regarding demographic characteristics (sex, age, and residence location) and medical history (diagnoses, symptoms, therapies, payment records, past diseases, and chronic diseases) was extracted and included into the database for analysis.

### The studied population

SMCHBDP had initially recruited potentially eligible participants who were classified into nine subgroups according to the data sources and population characteristics (Additional file 1: Table S1). The participants who did not provide necessary information or had duplicate or incorrect identity card numbers were excluded. This study was approved by the Ethics Committee of the School of Public Health, Shandong University, China. All information regarding individual persons has been anonymized.

### Data extraction

Individual records concerning infectious diseases were extracted from January 1, 2013, to June 30, 2017, and the outcome of interest was the incidence of specific infectious diseases. All infectious diseases were defined according to the Law of the People’s Republic of China on Prevention and Treatment of Infectious Diseases (2013 Amendment; Additional file 1: Text S1) and the 10th revision codes established by the International Statistical Classification of Diseases and Related Health Problems (ICD−10). Infectious diseases were divided into five categories based on the main transmission route, i.e., respiratory, gastrointestinal, vector-borne, blood- and sexually transmitted, and mucocutaneous infections [5]. Data were collected and cleaned by Shandong University. Researchers can only use the encrypted data on the SMCHBDP server after approval by the official review committee.

### Statistical analysis

The ID of infectious diseases was defined as the number of new cases (incident number) divided by person-time over the period (per 100,000 person-years). We compared the ID of each disease and disease category, stratified by sex, age, and area (urban vs. rural and prefecture-level dimensions), and estimated the relative risks (RRs) and their 95% confidence intervals (CIs) using Miettinen’s formula. The annual incidence trend was identified by the Cochran Armitage test. The monthly ID was calculated to demonstrate the changing pattern and seasonal distribution of infectious diseases. Joinpoint regression models were used to estimate the annual percentage change (APC) of each infectious disease from 2013 to 2017 [6]. Prefecture-level disease maps were visualized using ArcGIS software (version 10.3; ESRI Inc., Redlands, CA, USA). All descriptive analyses were conducted using R software (version 3.4.1, R Foundation for Statistical Computing, Vienna, Austria), and joinpoint regression analyses were conducted using the Joinpoint Regression Program (version 4.8.0.1, National Cancer Institute, MD, USA).

## Results

In total, 3,987,573 participants from 16 prefecture-level cities of Shandong province, China, were included in our study. The sample size, geographical coverage, and composition of participants in each city are displayed in Additional file 1: Fig. S1. Among them, 47.81% were male and 57.30% lived in urban areas. More than 3,000,000 participants were recruited in 2013 (Table 1).

**Table 1:** Basic information of study participants, January 2013 to June 2017

	Participants (n = 3,987,573)	*P* value*
Sex distribution of population, n (%)		
Male	1,906,485 (47.81)	
Female	2,081,088 (52.19)	
Urban–rural distribution of population, n (%)		
Urban	2,284,975 (57.30)	
Rural	1,702,598 (42.70)	
Recruiting time (year), n (%)		
2013	3,017,631 (75.67)	
2014	338,850 (8.50)	
2015	332,129 (8.33)	
2016	214,279 (5.37)	
2017	84,684 (2.12)	
Case number of infectious diseases (incidence density, per 100,000 person–years)		
Total	106,289 (694.86)	< 0.0001
Notifiable infections	73,801 (482.47)	< 0.0001
Non–notifiable infections	32,488 (212.39)	< 0.0001
Case number of five categories of infectious diseases (incidence density, per 100,000 person–years)		
Respiratory	61,951 (405.00)	< 0.0001
Gastrointestinal	9862 (64.47)	< 0.0001
Vector–borne	1128 (7.37)	< 0.0001
Blood– and sexually transmitted	10,170 (66.48)	< 0.0001
Mucocutaneous	23,178 (151.52)	< 0.0001
Annual case number of infectious diseases (incidence density, per 100,000 person–years)		
2013	10,313 (364.81)	
2014	12,813 (397.04)	
2015	27,181 (771.20)	
2016	35,167 (931.37)	
2017	20,815 (1071.80)	

*Calculated by *χ*² test for trend from 2013 to 2017

From January 2013 to June 2017, 106,289 cases belonging to 130 types of infectious diseases were identified, with an ID of 694.86 per 100,000 person-years. Approximately two-thirds (73,801) of the cases belonged to 35 notifiable infectious diseases, while up to 95 types of non-notifiable infectious diseases were discovered, involving 32,488 cases. The most common infectious diseases were respiratory, followed by mucocutaneous. The ID of vector-borne infections was the lowest (Table 1).

**Fig. 1:**
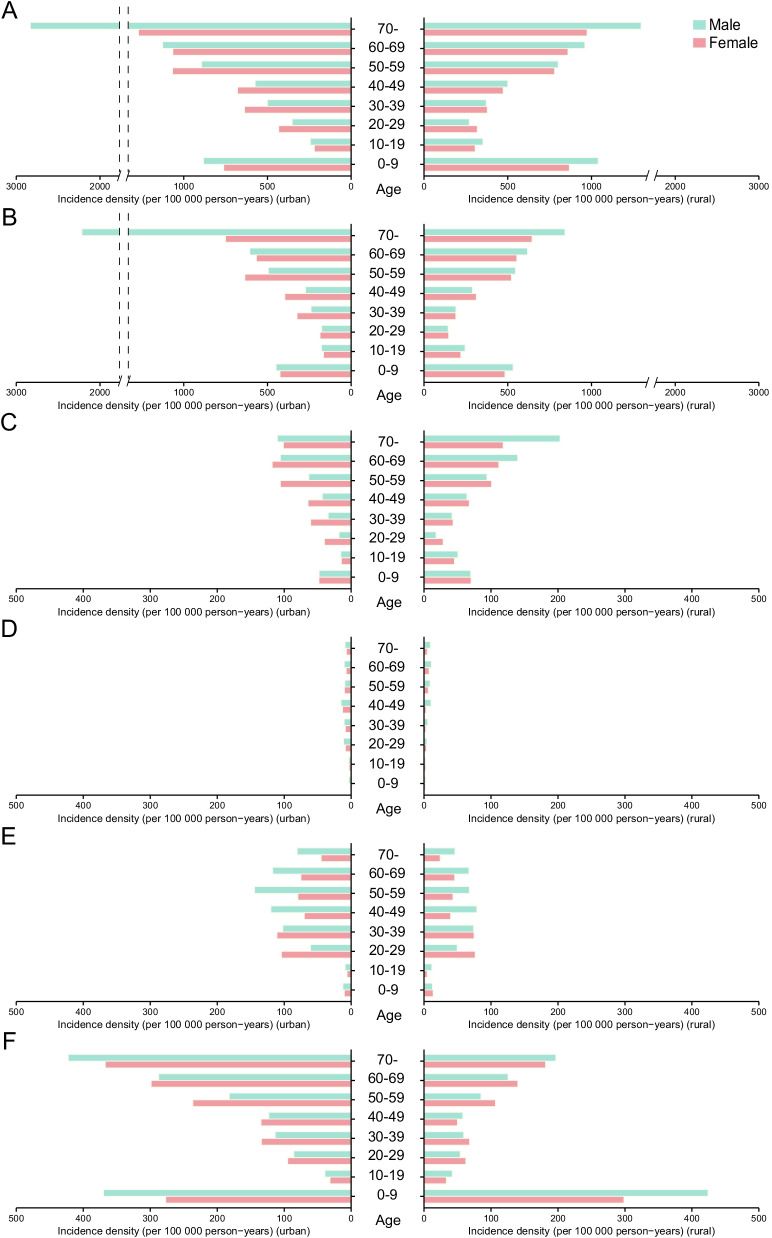
Age and sex differences related to the incidence density of infectious diseases across different geographical regions (urban vs. rural dimension). **A** Total infections. **B** Respiratory infections. **C** Gastrointestinal infections. **D** Vector-borne infections. **E** Blood- and sexually transmitted infections. **F** Mucocutaneous infections

Overall, urban areas had a significantly higher ID of infectious diseases than rural areas (RR 1.25, 95% CI 1.23–1.27). As to each category, the ID of gastrointestinal infections was significantly higher in rural areas (RR 1.19, 95% CI 1.15–1.24), while the IDs of the other four categories were significantly higher in urban areas (all *P* < 0.05; Fig. 1). For respiratory infections, people aged ≥ 70 years had an approximately 3-times higher risk compared with younger age groups (RR 3.23, 95% CI 3.17–3.29). Men had a 12% higher risk than women (RR 1.12, 95% CI 1.10–1.14). The ID of gastrointestinal infections was highest in people aged ≥ 60 years (all *P* < 0.05). Women aged 20–59 years were more vulnerable to gastrointestinal infections than men of the same age group (all *P* < 0.05). The working population aged 40–49 years had a significantly higher ID of vector-borne infections than any other age group (all *P* < 0.05). Blood- and sexually transmitted diseases were most common in young adults aged 30–39 years (RR 1.56, 95% CI 1.49–1.63). In urban areas, the ID was the highest among those aged 50–59 years for men and among those aged 20–39 years for women (all *P* < 0.05). Children aged < 10 years and older individuals aged ≥ 70 years had a significantly higher ID of mucocutaneous infections than those aged 10–69 years (RR 3.05, 95% CI 2.96–3.14 and RR 2.69, 95% CI 2.59–2.79, respectively; Fig. 1).

**Fig. 2:**
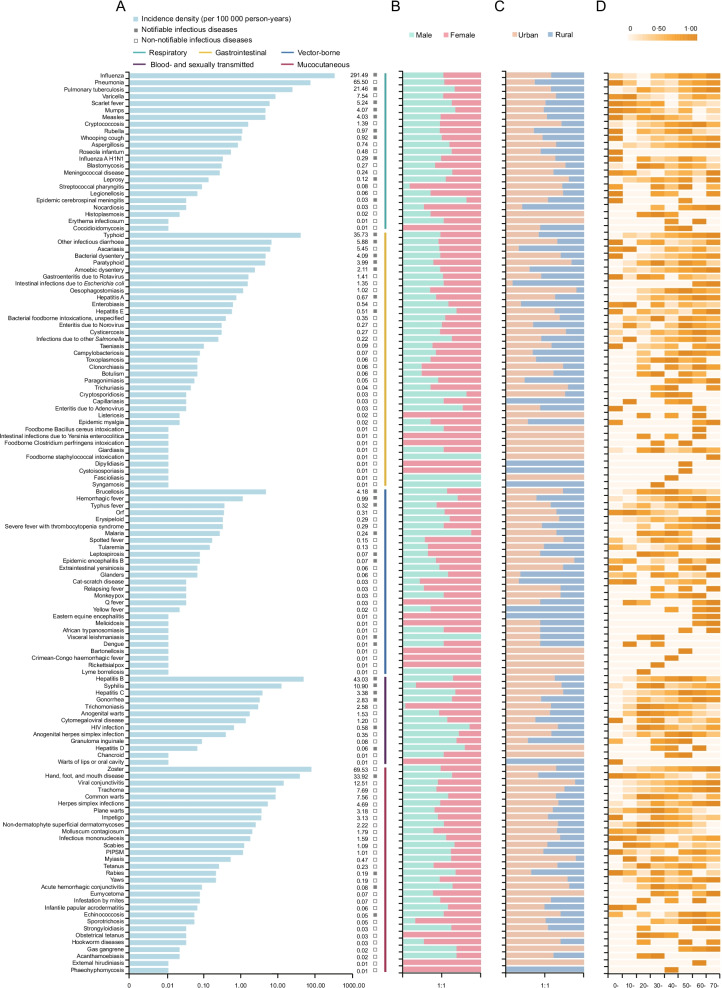
Incidence density of 130 infectious diseases and incidence density stratified by sex, urban–rural region, and age group. **A** IDs of 130 infectious diseases (per 100,000 person-years). **B** Incidence ratio between male and female individuals. **C** Incidence ratio between urban and rural populations. **D** Age-stratified IDs. The ID of each infectious disease is standardized from 0 to 1 according to the percentile rank and is represented by heat maps (with color scale from 0 to 1, where 1 is the highest incidence and 0 is the lowest incidence). *ID* incidence density, *HIV* human immunodeficiency virus, *PIPSM * picornavirus infections presenting in the skin or mucous membranes

Among the 130 types of infectious diseases, 23 were respiratory, 37 gastrointestinal, 27 vector-borne, 13 blood- and sexually transmitted, and 30 mucocutaneous. The most frequent infections were influenza, followed by zoster and pneumonia, which accounted for 61.38% of all cases. Additionally, typhoid was the most common gastrointestinal infection, hepatitis B was the most common blood- and sexually transmitted disease, and brucellosis was the most common vector-borne infection (Fig. 2, Additional file 1: Table S1).

The most vulnerable groups were identified for each infection type (Fig. 2, Additional file 1: Fig. S2). Young people aged < 20 years in rural areas were more likely to be infected with mumps and scarlet fever (all *P* < 0.05). Infants and children aged 0–10 years had the highest ID of pneumonia, whereas adolescents aged 10–19 years had the highest ID of varicella (all *P* < 0.05). Some gastrointestinal infections, such as gastroenteritis due to Rotavirus and other infectious diarrhea, had the highest IDs in children aged < 10 years, while amoebic dysentery had the highest ID in older individuals aged ≥ 60 years (all *P* < 0.05). Women aged 20–39 years had significantly higher IDs of syphilis and trichomoniasis (all *P* < 0.05). Most new cases of human immunodeficiency virus (HIV) infection occurred in young people aged 20–29 years (RR 2.74, 95% CI 1.80–4.16), and men had a 60-fold higher risk than women in this age group (RR 60.12, 95% CI 34.20–105.69). The working population aged 40–49 years had the highest ID of brucellosis (RR 2.04, 95% CI 1.73–2.41). Among mucocutaneous infections, people aged ≥ 60 years had the highest IDs of zoster and viral conjunctivitis, while infants and children aged < 10 years had the highest ID of “hand, foot, and mouth disease” (all *P* < 0.05).

**Fig. 3:**
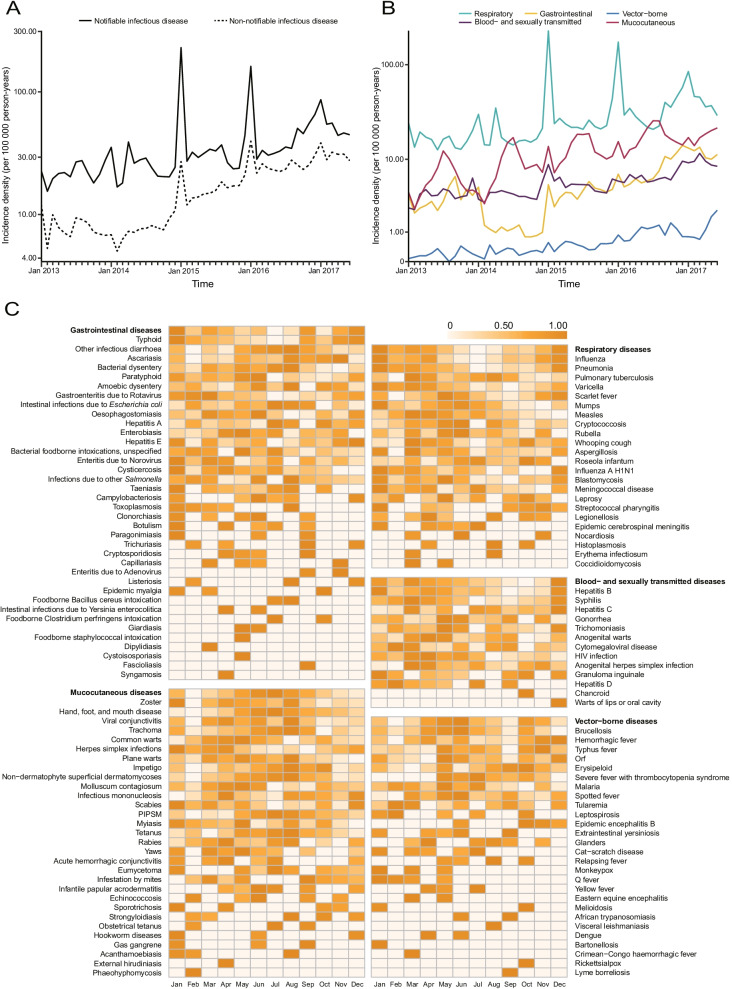
Trends in incidence density of infectious diseases by reporting type and transmission route and seasonality of infectious diseases. **A** Monthly IDs of infectious diseases by reporting type, from January 2013 to June 2017. **B** Monthly IDs of infectious diseases by transmission route, from January 2013 to June 2017. **C** Seasonal distribution of infectious diseases. *ID* incidence density, *HIV* human immunodeficiency virus, *PIPSM* picornavirus infections presenting in the skin or mucous membranes

Figure 3 illustrates the secular trends and seasonality of infectious diseases from 2013 to 2017. The ID of all infectious diseases increased remarkably from 364.81 to 1,00,000 person-years in 2013 to 1071.80 per 1,00,000 person-years in 2017, and a similar trend was observed for the five categories of infections (*χ*^2^ test for trend, all *P* < 0.0001; Table 1; Fig. 3 A and 3B). For both notifiable and non-notifiable diseases, the overall ID increased continuously throughout the study period, and the rise in non-notifiable diseases was greater than that in notifiable diseases, with an annual increase of 32.84% and 20.30%, respectively (Fig. 3A). Significant seasonal features were noted in different categories of infectious diseases. Respiratory infections peaked in winter (mainly in January), whereas gastrointestinal infections peaked twice per year (January and August to September). Moreover, the highest ID of vector-borne diseases and mucocutaneous infections typically occurred in summer (between May and August). In contrast, there was less seasonality in the pattern of blood- and sexually transmitted diseases (Fig. 3C). The changing pattern and seasonal distribution of 130 infectious diseases are shown in the appendix (Additional file 1: Figs. S3–S4). We further analyzed the seasonality of the five categories of infectious diseases in 16 cities, and a similar pattern was observed (Additional file 1: Fig. S5).

**Fig. 4:**
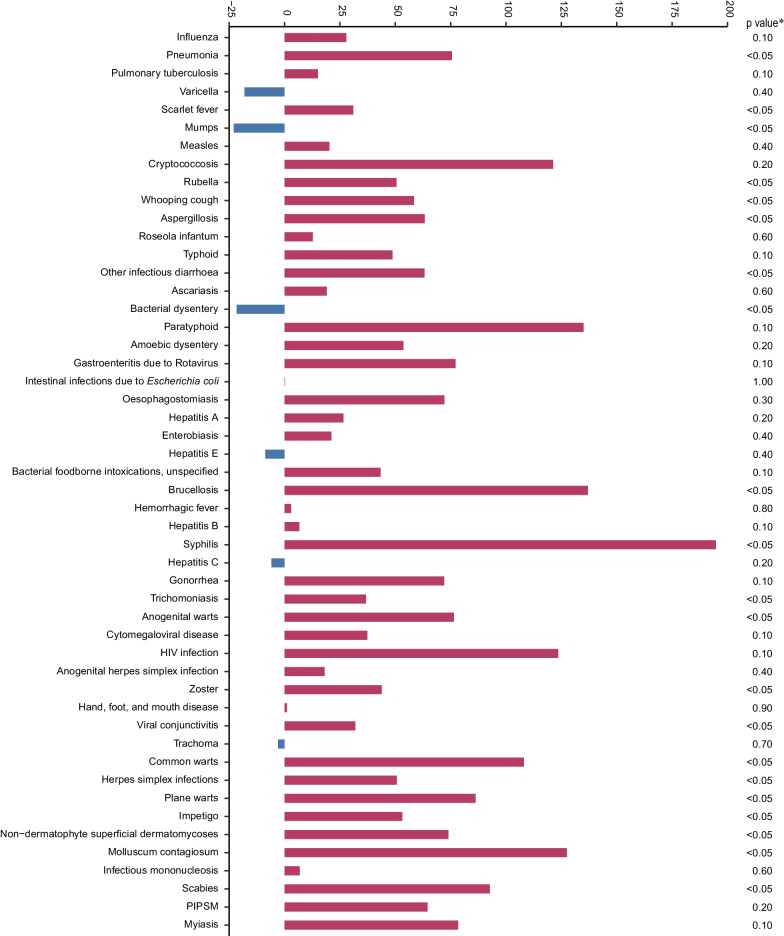
Annual percentage change in the incidence density of the top 50 infectious diseases. The annual percentage change (APC) of each infectious disease is estimated using the joinpoint regression models. The Z-test is used to assess whether an APC is significantly different from zero. If the APC is significant (*P *< 0.05), the incidence trend is identified as an increase or decrease; otherwise, the incidence is maintained stable. ^*^Joinpoint regression provided significant values (*P* < 0.05). *HIV* human immunodeficiency virus, *PIPSM* picornavirus infections presenting in the skin or mucous membranes

According to the joinpoint regression analysis, 19 of the top 50 infectious diseases showed a significantly increasing trend in ID from 2013 to 2017, including six notifiable infectious diseases and 13 non-notifiable infectious diseases (all *P* < 0.05). Conversely, the IDs of mumps and bacterial dysentery decreased significantly during the study period (all *P* < 0.05), with annual declines of 29.0% and 21.6%, respectively (Fig. 4). In addition, the IDs of some imported and parasitic diseases notably increased as evidenced by the higher APCs (Additional file 1: Fig. S6).

**Fig. 5:**
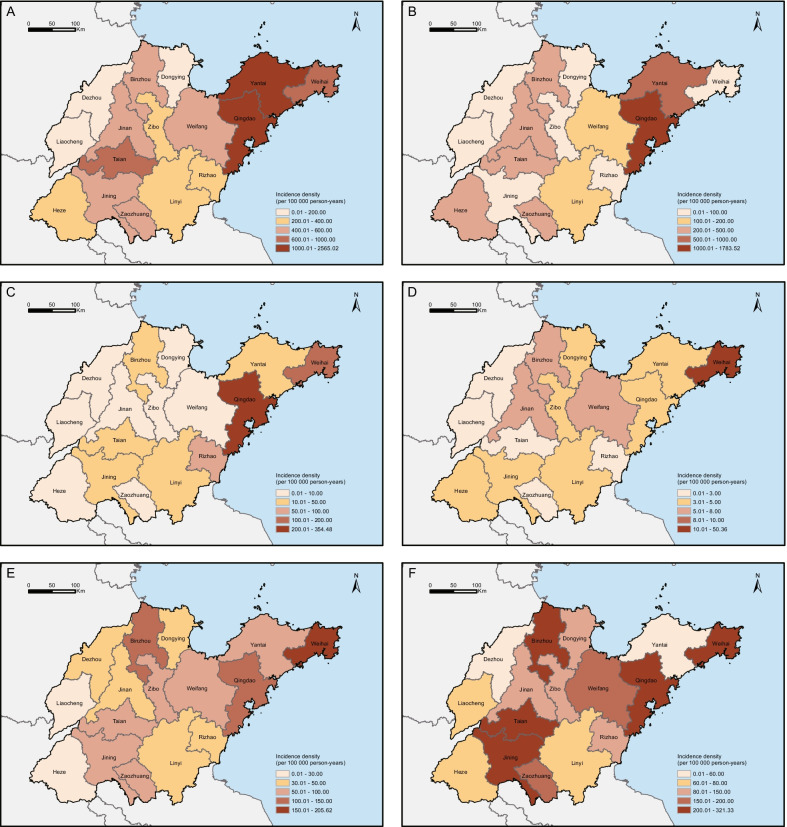
Geographical distribution of infectious diseases in Shandong province, China. Thematic maps represent the prefecture-level incidence densities of infectious diseases. **A** Total infections. **B** Respiratory infections. **C** Gastrointestinal infections. **D** Vector-borne infections. **E** Blood- and sexually transmitted infections. **F** Mucocutaneous infections

The IDs of infectious diseases varied greatly across the geographical regions. Overall, Jiaodong Peninsula had a higher ID than did inland areas (RR 4.66, 95% CI 4.61–4.71, Fig. 5A). Figure panels 5B–5 F show the incidence maps of the five categories of infectious diseases. Among the 16 prefecture-level cities, Qingdao had the highest IDs of respiratory, gastrointestinal, and mucocutaneous infections (all *P *< 0.05). The IDs of vector-borne and blood- and sexually transmitted infections were highest in Weihai (all *P* < 0.05). In addition, notifiable infectious diseases were mainly concentrated in Jiaodong Peninsula, whereas the non-notifiable infectious diseases were distributed in a more scattered manner (Additional file 1: Fig. S7).

## Discussion

This population-based observational study identified 130 infectious diseases among nearly four million individuals in Shandong province, China, with an ID ranging from 0.01 to 291.49 per 100,000 person-years. A significant increasing trend in ID was noted from January 2013 to June 2017. To our knowledge, this was the first study to utilize a multi-source big data platform to actively monitor all types of infectious diseases in China. The dataset enabled us to profile the epidemic features of notifiable infectious diseases and to report the status of non-notifiable infections.

During the study period, 73,801 cases of 35 notifiable infectious diseases and 32,488 cases of 95 non-notifiable infectious diseases were identified. These non-notifiable infections might also cause substantial morbidity, and their public health significance deserves further investigation. The three infectious diseases with the highest ID were influenza, zoster, and pneumonia, of which the latter two are non-notifiable infectious diseases. Globally, influenza and pneumonia lead to considerable morbidity and mortality with high healthcare costs [7, 8], while zoster is a major health burden that can affect individuals of any age [9].

We noted that infectious diseases were more common in urban than in rural areas. During the last few decades, China has witnessed an unprecedented economic boom and rapid urbanization. In urban areas, high population density, increased shared airspace, and heavy air pollution have increased the risk of respiratory infections [10]. Moreover, urbanization and globalization have increased the probability of zoonotic emergence in urban areas by affecting the ecology and distribution of arthropod vectors and increasing the spread of new infections globally [11]. Economic growth has also led to the reemergence of sex services; the influx of many rural-to-urban male migrant workers without spouses has created a growing demand for sex services, increasing the transmission of blood- and sexually transmitted diseases [12].

In contrast, gastrointestinal infections were more frequent in rural than in urban areas. This may be attributable to lower socioeconomic statuses, poor sanitation, and malnutrition in rural areas [13]. In addition, some vector-borne diseases, especially natural-focal diseases such as Q fever and hemorrhagic fever, were more common in rural than in urban areas, likely because rural areas tend to have diverse habitats that can be exploited by more species of vectors [14].

Sex and age groups that are vulnerable to infectious diseases were identified in the present study. Some respiratory infections tended to mostly affect men aged ≥ 70 years. This may be due to the substantial adverse effects of smoking on the immune system and respiratory tract [15]. With the introduction of global varicella vaccination programs, the burden of varicella has been reduced. However, most countries (including China) have chosen to vaccinate high-risk groups rather than to conduct universal vaccination [16]. In our study, adolescents between ages 10 and 19 who were not prioritized for varicella vaccination [17], had the highest ID of varicella. Zoster is a mucocutaneous infection caused by the reactivation of the latent varicella zoster virus (VZV). The recombinant zoster vaccine, which is the most effective measure to prevent zoster infection, has been introduced into China in 2019. Besides, ageing of the population, reduced immune function, and previous VZV exposure are also important reasons for the high incidence of zoster among older individuals [18, 19]. Therefore, continuous surveillance of VZV infections is necessary to assess the need for catch-up vaccination in high-risk groups.

We found that, among individuals aged 20–39 years, the risk of contracting gastrointestinal infections was higher in women than in men; the reasons for this sex difference are unclear. Besides host factors, women as the principal food handlers in China may have a higher risk of exposure to foodborne gastrointestinal infections [20]. Regarding mucocutaneous diseases, viral conjunctivitis had the highest IDs in adults aged ≥ 60 years, partly because of the gradual decline of the immune system with age, whereas “hand, foot, and mouth disease” dominated in children aged < 10 years. Such variations suggest the need for age-specific control measures for different mucocutaneous diseases. Based on the findings of our study, adults aged 40–49 years, especially men, are most affected by vector-borne infections, possibly because this age group most actively herds or handles domestic animals; thus, they have a higher risk of exposure to vector-borne infections [21].

In our study, the ID of blood- and sexually transmitted diseases was higher in men than in women. One possible reason is that sexually transmitted infections in men who have sex with men have increased since the late 1990 [22]. Additionally, the relaxation of traditional cultural norms has yielded new patterns of behavior that involve changes in sexual activity and the use of recreational drugs [10]. Notably, men living in urban areas aged 50–59 years had the highest ID of blood- and sexually transmitted infections. Many adults continue to be sexually active throughout their lives. Among them, men living in urban areas nearing retirement are more likely to engage in unsafe sex [23]. Our study indicated that women of child-bearing age (20–39 years) are emerging as another vulnerable group of blood- and sexually transmitted infections, as their open-minded attitude toward sex and high-risk sexual behavior have increased the risk of spreading blood- and sexually transmitted diseases [24].

The overall ID of infectious diseases showed a fluctuating upward trend during the study period. This could be due to increasing antimicrobial resistance, changes in human behavior, and improved screening intensity and diagnostic technologies. Notably, the IDs of some infections have alarmingly increased, mainly involving imported infectious and parasitic diseases. In our study, imported infectious diseases were reported nearly every year. These imported cases are related to population influx from other countries because of globalization [12]. Thus, pretravel education for migrant workers and tourists in China should be strengthened, and border screening and early warnings should be further improved [25]. Large-scale and aggressive case screening strategies for specific parasites in China may lead to a substantial increase in detected cases of parasitic diseases [26]. Therefore, enhanced surveillance and investigations are required to control these infections.

Our results demonstrated that the epidemic peak of respiratory infections occurred in winter, whereas the peaks of vector-borne diseases and mucocutaneous infections occurred in summer. Weather conditions in winter could affect not only the viability and transmission of respiratory viruses, but also host immunity, increasing the contact between infected and susceptible populations [27]. This facilitates the spread of respiratory infectious diseases, such as influenza and pneumonia [28]. Seasonality is a well-known epidemiological phenomenon in vector-borne diseases, partly because vectors are greatly influenced by meteorological factors [29]. Additionally, two epidemic peaks were observed for gastrointestinal infections (i.e., summer and winter), which might be due to changes in the pathogen spectrum over time.

In the present study, Jiaodong Peninsula was identified as the area where individuals had the highest risk of contracting infectious diseases. The possible reasons are as follows. The Jiaodong Peninsula region is more developed and more densely populated than any other region in Shandong province, China. These cities have attracted large mobile populations, characterized by weak immune systems, poor living conditions, and poor knowledge on epidemic prevention [30], and populations in these regions are susceptible to various infectious diseases. Among all cities, Qingdao is a hotspot for many infectious diseases. As an important hub for international trade and transportation, frequent international population movement in Qingdao could favor the emergence and spread of infectious diseases [5]. Additionally, most big data systems differing in reporting and health-seeking behaviors across regions are pervasive [31], and further investigations are required to confirm our geographical variations.

Our study has some limitations. First, variations in diagnostic procedures, experimental conditions, technical levels, and reporting times of different institutions and periods could have introduced bias into the reported IDs. Second, the case number of typhoid may be overestimated because its definition in medical records may be confused with the definition of exogenous febrile diseases in Chinese medicine. Third, the study population was not stratified for comorbidities and immunological condition because of unavailability of data and information.

## Conclusions

In conclusion, we identified 130 infectious diseases in approximately 4 million individuals in Shandong province, China. The challenges posed by infectious diseases, especially non-notifiable infections, have been increasing continuously. Targeted prevention strategies with an emphasis on children, older individuals, and people living in urban areas should be implemented.

## Supplementary Information

**Additional file 1.** Additional figures and tables

## Data Availability

The datasets used and/or analyzed during the current study are available from the corresponding author on reasonable request.

## References

[1] Wang L, Wang Y, Jin S (2008). Emergence and control of infectious diseases in China. Lancet.

[2] Yang S, Wu J, Ding C (2017). Epidemiological features of and changes in incidence of infectious diseases in China in the first decade after the SARS outbreak: an observational trend study. Lancet Infect Dis.

[3] Dong Y, Wang L, Burgner DP (2020). Infectious diseases in children and adolescents in China: analysis of national surveillance data from 2008 to 2017. BMJ.

[4] Salathé M (2016). Digital pharmacovigilance and disease surveillance: combining traditional and big-data systems for better public health. J Infect Dis.

[5] Fang LQ, Sun Y, Zhao GP (2018). Travel-related infections in mainland China, 2014–16: an active surveillance study. Lancet Public Health.

[6] Wang L, Ning P, Yin P (2019). Road traffic mortality in China: analysis of national surveillance data from 2006 to 2016. Lancet Public Health.

[7] Prina E, Ranzani OT, Torres A (2015). Community-acquired pneumonia. Lancet.

[8] Li L, Liu Y, Wu P (2019). Influenza-associated excess respiratory mortality in China, 2010–15: a population-based study. Lancet Public health.

[9] Koshy E, Mengting L, Kumar H, Jianbo W (2018). Epidemiology, treatment and prevention of herpes zoster: a comprehensive review. Indian J Dermatol Venereol Leprol.

[10] Alirol E, Getaz L, Stoll B, Chappuis F, Loutan L (2011). Urbanisation and infectious diseases in a globalised world. Lancet Infect Dis.

[11] Wilke ABB, Beier JC, Benelli G (2019). Complexity of the relationship between global warming and urbanization - an obscure future for predicting increases in vector-borne infectious diseases. Curr Opin Insect Sci.

[12] Liu Q, Xu W, Lu S (2018). Landscape of emerging and re-emerging infectious diseases in China: impact of ecology, climate, and behavior. Front Med.

[13] Shah M, Odoyo E, Wandera E (2017). Burden of rotavirus and enteric bacterial pathogens among children under five years old hospitalized with diarrhea in suburban and rural areas in Kenya. Jpn J Infect Dis.

[14] Lines J, Harpham T, Leake C, Schofield C (1994). Trends, priorities and policy directions in the control of vector-borne diseases in urban environments. Health Policy Plan.

[15] Huttunen R, Heikkinen T, Syrjänen J (2011). Smoking and the outcome of infection. J Intern Med.

[16] Papaloukas O, Giannouli G, Papaevangelou V (2014). Successes and challenges in varicella vaccine. Ther Adv Vaccines.

[17] Wong CA, Taylor JA, Wright JA, Opel DJ, Katzenellenbogen RA (2013). Missed opportunities for adolescent vaccination, 2006–2011. J Adolescent Health.

[18] Deng HJ, Liu FX (2021). Epidemiological characteristics of herpes zoster and research progress of vaccine immunization program in China. China Contin Med Educ.

[19] Du WY, Yin CN, Wang HT (2021). Infectious diseases among elderly persons: Results from a population-based observational study in Shandong province, China, 2013–2017. J Glob Health.

[20] Byrne L, Jenkins C, Launders N (2015). The epidemiology, microbiology and clinical impact of Shiga toxin-producing Escherichia coli in England, 2009–2012. Epidemiol Infect.

[21] Lwande OW, Irura Z, Tigoi C (2012). Seroprevalence of Crimean Congo Hemorrhagic Fever Virus in Ijara District, Kenya. Vector-Borne Zoonotic Dis.

[22] Unemo M, Bradshaw CS, Hocking JS (2017). Sexually transmitted infections: challenges ahead. Lancet Infect Dis.

[23] Wang Y, Lu R, Wu G (2020). Changing Trends of HIV, Syphilis, and Hepatitis C among Male Migrant Workers in Chongqing, China: Nine Consecutive Cross-Sectional Surveys, 2010–2018. Int J Environ Res Public Health.

[24] Fu G, Shi Y, Yan Y (2018). The prevalence of and factors associated with willingness to utilize HTC service among college students in China. Bmc Public Health.

[25] Wang Y, Wang X, Liu X (2018). Epidemiology of Imported Infectious Diseases, China, 2005–2016. Emerg Infect Dis.

[26] Li Z, Gao GF (2017). Infectious disease trends in China since the SARS outbreak. Lancet Infect Dis.

[27] Moriyama M, Hugentobler WJ, Iwasaki A (2020). Seasonality of Respiratory Viral Infections. Annu Rev Virol.

[28] Davis RE, Rossier CE, Enfield KB (2012). The impact of weather on influenza and pneumonia mortality in New York City, 1975–2002: a retrospective study. PLoS ONE.

[29] Stratton MD, Ehrlich HY, Mor SM, Naumova EN (2017). A comparative analysis of three vector-borne diseases across Australia using seasonal and meteorological models. Sci Rep.

[30] Mao Y, Zhang N, Zhu B, Liu J, He R (2019). A descriptive analysis of the Spatio-temporal distribution of intestinal infectious diseases in China. BMC Infect Dis.

[31] Simonsen L, Gog JR, Olson D, Viboud C (2016). Infectious Disease Surveillance in the Big Data Era: Towards Faster and Locally Relevant Systems. J Infect Dis.

