# Supplementary material for: Profile and dynamics of infectious diseases: a population-based observational study using multi-source big data
Source: BMC Infect Dis. 2022 Apr 4;22:332. doi: 10.1186/s12879-022-07313-6 (PMC8977827; doi:10.1186/s12879-022-07313-6)
Supplement: Supplementary file 1 — Additional file 1. Additional figures and tables [file 12879_2022_7313_MOESM1_ESM.pdf]

Additional file 1

# Contents

Figure S1: Geographical distribution of participants in Shandong province, China……………………………..…………………………………..………1

Text S1: Description and classification of 39 notifiable infectious diseases in China..2

Table S1: Characteristics of infectious diseases in Shandong province, China, from January 2013 to June 2017.............................................................................................3

Figure S2: Age and sex differences related to the incidence density of the top 50 infectious diseases across different geographical regions (urban vs. rural dimension)……………………………………………………………………….9

Figure S3: Monthly incidence density of 130 infectious diseases, from January 2013 to June 2017 ..................................................................................................18

Figure S4: Seasonal distribution of 130 infectious diseases in Shandong province, China ................................................................................................................29

Figure S5: Seasonal distribution of the five categories of infectious diseases in 16 prefecture-level cities of Shandong province, China ………………………………. 51

Figure S6: Annual percentage change in the incidence density of 130 infectious diseases…………………………………………………………………………....52

Figure S7: Geographical distribution of 35 notifiable infectious diseases and 95 non-notifiable infectious diseases in Shandong province, China......................................53

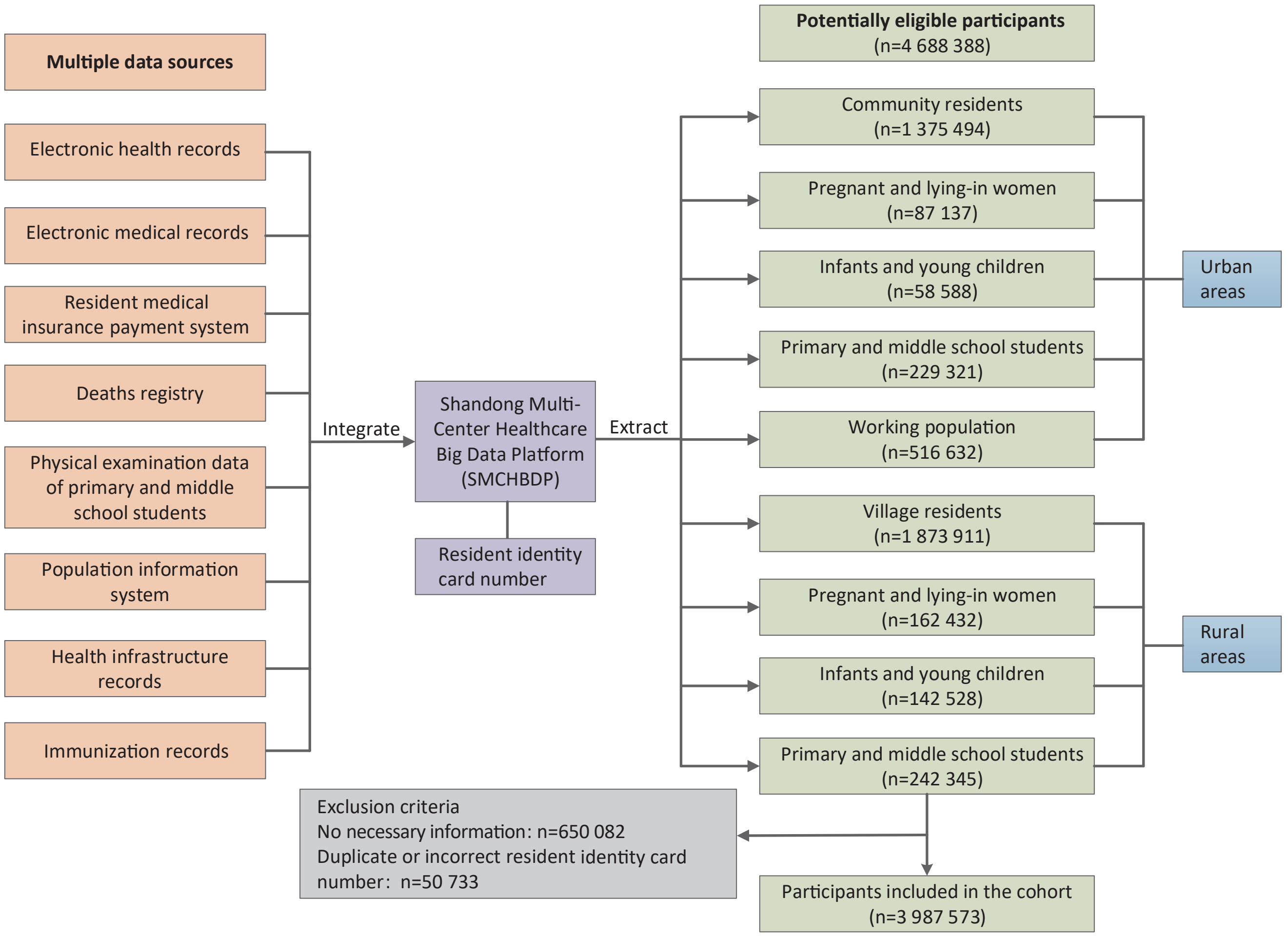


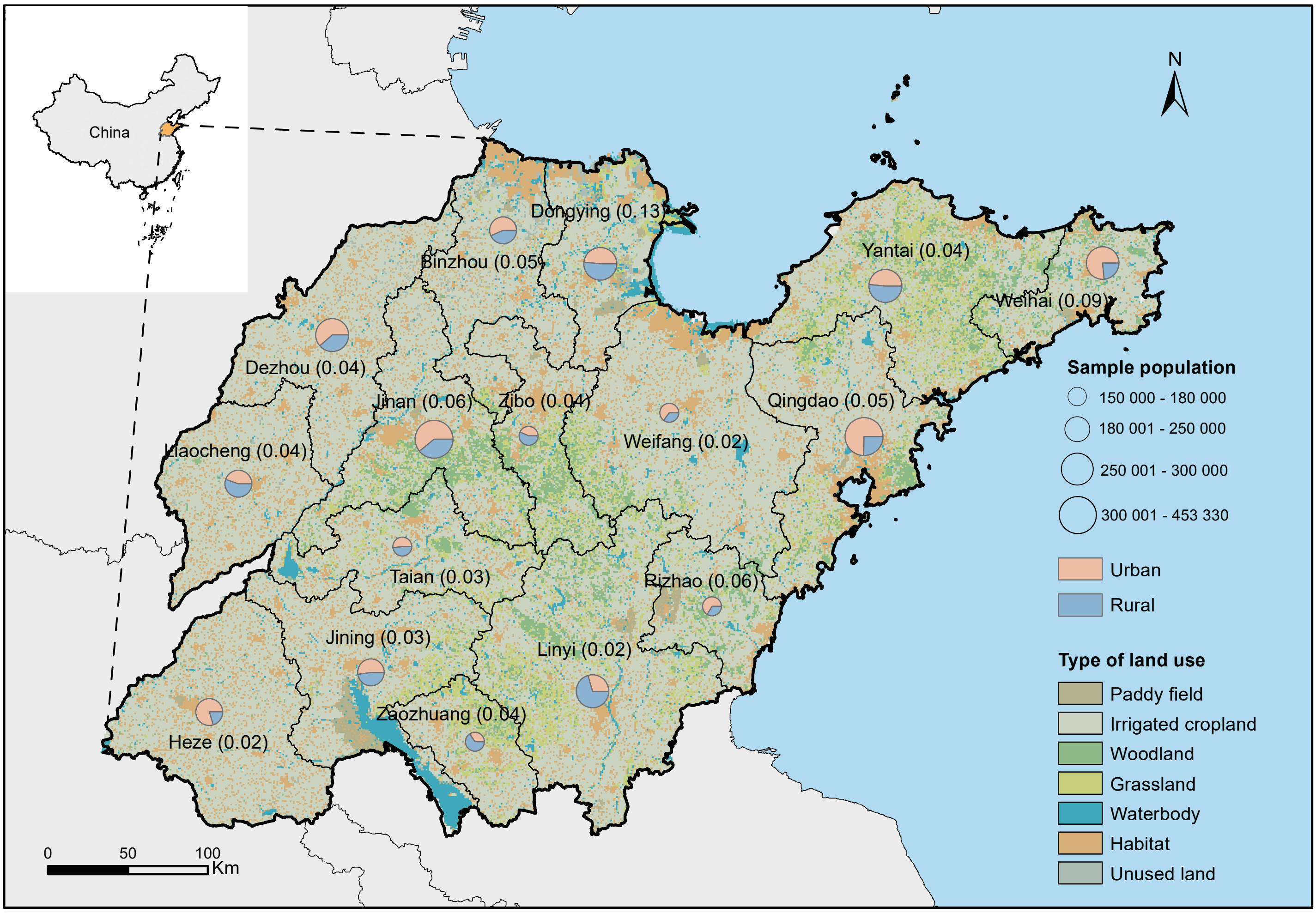


**Figure S1: Geographical distribution of participants in Shandong province, China.** Figures in brackets represent the sample proportion of each city (sample population divided by total population).

**Text S1: Description and classification of 39 notifiable infectious diseases in China**

The 39 notifiable infectious diseases are divided into the following three classes: class A, which includes plague and cholera; class B, which includes SARS, HIV infection, viral hepatitis, poliomyelitis, avian influenza H5N1, measles, haemorrhagic fever, rabies, epidemic encephalitis B, dengue, anthrax, bacillary and amoebic dysentery, pulmonary tuberculosis, typhoid and paratyphoid, epidemic cerebrospinal meningitis, whooping cough, diphtheria, tetanus neonatorum, scarlet fever, brucellosis, gonorrhoea, syphilis, leptospirosis, schistosomiasis, malaria, avian influenza H7N9; class C, which includes seasonal influenza, mumps, rubella, acute haemorrhagic conjunctivitis, leprosy, typhus fever, visceral leishmaniasis, echinococcosis, filariasis, hand, foot, and mouth disease, Other infectious diarrhoea (infectious diarrhoeal diseases other than cholera, bacterial and amoebic dysentery, typhoid and paratyphoid).

In particular, we divided viral hepatitis into hepatitis A, B, C, D, and E; bacterial and amoebic dysentery into two separate categories of bacterial dysentery and amoebic dysentery; typhoid and paratyphoid into two separate groups of typhoid and paratyphoid; and seasonal influenza into traditional seasonal influenza and influenza A H1N1.

**Table S1**

Characteristics of infectious diseases in Shandong province, China, from January 2013 to June 2017

| Infectious diseases | Case number (n,% )* | | | | | Age of onset | Incidence density (per 100 000 person-years) | | | | | Incidence density between |
|---|---|---|---|---|---|---|---|---|---|---|---|---|
| | Total | Male | Female | Urban | Rural | Median (IQR) | 2013 | 2014 | 2015 | 2016 | 2017 | 2013-2017 (per 100 000 person-years) |
| **Respiratory** | 61951 | 31443 (50.75) | 30508 (49.25) | 37361 (60.31) | 24590 (39.69) | 51 (31-64) | 201.00 | 227.54 | 495.83 | 538.3 | 572.77 | 405.00 |
| Influenza | 44588 | 22093 (49.55) | 22495 (50.45) | 28466 (63.84) | 16122 (36.16) | 54 (40-66) | 139.76 | 174.83 | 388.05 | 371.43 | 375.51 | 291.49 |
| Pneumonia | 10020 | 5081 (50.71) | 4939 (49.29) | 4300 (42.91) | 5720 (57.09) | 31 (5-61) | 15.49 | 20.23 | 59.81 | 109.24 | 138.82 | 65.50 |
| Pulmonary tuberculosis | 3282 | 2110 (64.29) | 1172 (35.71) | 2087 (63.59) | 1195 (36.41) | 49 (29-64) | 16.34 | 15.28 | 24.86 | 25.55 | 25.09 | 21.46 |
| Varicella | 1153 | 568 (49.26) | 585 (50.74) | 804 (69.73) | 349 (30.27) | 27 (12-54) | 17.05 | 2.29 | 5.14 | 5.99 | 9.79 | 7.54 |
| Scarlet fever | 801 | 484 (60.42) | 317 (39.58) | 455 (56.8) | 346 (43.20) | 6 (5-7) | 1.84 | 4.28 | 5.79 | 6.94 | 7.47 | 5.24 |
| Mumps | 623 | 405 (65.01) | 218 (34.99) | 341 (54.74) | 282 (45.26) | 10 (7-20) | 6.97 | 4.18 | 3.77 | 2.81 | 2.68 | 4.07 |
| Measles | 616 | 283 (45.94) | 333 (54.06) | 362 (58.77) | 254 (41.23) | 30 (13-42) | 2.09 | 3.90 | 4.65 | 6.38 | 1.34 | 4.03 |
| Cryptococcosis | 213 | 96 (45.07) | 117 (54.93) | 162 (76.06) | 51 (23.94) | 51 (41-61) | 0.04 | 0.15 | 0.28 | 3.52 | 3.30 | 1.39 |
| Rubella | 149 | 67 (44.97) | 82 (55.03) | 62 (41.61) | 87 (58.39) | 26 (0-45) | 0.35 | 0.56 | 0.88 | 1.48 | 1.75 | 0.97 |
| Whooping cough | 141 | 67 (47.52) | 74 (52.48) | 75 (53.19) | 66 (46.81) | 47 (4-66) | 0.21 | 0.74 | 0.57 | 1.30 | 2.16 | 0.92 |
| Aspergillosis | 113 | 65 (57.52) | 48 (42.48) | 79 (69.91) | 34 (30.09) | 65 (57-74) | 0.14 | 0.50 | 0.57 | 1.09 | 1.65 | 0.74 |
| Roseola infantum | 73 | 44 (60.27) | 29 (39.73) | 37 (50.68) | 36 (49.32) | 0 (0-0) | 0.35 | 0.28 | 0.71 | 0.64 | 0.26 | 0.48 |
| Influenza A H1N1 | 45 | 21 (46.67) | 24 (53.33) | 31 (68.89) | 14 (31.11) | 39 (24-55) | 0.07 | 0.06 | 0.23 | 0.58 | 0.57 | 0.29 |
| Blastomycosis | 41 | 16 (39.02) | 25 (60.98) | 33 (80.49) | 8 (19.51) | 37 (28-54) | 0.11 | 0.06 | 0.09 | 0.32 | 1.08 | 0.27 |
| Meningococcal disease | 36 | 22 (61.11) | 14 (38.89) | 24 (66.67) | 12 (33.33) | 48 (12-71) | 0.11 | 0.03 | 0.14 | 0.40 | 0.62 | 0.24 |
| Leprosy | 19 | 10 (52.63) | 9 (47.37) | 16 (84.21) | 3 (15.79) | 49 (32-62) | 0.04 | 0 | 0.03 | 0.32 | 0.26 | 0.12 |
| Streptococcal pharyngitis | 13 | 1 (7.69) | 12 (92.31) | 10 (76.92) | 3 (23.08) | 44 (35-54) | 0.04 | 0.06 | 0.09 | 0.13 | 0.10 | 0.08 |
| Legionellosis | 9 | 3 (33.33) | 6 (66.67) | 7 (77.78) | 2 (22.22) | 5 (3-38) | 0 | 0 | 0.03 | 0.13 | 0.15 | 0.06 |
| Epidemic cerebrospinal meningitis | 5 | 4 (80.00) | 1 (20.00) | 3 (60.00) | 2 (40.00) | 8 (6-9) | 0 | 0.03 | 0.06 | 0.03 | 0.05 | 0.03 |

| | | | | | | | | | | | | |
|---|---|---|---|---|---|---|---|---|---|---|---|---|
| Nocardiosis | 4 | 1 (25.00) | 3 (75.00) | 1 (25.00) | 3 (75.00) | 60 (50-68) | 0 | 0.03 | 0.06 | 0 | 0.05 | 0.03 |
| Histoplasmosis | 3 | 1 (33.33) | 2 (66.67) | 3 (100.00) | 0 (0.00) | 34 (31-39) | 0 | 0.03 | 0 | 0.05 | 0 | 0.02 |
| Erythema infectiosum | 2 | 1 (50.00) | 1 (50.00) | 2 (100.00) | 0 (0.00) | 53 (49-56) | 0 | 0 | 0.03 | 0 | 0.05 | 0.01 |
| Coccidioidomycosis | 2 | 0 (0.00) | 2 (100.00) | 1 (50.00) | 1 (50.00) | 51 (47-54) | 0 | 0 | 0.03 | 0 | 0.05 | 0.01 |
| **Gastrointestinal** | 9862 | 4437 (44.99) | 5425 (55.01) | 5135 (52.07) | 4727 (47.93) | 50 (33-63) | 42.48 | 16.24 | 49.96 | 94.62 | 144.33 | 64.47 |
| Typhoid | 5466 | 2500 (45.74) | 2966 (54.26) | 2630 (48.12) | 2836 (51.88) | 57 (45-66) | 27.59 | 6.20 | 22.36 | 48.78 | 95.56 | 35.73 |
| Other infectious diarrhoea† | 899 | 389 (43.27) | 510 (56.73) | 641 (71.3) | 258 (28.7) | 31 (3-53) | 1.49 | 3.32 | 4.34 | 10.14 | 11.02 | 5.88 |
| Ascariasis | 833 | 370 (44.42) | 463 (55.58) | 166 (19.93) | 667 (80.07) | 33 (10-50) | 2.79 | 0.43 | 9.67 | 8.05 | 4.89 | 5.45 |
| Bacterial dysentery | 625 | 285 (45.60) | 340 (54.40) | 469 (75.04) | 156 (24.96) | 46 (23-60) | 7.25 | 3.78 | 3.57 | 3.02 | 2.99 | 4.09 |
| Paratyphoid | 610 | 225 (36.89) | 385 (63.11) | 531 (87.05) | 79 (12.95) | 41 (32-54) | 0.21 | 0.12 | 0.31 | 9.11 | 12.61 | 3.99 |
| Amoebic dysentery | 322 | 146 (45.34) | 176 (54.66) | 115 (35.71) | 207 (64.29) | 49 (35-60) | 0.32 | 0.65 | 2.7 | 3.97 | 2.42 | 2.11 |
| Gastroenteritis due to Rotavirus | 216 | 106 (49.07) | 110 (50.93) | 111 (51.39) | 105 (48.61) | 1 (1-3) | 0.88 | 0.22 | 0.77 | 1.46 | 5.25 | 1.41 |
| Intestinal infections due to *Escherichia coli* | 206 | 103 (50.00) | 103 (50.00) | 21 (10.19) | 185 (89.81) | 48 (17-61) | 0 | 0 | 1.87 | 3.02 | 1.34 | 1.35 |
| Oesophagostomiasis | 156 | 51 (32.69) | 105 (67.31) | 144 (92.31) | 12 (7.69) | 50 (42-58) | 0 | 0 | 0.11 | 2.49 | 2.99 | 1.02 |
| Hepatitis A | 102 | 41 (40.20) | 61 (59.80) | 70 (68.63) | 32 (31.37) | 42 (29-58) | 0.39 | 0.25 | 0.96 | 0.85 | 0.88 | 0.67 |
| Enterobiasis | 82 | 46 (56.10) | 36 (43.90) | 19 (23.17) | 63 (76.83) | 18 (10-43) | 0.18 | 0 | 0.91 | 0.85 | 0.67 | 0.54 |
| Hepatitis E | 78 | 52 (66.67) | 26 (33.33) | 53 (67.95) | 25 (32.05) | 58 (43-65) | 0.53 | 0.65 | 0.6 | 0.29 | 0.51 | 0.51 |
| Bacterial foodborne intoxications, unspecified | 54 | 28 (51.85) | 26 (48.15) | 40 (74.07) | 14 (25.93) | 41 (33-55) | 0.21 | 0.15 | 0.23 | 0.69 | 0.46 | 0.35 |
| Enteritis due to Norovirus | 42 | 20 (47.62) | 22 (52.38) | 17 (40.48) | 25 (59.52) | 49 (28-58) | 0.11 | 0.03 | 0.77 | 0.21 | 0.15 | 0.27 |
| Cysticercosis | 42 | 19 (45.24) | 23 (54.76) | 34 (80.95) | 8 (19.05) | 55 (38-66) | 0.11 | 0.15 | 0.17 | 0.48 | 0.51 | 0.27 |
| Infections due to other *Salmonella* | 33 | 20 (60.61) | 13 (39.39) | 17 (51.52) | 16 (48.48) | 44 (27-64) | 0 | 0.09 | 0.03 | 0.29 | 0.93 | 0.22 |
| Taeniasis | 14 | 5 (35.71) | 9 (64.29) | 9 (64.29) | 5 (35.71) | 39 (14-57) | 0 | 0.03 | 0.09 | 0.13 | 0.26 | 0.09 |
| Campylobacteriosis | 10 | 4 (40.00) | 6 (60.00) | 4 (40.00) | 6 (60.00) | 52 (47-61) | 0.04 | 0.03 | 0.06 | 0.03 | 0.26 | 0.07 |
| Toxoplasmosis | 9 | 3 (33.33) | 6 (66.67) | 4 (44.44) | 5 (55.56) | 40 (39-46) | 0.07 | 0 | 0.06 | 0.11 | 0.05 | 0.06 |
| Clonorchiasis | 9 | 2 (22.22) | 7 (77.78) | 7 (77.78) | 2 (22.22) | 48 (44-61) | 0.07 | 0 | 0 | 0.03 | 0.31 | 0.06 |
| Botulism | 9 | 2 (22.22) | 7 (77.78) | 6 (66.67) | 3 (33.33) | 32 (30-53) | 0 | 0.03 | 0.06 | 0.11 | 0.10 | 0.06 |

| | | | | | | | | | | | | |
|---|---|---|---|---|---|---|---|---|---|---|---|---|
| Paragonimiasis | 7 | 3 (42.86) | 4 (57.14) | 2 (28.57) | 5 (71.43) | 57 (52-66) | 0 | 0 | 0 | 0.19 | 0 | 0.05 |
| Trichuriasis | 6 | 2 (33.33) | 4 (66.67) | 5 (83.33) | 1 (16.67) | 44 (39-48) | 0.04 | 0 | 0 | 0.11 | 0.05 | 0.04 |
| Cryptosporidiosis | 5 | 4 (80.00) | 1 (20.00) | 4 (80.00) | 1 (20.00) | 45 (40-47) | 0 | 0 | 0.03 | 0.08 | 0.05 | 0.03 |
| Capillariasis | 4 | 2 (50.00) | 2 (50.00) | 0 (0.00) | 4 (100.00) | 30 (15-47) | 0 | 0 | 0.11 | 0 | 0 | 0.03 |
| Enteritis due to Adenovirus | 4 | 3 (75.00) | 1 (25.00) | 2 (50.00) | 2 (50.00) | 1 (0-17) | 0.04 | 0.03 | 0.03 | 0.03 | 0 | 0.03 |
| Listeriosis | 3 | 0 (0.00) | 3 (100.00) | 3 (100.00) | 0 (0.00) | 48 (39-57) | 0 | 0 | 0 | 0.05 | 0.05 | 0.02 |
| Epidemic myalgia | 3 | 1 (33.33) | 2 (66.67) | 1 (33.33) | 2 (66.67) | 68 (37-71) | 0 | 0 | 0.03 | 0.05 | 0 | 0.02 |
| Foodborne Bacillus cereus intoxication | 2 | 1 (50.00) | 1 (50.00) | 2 (100.00) | 0 (0.00) | 69 (69-69) | 0 | 0.03 | 0.03 | 0 | 0 | 0.01 |
| Intestinal infections due to Yersinia enterocolitica | 2 | 0 (0.00) | 2 (100.00) | 2 (100.00) | 0 (0.00) | 69 (65-73) | 0 | 0 | 0.06 | 0 | 0 | 0.01 |
| Foodborne Clostridium perfringens intoxication | 2 | 0 (0.00) | 2 (100.00) | 2 (100.00) | 0 (0.00) | 43 (38-47) | 0.07 | 0 | 0 | 0 | 0 | 0.01 |
| Giardiasis | 2 | 1 (50.00) | 1 (50.00) | 2 (100.00) | 0 (0.00) | 48 (40-55) | 0.04 | 0 | 0.03 | 0 | 0 | 0.01 |
| Foodborne staphylococcal intoxication | 1 | 1 (100.00) | 0 (0.00) | 1 (100.00) | 0 (0.00) | 78 (78-78) | 0 | 0.03 | 0 | 0 | 0 | 0.01 |
| Dipylidiasis | 1 | 0 (0.00) | 1 (100.00) | 0 (0.00) | 1 (100.00) | 56 (56-56) | 0 | 0 | 0.03 | 0 | 0 | 0.01 |
| Cystoisosporiasis | 1 | 0 (0.00) | 1 (100.00) | 0 (0.00) | 1 (100.00) | 50 (50-50) | 0.04 | 0 | 0 | 0 | 0 | 0.01 |
| Fascioliasis | 1 | 1 (100.00) | 0 (0.00) | 1 (100.00) | 0 (0.00) | 46 (46-46) | 0.04 | 0 | 0 | 0 | 0 | 0.01 |
| Syngamosis | 1 | 1 (100.00) | 0 (0.00) | 0 (0.00) | 1 (100.00) | 31 (31-31) | 0 | 0 | 0 | 0.03 | 0 | 0.01 |
| **Vector-borne** | 1128 | 613 (54.34) | 515 (45.66) | 780 (69.15) | 348 (30.85) | 44 (31-54) | 2.33 | 3.04 | 5.59 | 12.39 | 15.4 | 7.37 |
| Brucellosis | 640 | 348 (54.38) | 292 (45.62) | 497 (77.66) | 143 (22.34) | 42 (30-50) | 0.39 | 0.90 | 1.48 | 8.32 | 12.05 | 4.18 |
| Hemorrhagic fever | 151 | 103 (68.21) | 48 (31.79) | 68 (45.03) | 83 (54.97) | 49 (43-61) | 0.92 | 0.84 | 1.13 | 1.19 | 0.67 | 0.99 |
| Typhus fever | 49 | 20 (40.82) | 29 (59.18) | 31 (63.27) | 18 (36.73) | 56 (48-63) | 0.18 | 0.28 | 0.40 | 0.40 | 0.31 | 0.32 |
| Orf | 47 | 24 (51.06) | 23 (48.94) | 33 (70.21) | 14 (29.79) | 15 (8-24) | 0.21 | 0.37 | 0.34 | 0.32 | 0.26 | 0.31 |
| Erysipeloid | 45 | 26 (57.78) | 19 (42.22) | 33 (73.33) | 12 (26.67) | 55 (38-64) | 0.18 | 0.15 | 0.43 | 0.45 | 0.15 | 0.29 |
| Severe fever with thrombocytopenia syndrome | 44 | 22 (50.00) | 22 (50.00) | 23 (52.27) | 21 (47.73) | 63 (53-69) | 0.07 | 0.06 | 0.68 | 0.34 | 0.15 | 0.29 |
| Malaria | 37 | 32 (86.49) | 5 (13.51) | 26 (70.27) | 11 (29.73) | 41 (31-47) | 0.07 | 0.19 | 0.28 | 0.34 | 0.31 | 0.24 |
| Spotted fever | 23 | 6 (26.09) | 17 (73.91) | 18 (78.26) | 5 (21.74) | 42 (31-53) | 0 | 0 | 0.31 | 0.13 | 0.36 | 0.15 |
| Tularemia | 20 | 6 (30.00) | 14 (70.00) | 11 (55.00) | 9 (45.00) | 57 (48-64) | 0.04 | 0.06 | 0.14 | 0.11 | 0.41 | 0.13 |

| | | | | | | | | | | | | |
|---|---|---|---|---|---|---|---|---|---|---|---|---|
| Leptospirosis | 10 | 3 (30.00) | 7 (70.00) | 5 (50.00) | 5 (50.00) | 28 (27-29) | 0 | 0 | 0.03 | 0.11 | 0.26 | 0.07 |
| Epidemic encephalitis B | 10 | 4 (40.00) | 6 (60.00) | 9 (90.00) | 1 (10.00) | 52 (38-61) | 0.18 | 0.03 | 0 | 0.11 | 0 | 0.07 |
| Extraintestinal yersiniosis | 9 | 4 (44.44) | 5 (55.56) | 7 (77.78) | 2 (22.22) | 60 (31-62) | 0.04 | 0.06 | 0 | 0.13 | 0.05 | 0.06 |
| Glanders | 9 | 5 (55.56) | 4 (44.44) | 2 (22.22) | 7 (77.78) | 41 (37-59) | 0 | 0 | 0.14 | 0.08 | 0.05 | 0.06 |
| Cat-scratch disease | 5 | 1 (20.00) | 4 (80.00) | 1 (20.00) | 4 (80.00) | 44 (11-53) | 0 | 0 | 0.06 | 0.08 | 0 | 0.03 |
| Relapsing fever | 4 | 1 (25.00) | 3 (75.00) | 3 (75.00) | 1 (25.00) | 62 (47-74) | 0.04 | 0.03 | 0 | 0 | 0.10 | 0.03 |
| Monkeypox | 4 | 2 (50.00) | 2 (50.00) | 3 (75.00) | 1 (25.00) | 46 (39-55) | 0 | 0 | 0 | 0.05 | 0.10 | 0.03 |
| Q fever | 4 | 0 (0.00) | 4 (100.00) | 2 (50.00) | 2 (50.00) | 40 (19-67) | 0 | 0 | 0.06 | 0.03 | 0.05 | 0.03 |
| Yellow fever | 3 | 1 (33.33) | 2 (66.67) | 0 (0.00) | 3 (100.00) | 69 (56-71) | 0 | 0 | 0 | 0.08 | 0 | 0.02 |
| Eastern equine encephalitis | 2 | 0 (0.00) | 2 (100.00) | 0 (0.00) | 2 (100.00) | 69 (69-70) | 0 | 0 | 0.03 | 0 | 0.05 | 0.01 |
| Melioidosis | 2 | 0 (0.00) | 2 (100.00) | 1 (50.00) | 1 (50.00) | 75 (71-78) | 0 | 0 | 0.06 | 0 | 0 | 0.01 |
| African trypanosomiasis | 2 | 1 (50.00) | 1 (50.00) | 1 (50.00) | 1 (50.00) | 67 (61-74) | 0 | 0.03 | 0 | 0 | 0.05 | 0.01 |
| Visceral leishmaniasis | 2 | 2 (100.00) | 0 (0.00) | 1 (50.00) | 1 (50.00) | 29 (28-30) | 0 | 0 | 0 | 0.05 | 0 | 0.01 |
| Dengue | 2 | 1 (50.00) | 1 (50.00) | 1 (50.00) | 1 (50.00) | 21 (16-26) | 0 | 0.03 | 0 | 0.03 | 0 | 0.01 |
| Bartonellosis | 1 | 0 (0.00) | 1 (100.00) | 1 (100.00) | 0 (0.00) | 53 (53-53) | 0 | 0 | 0 | 0.03 | 0 | 0.01 |
| Crimean-Congo haemorrhagic fever | 1 | 0 (0.00) | 1 (100.00) | 1 (100.00) | 0 (0.00) | 41 (41-41) | 0 | 0 | 0.03 | 0 | 0 | 0.01 |
| Rickettsialpox | 1 | 0 (0.00) | 1 (100.00) | 1 (100.00) | 0 (0.00) | 35 (35-35) | 0 | 0 | 0 | 0.03 | 0 | 0.01 |
| Lyme borreliosis | 1 | 1 (100.00) | 0 (0.00) | 1 (100.00) | 0 (0.00) | 28 (28-28) | 0.04 | 0 | 0 | 0 | 0 | 0.01 |
| **Blood- and sexually transmitted** | 10170 | 5285 (51.97) | 4885 (48.03) | 6971 (68.54) | 3199 (31.46) | 43 (31-55) | 46.2 | 46.17 | 63.67 | 76.59 | 115.23 | 66.48 |
| Hepatitis B | 6576 | 4091 (62.21) | 2485 (37.79) | 4487 (68.23) | 2089 (31.77) | 46 (35-57) | 38.00 | 36.77 | 48.28 | 45.08 | 47.23 | 43.03 |
| Syphilis | 1667 | 254 (15.24) | 1413 (84.76) | 1269 (76.12) | 398 (23.88) | 31 (27-38) | 1.17 | 2.01 | 3.12 | 14.88 | 46.19 | 10.9 |
| Hepatitis C | 517 | 337 (65.18) | 180 (34.82) | 403 (77.95) | 114 (22.05) | 50 (38-60) | 3.96 | 3.13 | 3.55 | 3.39 | 2.63 | 3.38 |
| Gonorrhea | 433 | 263 (60.74) | 170 (39.26) | 222 (51.27) | 211 (48.73) | 43 (28-58) | 0.6 | 0.43 | 3.01 | 4.95 | 5.61 | 2.83 |
| Trichomoniasis | 394 | 9 (2.28) | 385 (97.72) | 251 (63.71) | 143 (36.29) | 36 (28-45) | 1.49 | 1.55 | 2.50 | 3.20 | 4.79 | 2.58 |
| Anogenital warts | 234 | 103 (44.02) | 131 (55.98) | 146 (62.39) | 88 (37.61) | 30 (25-45) | 0.25 | 0.71 | 1.16 | 2.70 | 3.14 | 1.53 |
| Cytomegaloviral disease | 183 | 100 (54.64) | 83 (45.36) | 80 (43.72) | 103 (56.28) | 2 (0-32) | 0.25 | 1.08 | 1.25 | 1.40 | 2.27 | 1.20 |

| | | | | | | | | | | | | |
|---|---|---|---|---|---|---|---|---|---|---|---|---|
| HIV infection | 89 | 75 (84.27) | 14 (15.73) | 64 (71.91) | 25 (28.09) | 35 (28-48) | 0.25 | 0.12 | 0.28 | 0.34 | 2.83 | 0.58 |
| Anogenital herpes simplex infection | 53 | 37 (69.81) | 16 (30.19) | 34 (64.15) | 19 (35.85) | 51 (38-54) | 0.14 | 0.31 | 0.45 | 0.48 | 0.26 | 0.35 |
| Granuloma inguinale | 12 | 8 (66.67) | 4 (33.33) | 4 (33.33) | 8 (66.67) | 49 (41-53) | 0 | 0.03 | 0.06 | 0.13 | 0.21 | 0.08 |
| Hepatitis D | 9 | 7 (77.78) | 2 (22.22) | 9 (100.00) | 0 (0.00) | 61 (45-62) | 0.04 | 0.03 | 0.06 | 0.05 | 0.15 | 0.06 |
| Chancroid | 2 | 1 (50.00) | 1 (50.00) | 2 (100.00) | 0 (0.00) | 48 (44-51) | 0.07 | 0 | 0 | 0 | 0 | 0.01 |
| Warts of lips or oral cavity | 1 | 0 (0.00) | 1 (100.00) | 0 (0.00) | 1 (100.00) | 4 (4-4) | 0 | 0 | 0 | 0.03 | 0 | 0.01 |
| **Mucocutaneous** | 23178 | 11435 (49.34) | 11743 (50.66) | 15500 (66.87) | 7678 (33.13) | 44 (14-62) | 72.8 | 104.06 | 156.13 | 209.43 | 224.03 | 151.52 |
| Zoster | 10636 | 4902 (46.09) | 5734 (53.91) | 7437 (69.92) | 3199 (30.08) | 58 (44-68) | 28.76 | 36.91 | 73.71 | 100.29 | 115.7 | 69.53 |
| Hand, foot, and mouth disease | 5188 | 3158 (60.87) | 2030 (39.13) | 2466 (47.53) | 2722 (52.47) | 2 (1-4) | 19.14 | 43.66 | 39.13 | 39.06 | 19.77 | 33.92 |
| Viral conjunctivitis | 1914 | 813 (42.48) | 1101 (57.52) | 1739 (90.86) | 175 (9.14) | 56 (42-65) | 7.64 | 6.82 | 12.94 | 17.27 | 19.05 | 12.51 |
| Trachoma | 1176 | 478 (40.65) | 698 (59.35) | 934 (79.42) | 242 (20.58) | 57 (43-68) | 9.98 | 6.20 | 7.18 | 6.99 | 9.11 | 7.69 |
| Common warts | 1156 | 645 (55.8) | 511 (44.20) | 805 (69.64) | 351 (30.36) | 34 (27-47) | 0.71 | 1.98 | 5.42 | 13.27 | 19.57 | 7.56 |
| Herpes simplex infections | 718 | 326 (45.40) | 392 (54.60) | 521 (72.56) | 197 (27.44) | 43 (29-59) | 2.12 | 2.39 | 3.97 | 7.04 | 9.01 | 4.69 |
| Plane warts | 487 | 187 (38.40) | 300 (61.60) | 321 (65.91) | 166 (34.09) | 32 (27-41) | 0.71 | 0.84 | 2.67 | 5.27 | 7.57 | 3.18 |
| Impetigo | 479 | 204 (42.59) | 275 (57.41) | 288 (60.13) | 191 (39.87) | 33 (10-52) | 1.27 | 1.49 | 2.58 | 5.35 | 5.25 | 3.13 |
| Non-dermatophyte superficial dermatomycoses | 339 | 170 (50.15) | 169 (49.85) | 244 (71.98) | 95 (28.02) | 33 (26-46) | 0.50 | 0.74 | 1.84 | 4.24 | 3.91 | 2.22 |
| Molluscum contagiosum | 274 | 101 (36.86) | 173 (63.14) | 194 (70.8) | 80 (29.20) | 29 (25-37) | 0.35 | 0.31 | 0.94 | 2.86 | 5.82 | 1.79 |
| Infectious mononucleosis | 243 | 129 (53.09) | 114 (46.91) | 181 (74.49) | 62 (25.51) | 6 (3-24) | 0.81 | 1.92 | 1.79 | 1.69 | 1.60 | 1.59 |
| Scabies | 167 | 107 (64.07) | 60 (35.93) | 99 (59.28) | 68 (40.72) | 28 (21-45) | 0.11 | 0.31 | 1.13 | 1.46 | 3.04 | 1.09 |
| PIPSM | 155 | 92 (59.35) | 63 (40.65) | 84 (54.19) | 71 (45.81) | 2 (1-3) | 0.25 | 0.12 | 1.05 | 2.12 | 1.39 | 1.01 |
| Myiasis | 72 | 43 (59.72) | 29 (40.28) | 66 (91.67) | 6 (8.33) | 38 (26-54) | 0.04 | 0.03 | 0.51 | 0.82 | 1.08 | 0.47 |
| Tetanus | 35 | 13 (37.14) | 22 (62.86) | 25 (71.43) | 10 (28.57) | 45 (27-53) | 0.18 | 0.15 | 0.17 | 0.45 | 0.10 | 0.23 |
| Rabies | 29 | 19 (65.52) | 10 (34.48) | 11 (37.93) | 18 (62.07) | 29 (7-46) | 0 | 0 | 0.60 | 0.11 | 0.21 | 0.19 |
| Yaws | 29 | 13 (44.83) | 16 (55.17) | 24 (82.76) | 5 (17.24) | 50 (31-62) | 0 | 0 | 0.06 | 0.21 | 0.98 | 0.19 |
| Acute hemorrhagic conjunctivitis | 13 | 8 (61.54) | 5 (38.46) | 11 (84.62) | 2 (15.38) | 40 (34-50) | 0.11 | 0 | 0.09 | 0.05 | 0.26 | 0.08 |
| Eumycetoma | 11 | 4 (36.36) | 7 (63.64) | 11 (100.00) | 0 (0.00) | 49 (42-75) | 0.11 | 0 | 0.06 | 0.13 | 0.05 | 0.07 |

| | | | | | | | | | | | | |
|---|---|---|---|---|---|---|---|---|---|---|---|---|
| Infestation by mites | 11 | 5 (45.45) | 6 (54.55) | 7 (63.64) | 4 (36.36) | 40 (24-61) | 0.04 | 0.03 | 0.06 | 0.11 | 0.15 | 0.07 |
| Infantile papular acrodermatitis | 9 | 5 (55.56) | 4 (44.44) | 5 (55.56) | 4 (44.44) | 3 (2-12) | 0 | 0 | 0.09 | 0.11 | 0.1 | 0.06 |
| Echinococcosis | 8 | 4 (50.00) | 4 (50.00) | 6 (75.00) | 2 (25.00) | 58 (42-61) | 0 | 0.03 | 0.06 | 0.11 | 0.05 | 0.05 |
| Sporotrichosis | 7 | 1 (14.29) | 6 (85.71) | 4 (57.14) | 3 (42.86) | 51 (47-66) | 0 | 0 | 0 | 0.08 | 0.21 | 0.05 |
| Strongyloidiasis | 5 | 3 (60.00) | 2 (40.00) | 3 (60.00) | 2 (40.00) | 68 (62-75) | 0 | 0.06 | 0 | 0.08 | 0 | 0.03 |
| Obstetrical tetanus | 5 | 0 (0.00) | 5 (100.00) | 5 (100.00) | 0 (0.00) | 27 (24-30) | 0 | 0 | 0 | 0.13 | 0 | 0.03 |
| Hookworm diseases | 4 | 1 (25.00) | 3 (75.00) | 3 (75.00) | 1 (25.00) | 51 (44-56) | 0 | 0 | 0.09 | 0 | 0.05 | 0.03 |
| Gas gangrene | 3 | 2 (66.67) | 1 (33.33) | 3 (100.00) | 0 (0.00) | 60 (55-70) | 0 | 0 | 0.03 | 0.05 | 0 | 0.02 |
| Acanthamoebiasis | 3 | 2 (66.67) | 1 (33.33) | 2 (66.67) | 1 (33.33) | 30 (27-33) | 0 | 0.03 | 0 | 0.05 | 0 | 0.02 |
| External hirudiniasis | 1 | 0 (0.00) | 1 (100.00) | 1 (100.00) | 0 (0.00) | 69 (69-69) | 0 | 0.03 | 0 | 0 | 0 | 0.01 |
| Phaeohyphomycosis | 1 | 0 (0.00) | 1 (100.00) | 0 (0.00) | 1 (100.00) | 40 (40-40) | 0 | 0 | 0 | 0.03 | 0 | 0.01 |

*Total number of cases during January 2013 to June 2017.

†Infectious diarrhoeal diseases other than cholera, bacterial dysentery, amoebic dysentery, typhoid, and paratyphoid.

HIV=human immunodeficiency virus, PIPSM=picornavirus infections presenting in the skin or mucous membranes.

**Respiratory**

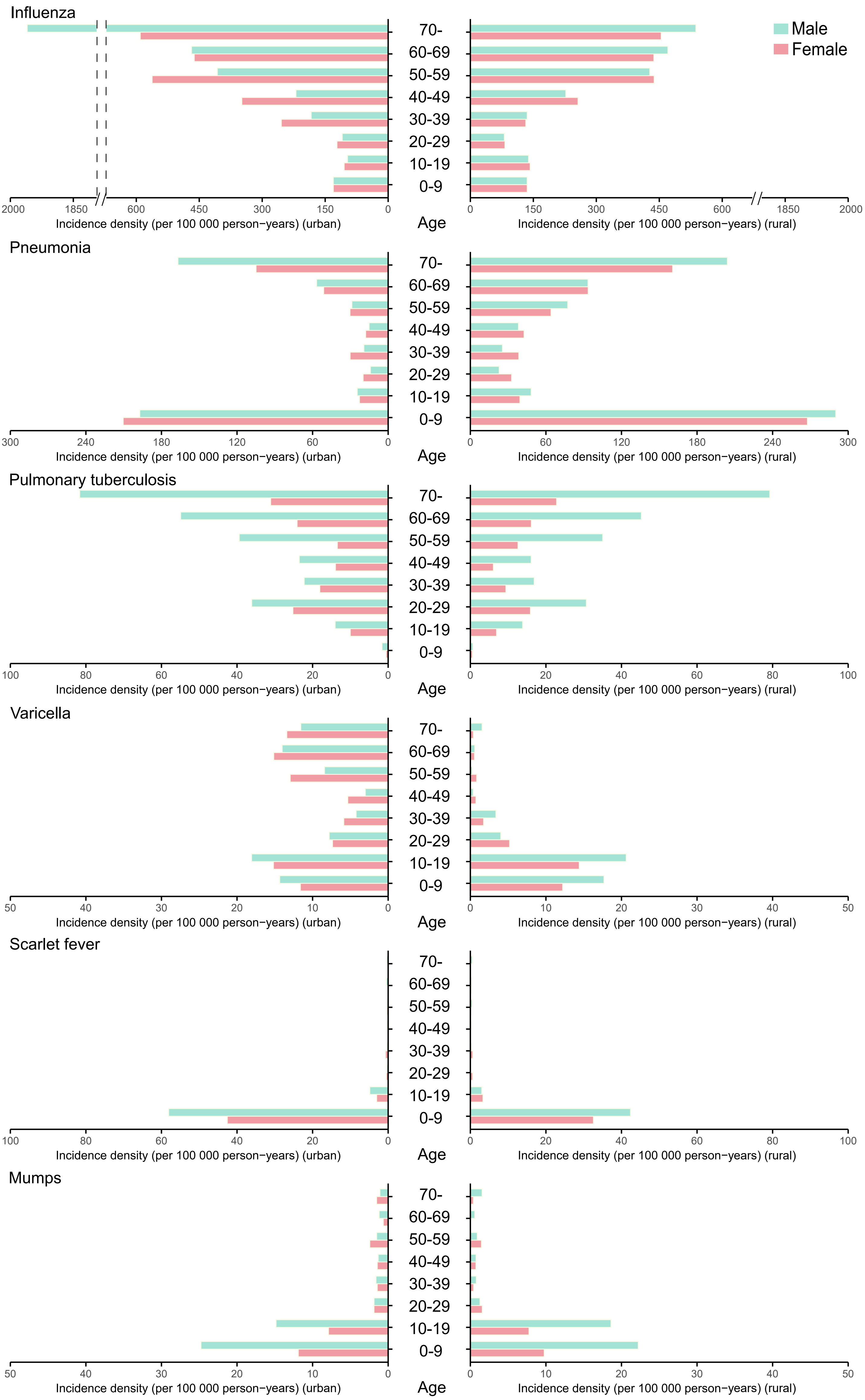

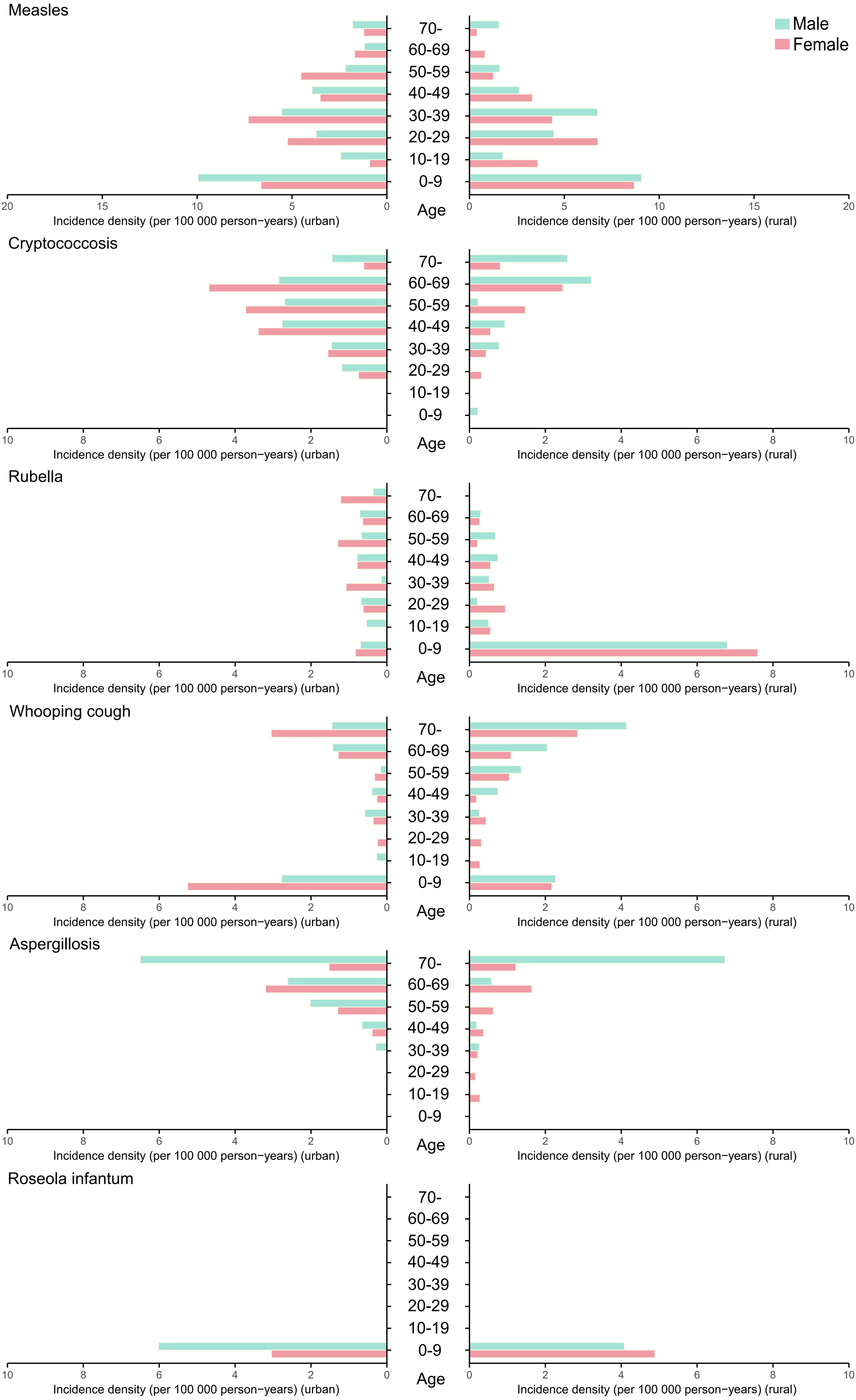
Measles
Male
Female
70-
60-69
50-59
40-49
30-39
20-29
10-19
0-9
Age
20
15
10
5
0
Incidence density (per 100 000 person−years) (urban)
Incidence density (per 100 000 person−years) (rural)
Cryptococcosis
Rubella
Whooping cough
Aspergillosis
Roseola infantum
10
8
6
4
2
0

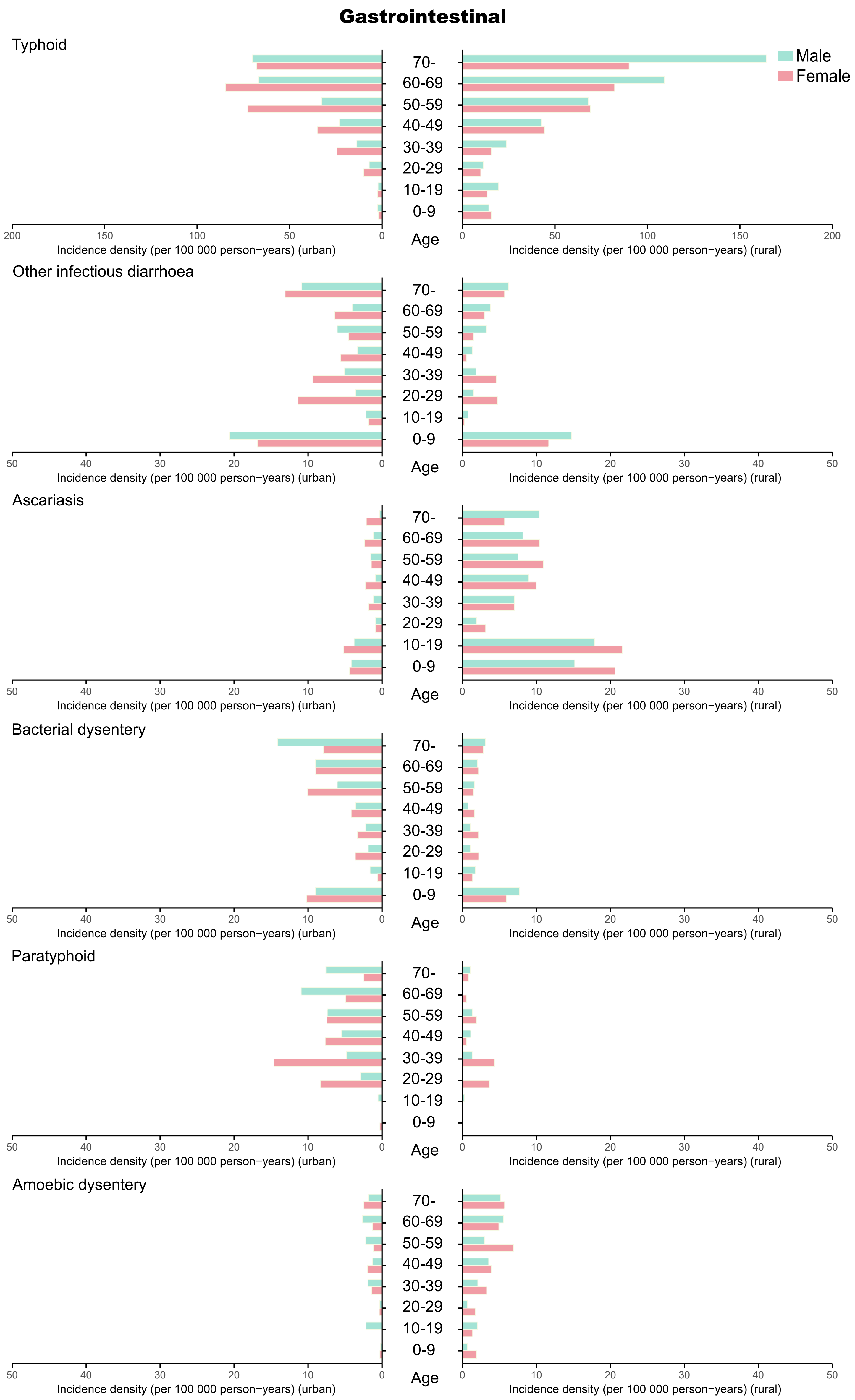
Gastrointestinal
Male
Female
Typhoid
Other infectious diarrhoea
Ascariasis
Bacterial dysentery
Paratyphoid
Amoebic dysentery
70-
60-69
50-59
40-49
30-39
20-29
10-19
0-9
Age
Incidence density (per 100 000 person−years) (urban)
Incidence density (per 100 000 person−years) (rural)
200 150 100 50 0
0 50 100 150 200
50 40 30 20 10 0
0 10 20 30 40 50

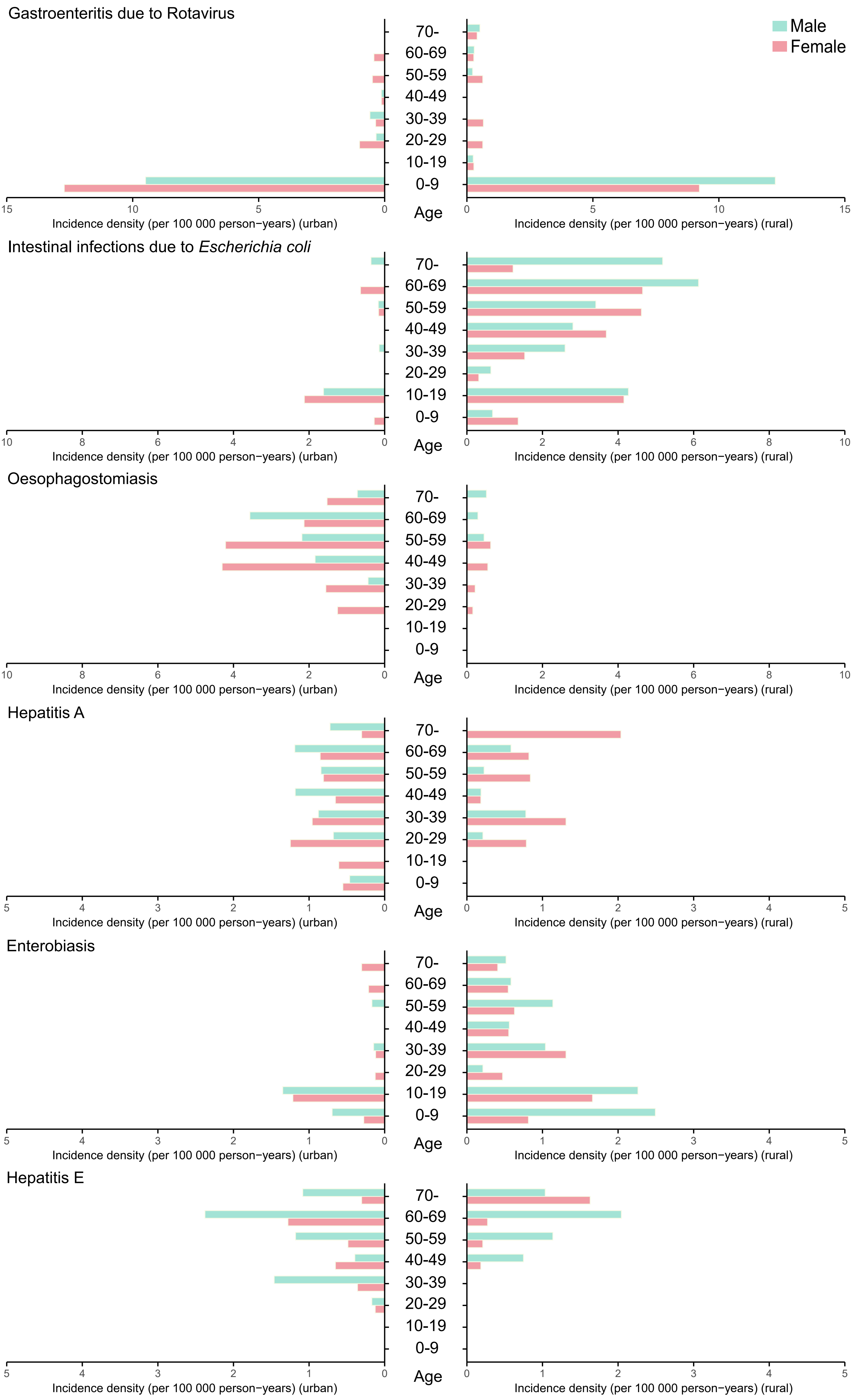
Gastroenteritis due to Rotavirus
Intestinal infections due to *Escherichia coli*
Oesophagostomiasis
Hepatitis A
Enterobiasis
Hepatitis E
Male
Female
70-
60-69
50-59
40-49
30-39
20-29
10-19
0-9
Age
Incidence density (per 100 000 person−years) (urban)
Incidence density (per 100 000 person−years) (rural)

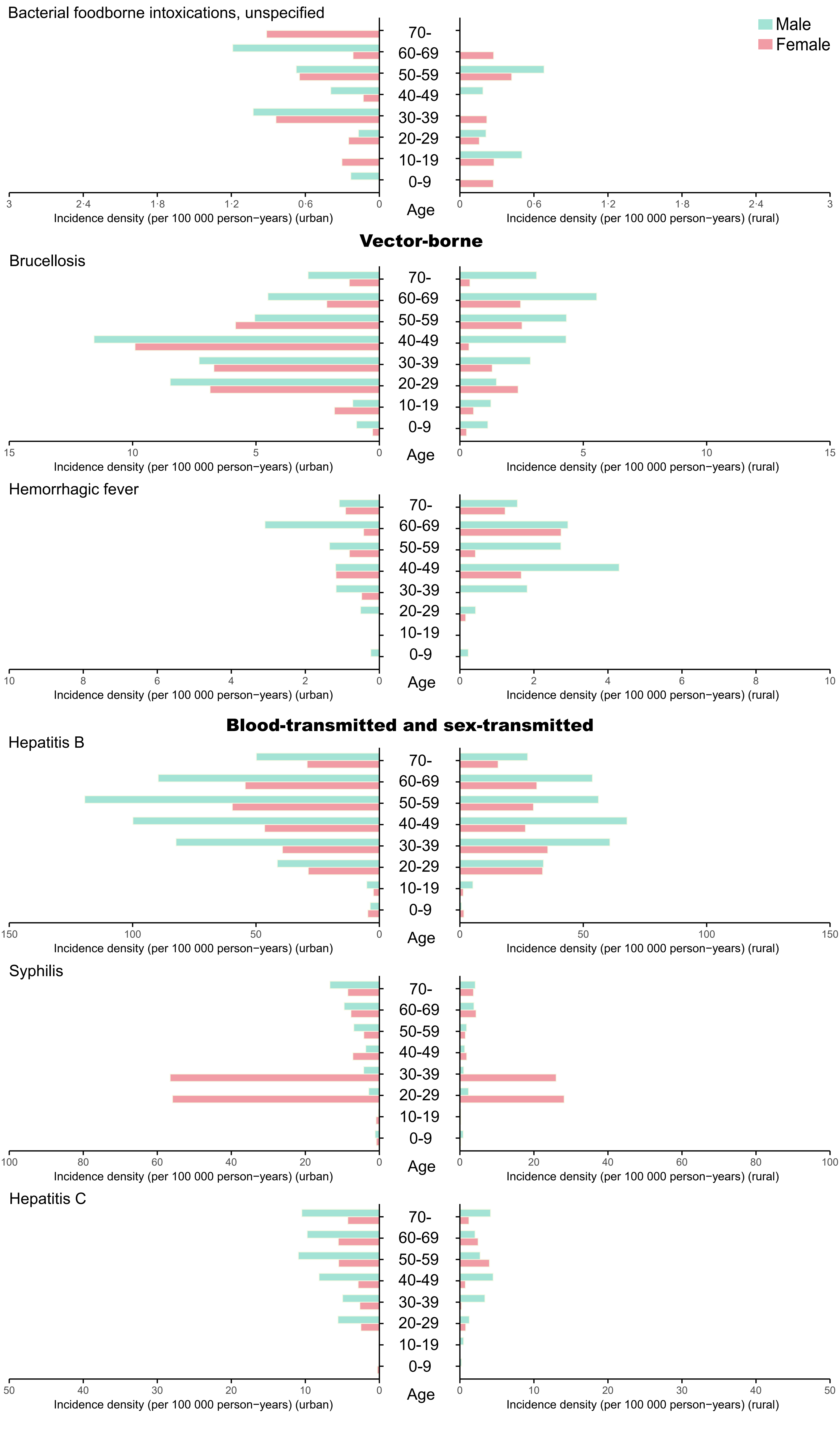

Bacterial foodborne intoxications, unspecified
Male
Female
70-
60-69
50-59
40-49
30-39
20-29
10-19
0-9
Age
3 2·4 1·8 1·2 0·6 0
Incidence density (per 100 000 person−years) (urban)
0 0·6 1·2 1·8 2·4 3
Incidence density (per 100 000 person−years) (rural)
Vector-borne
Brucellosis
15 10 5 0
0 5 10 15
Hemorrhagic fever
10 8 6 4 2 0
0 2 4 6 8 10
Blood-transmitted and sex-transmitted
Hepatitis B
150 100 50 0
0 50 100 150
Syphilis
100 80 60 40 20 0
0 20 40 60 80 100
Hepatitis C
50 40 30 20 10 0
0 10 20 30 40 50

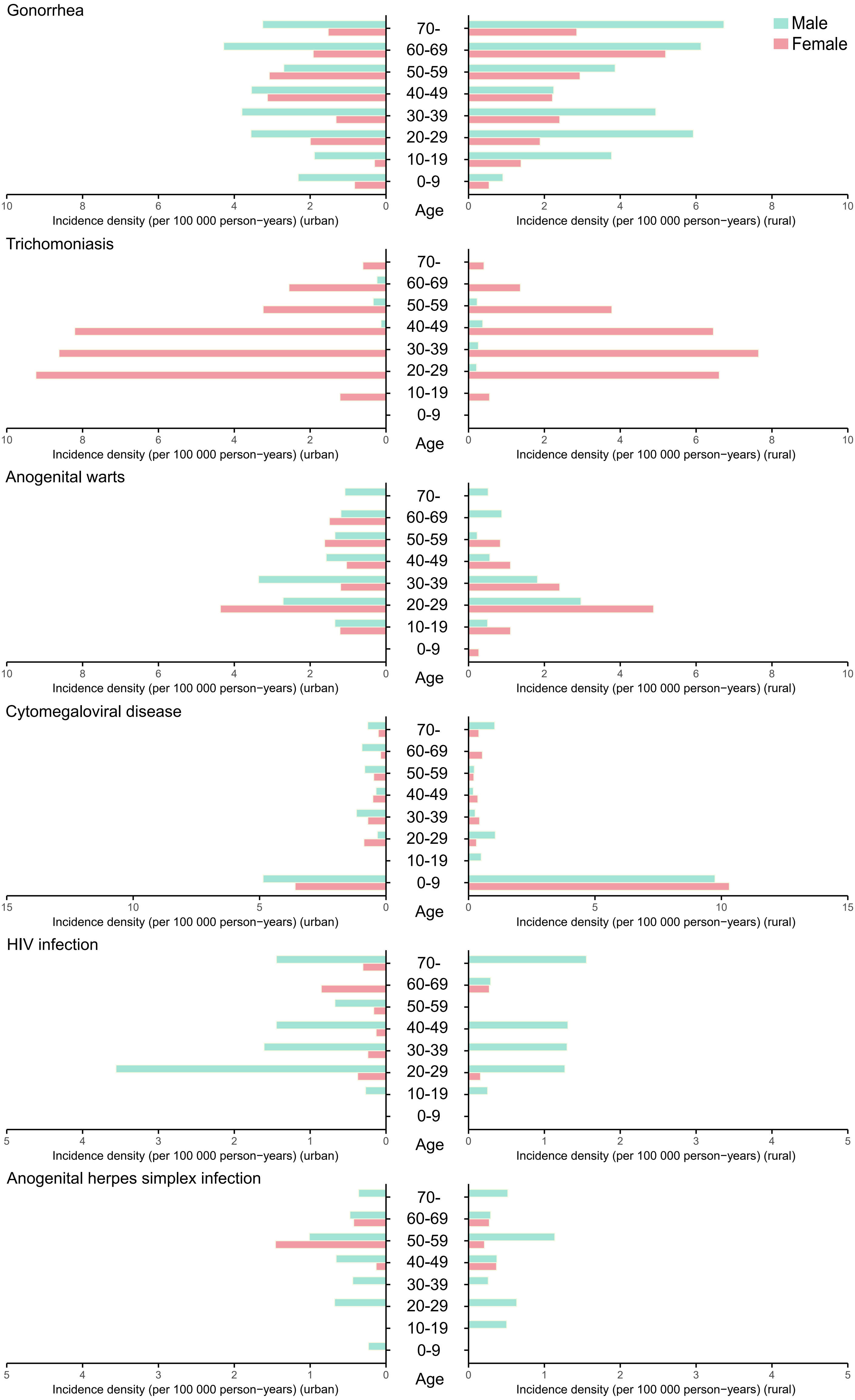
Gonorrhea
Male
Female
Trichomoniasis
Anogenital warts
Cytomegaloviral disease
HIV infection
Anogenital herpes simplex infection
70-
60-69
50-59
40-49
30-39
20-29
10-19
0-9
Age
Incidence density (per 100 000 person−years) (urban)
Incidence density (per 100 000 person−years) (rural)
10
8
6
4
2
0
15
5
3
1

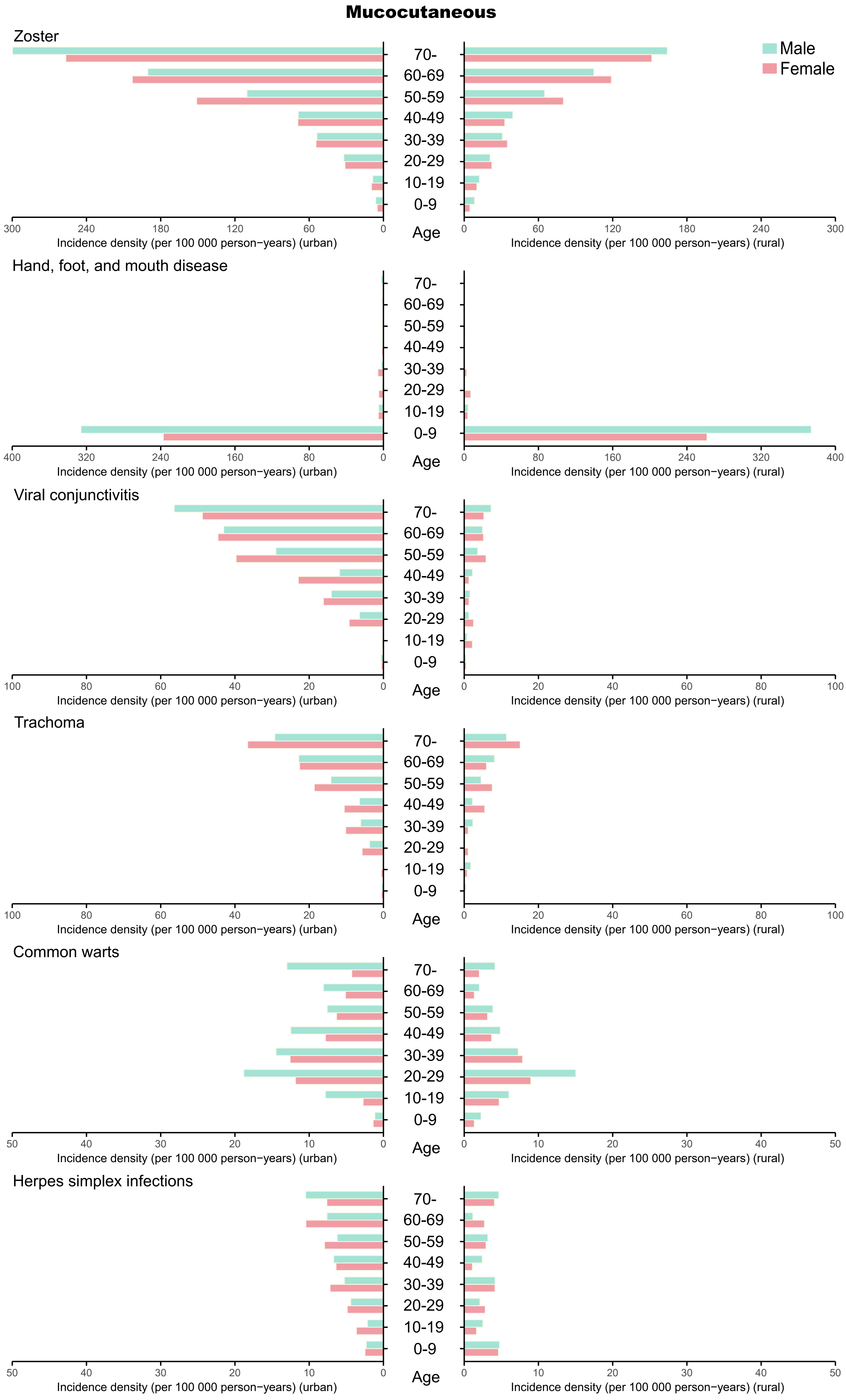
Mucocutaneous
Zoster
Male
Female
70-
60-69
50-59
40-49
30-39
20-29
10-19
0-9
Age
300 240 180 120 60 0
0 60 120 180 240 300
Incidence density (per 100 000 person−years) (urban)
Incidence density (per 100 000 person−years) (rural)
Hand, foot, and mouth disease
400 320 240 160 80 0
0 80 160 240 320 400
Viral conjunctivitis
100 80 60 40 20 0
0 20 40 60 80 100
Trachoma
Common warts
50 40 30 20 10 0
0 10 20 30 40 50
Herpes simplex infections

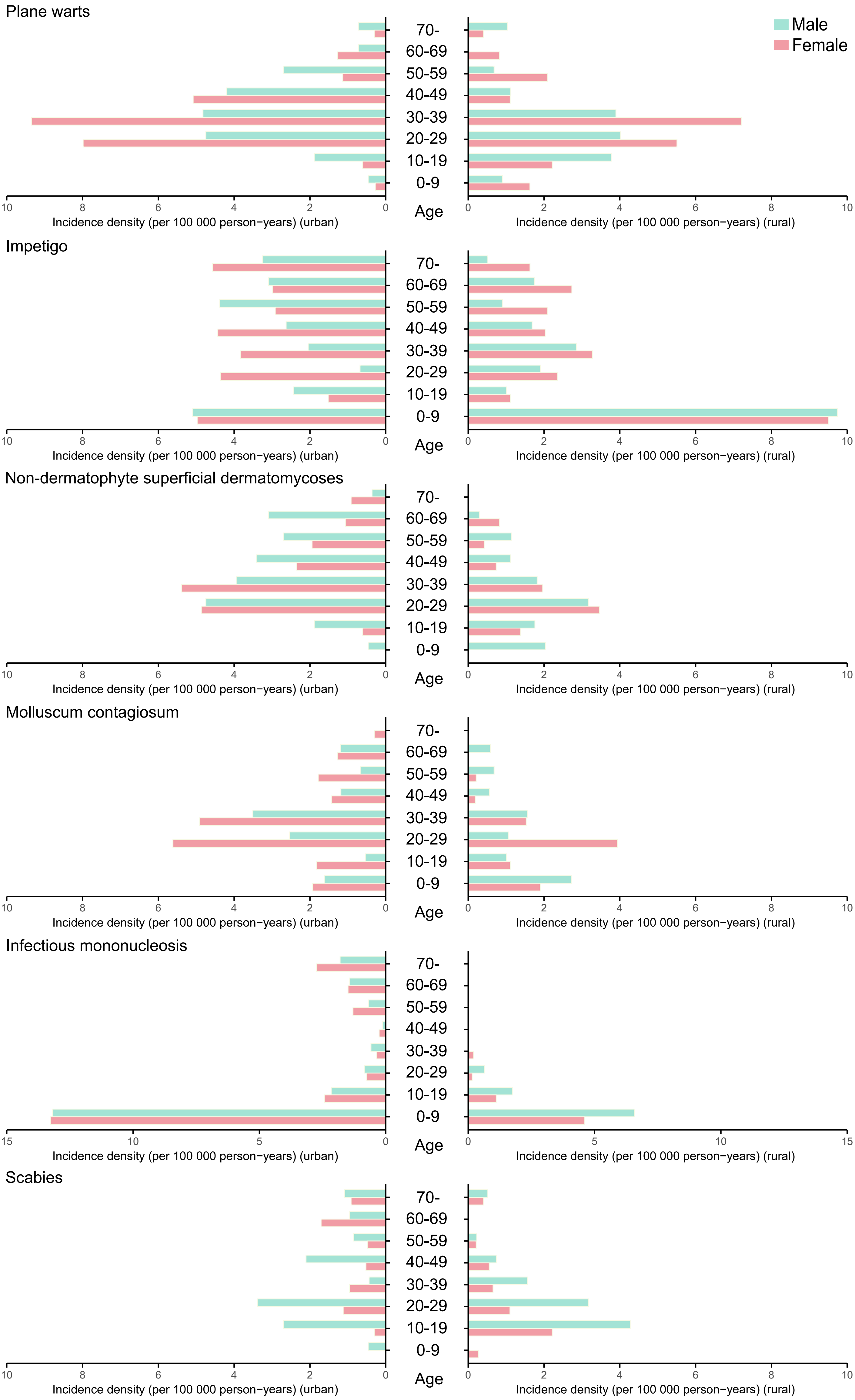
Plane warts
Impetigo
Non-dermatophyte superficial dermatomycoses
Molluscum contagiosum
Infectious mononucleosis
Scabies
Male
Female
70-
60-69
50-59
40-49
30-39
20-29
10-19
0-9
Age
Incidence density (per 100 000 person−years) (urban)
Incidence density (per 100 000 person−years) (rural)

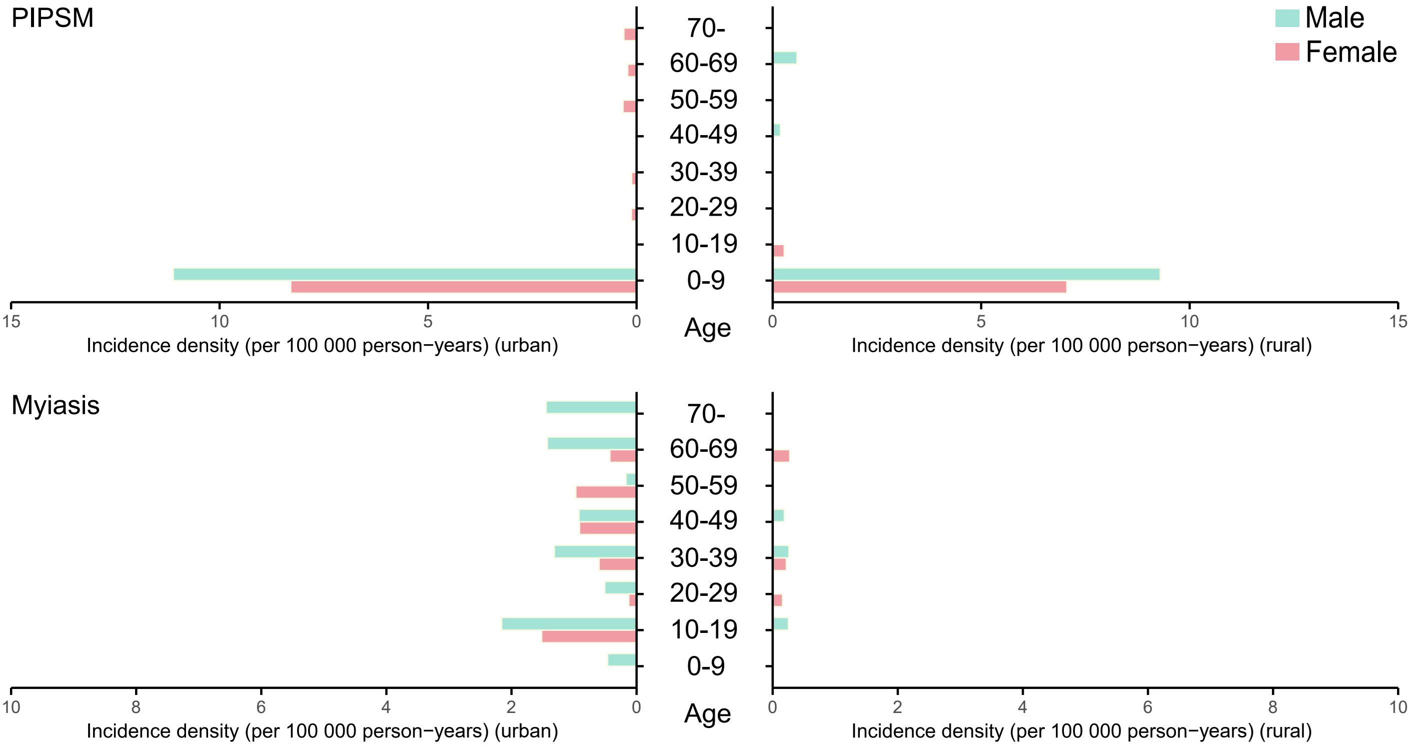


**Figure S2: Age and sex differences related to the incidence density of the top 50 infectious diseases across different geographical regions (urban vs. rural dimension).**
All infectious diseases with more than 50 cases (50 out of 130 infectious diseases) are shown.
HIV=human immunodeficiency virus, PIPSM=picornavirus infections presenting in the skin or mucous membranes.

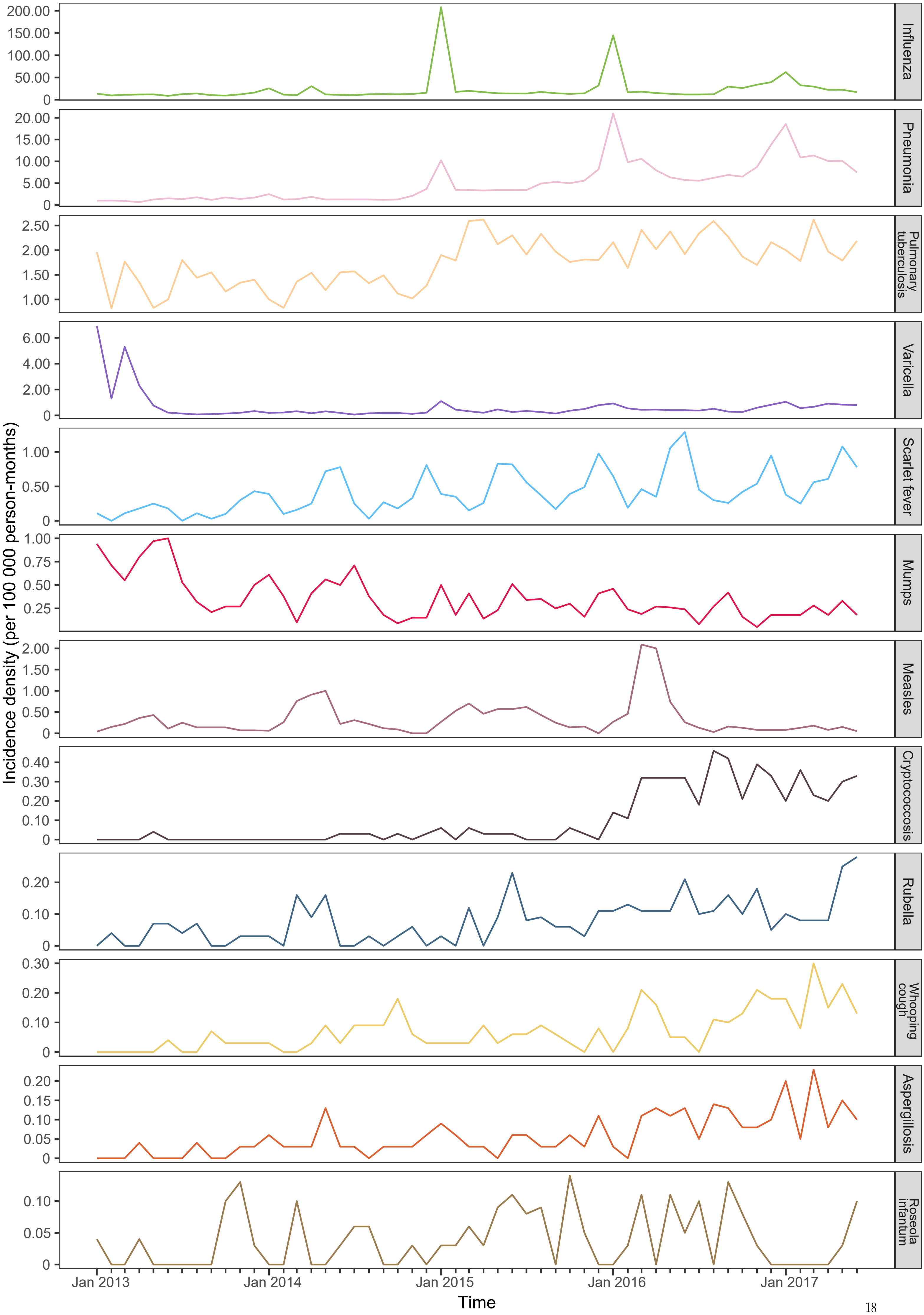
Influenza
Pneumonia
Pulmonary tuberculosis
Varicella
Scarlet fever
Mumps
Measles
Cryptococcosis
Rubella
Whooping cough
Aspergillosis
Roseola infantum
Incidence density (per 100 000 person-months)
Jan 2013
Jan 2014
Jan 2015
Jan 2016
Jan 2017
Time

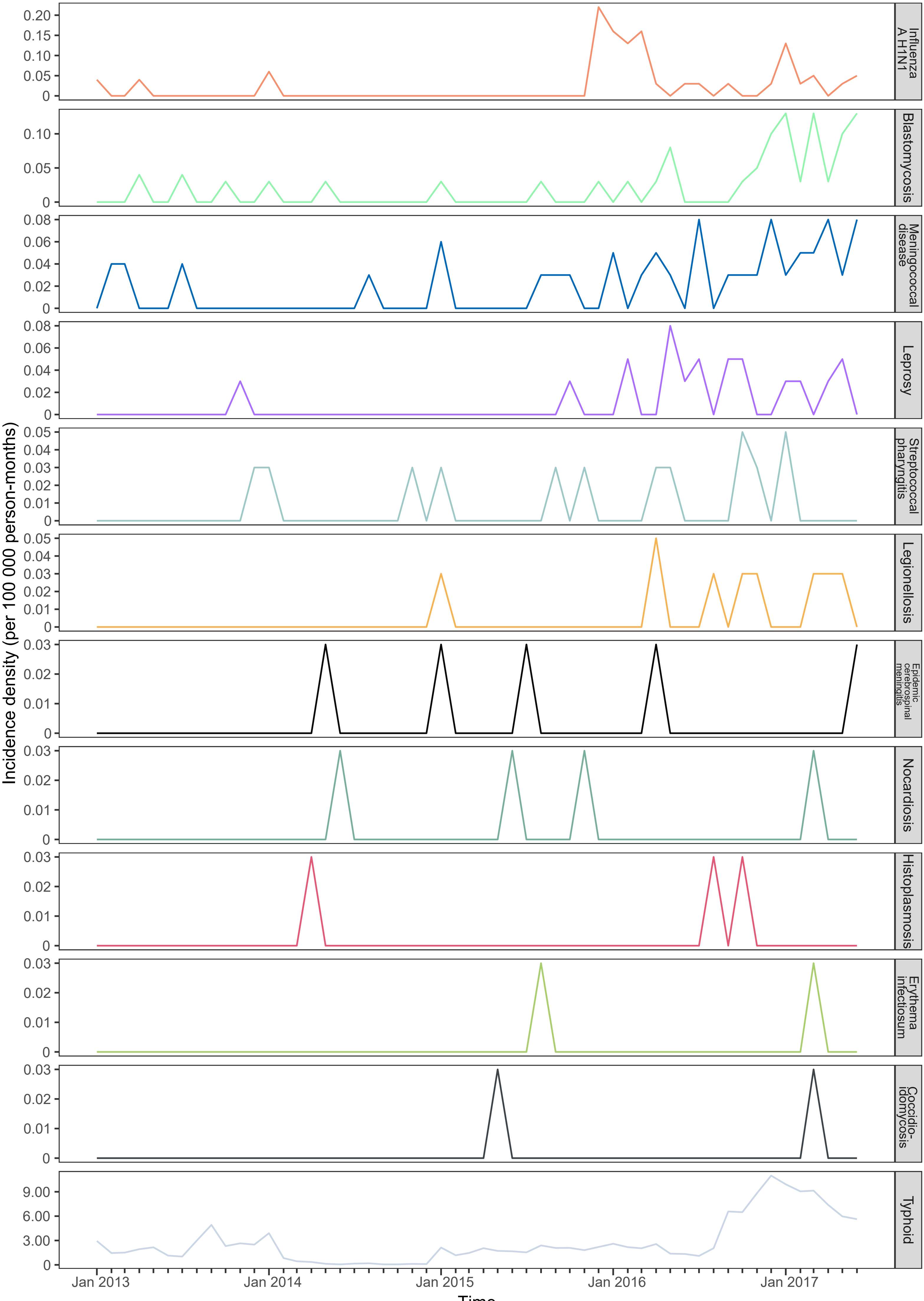

Influenza A H1N1
Blastomycosis
Meningococcal disease
Leprosy
Streptococcal pharyngitis
Legionellosis
Epidemic cerebrospinal meningitis
Nocardiosis
Histoplasmosis
Erythema infectiosum
Coccidio-idomycosis
Typhoid
Incidence density (per 100 000 person-months)
Time
Jan 2013
Jan 2014
Jan 2015
Jan 2016
Jan 2017

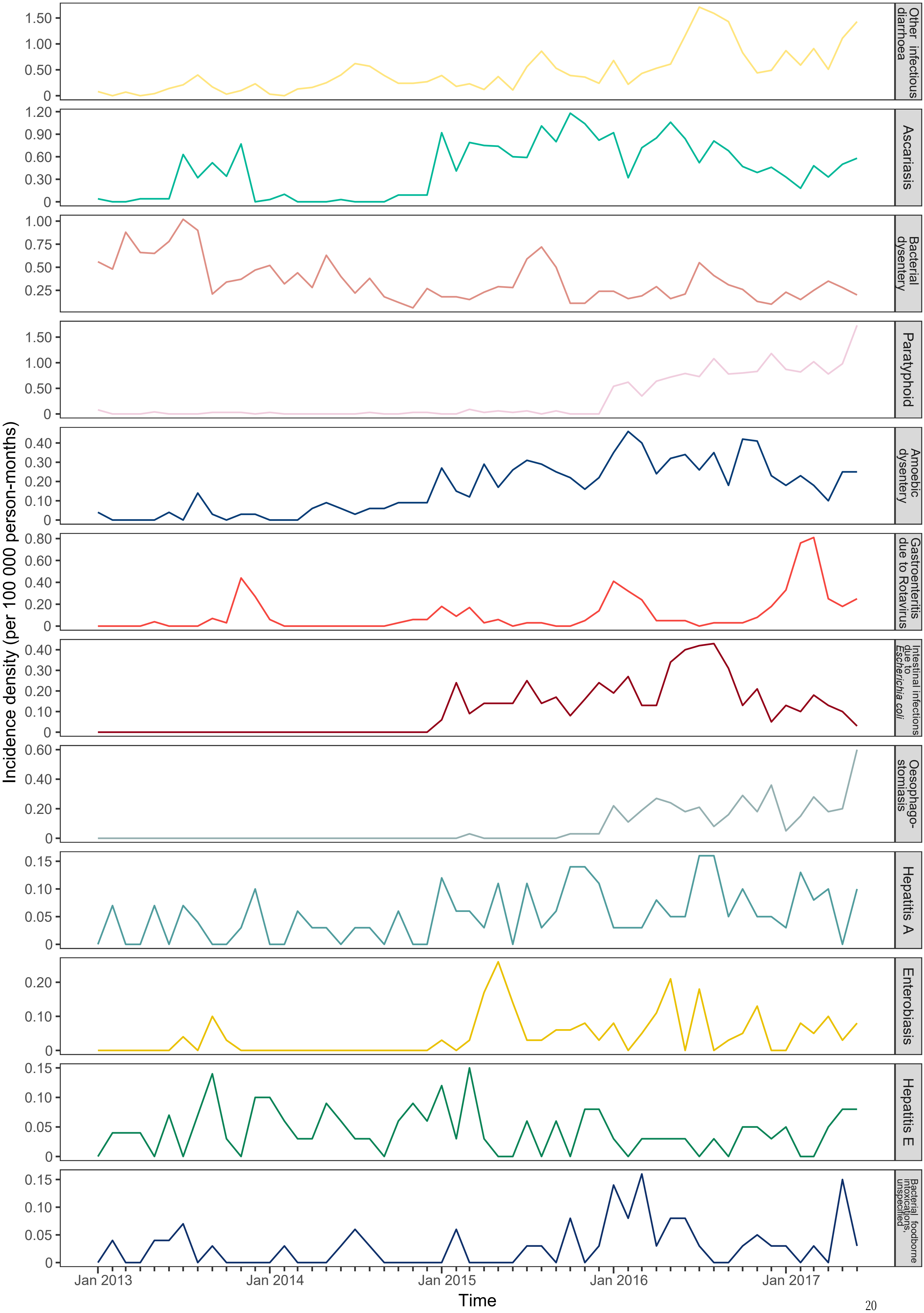
Other infectious diarrhoea
Ascariasis
Bacterial dysentery
Paratyphoid
Amoebic dysentery
Gastroenteritis due to Rotavirus
Intestinal infections due to Escherichia coli
Oesophago-stomiasis
Hepatitis A
Enterobiasis
Hepatitis E
Bacterial foodborne intoxications, unspecified
Incidence density (per 100 000 person-months)
Jan 2013
Jan 2014
Jan 2015
Jan 2016
Jan 2017
Time

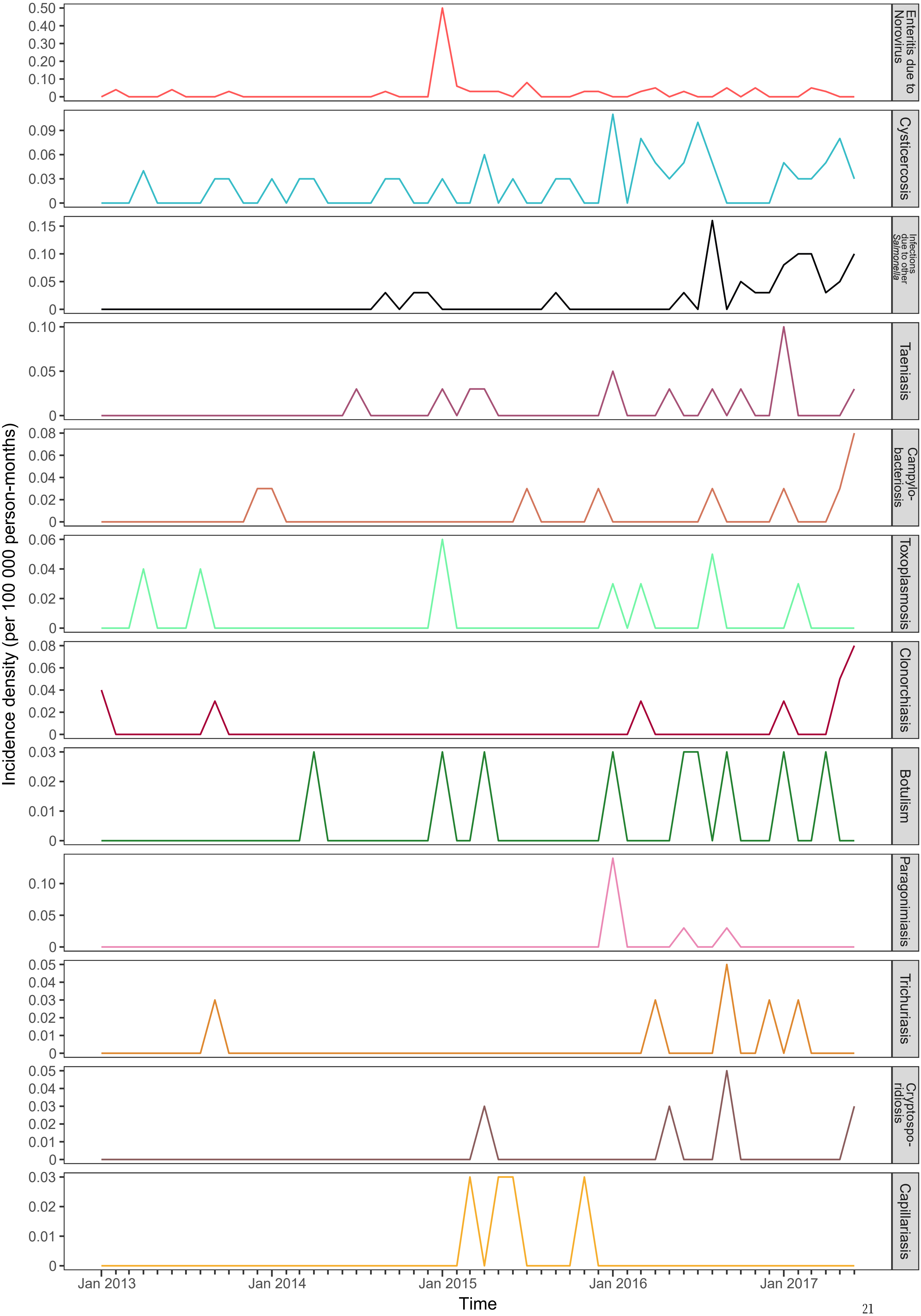
Enteritis due to Norovirus
Cysticercosis
Infections due to other *Salmonella*
Taeniasis
Campylo-bacteriosis
Toxoplasmosis
Clonorchiasis
Botulism
Paragonimiasis
Trichuriasis
Cryptospo-ridiosis
Capillariasis
Incidence density (per 100 000 person-months)
Jan 2013
Jan 2014
Jan 2015
Jan 2016
Jan 2017
Time

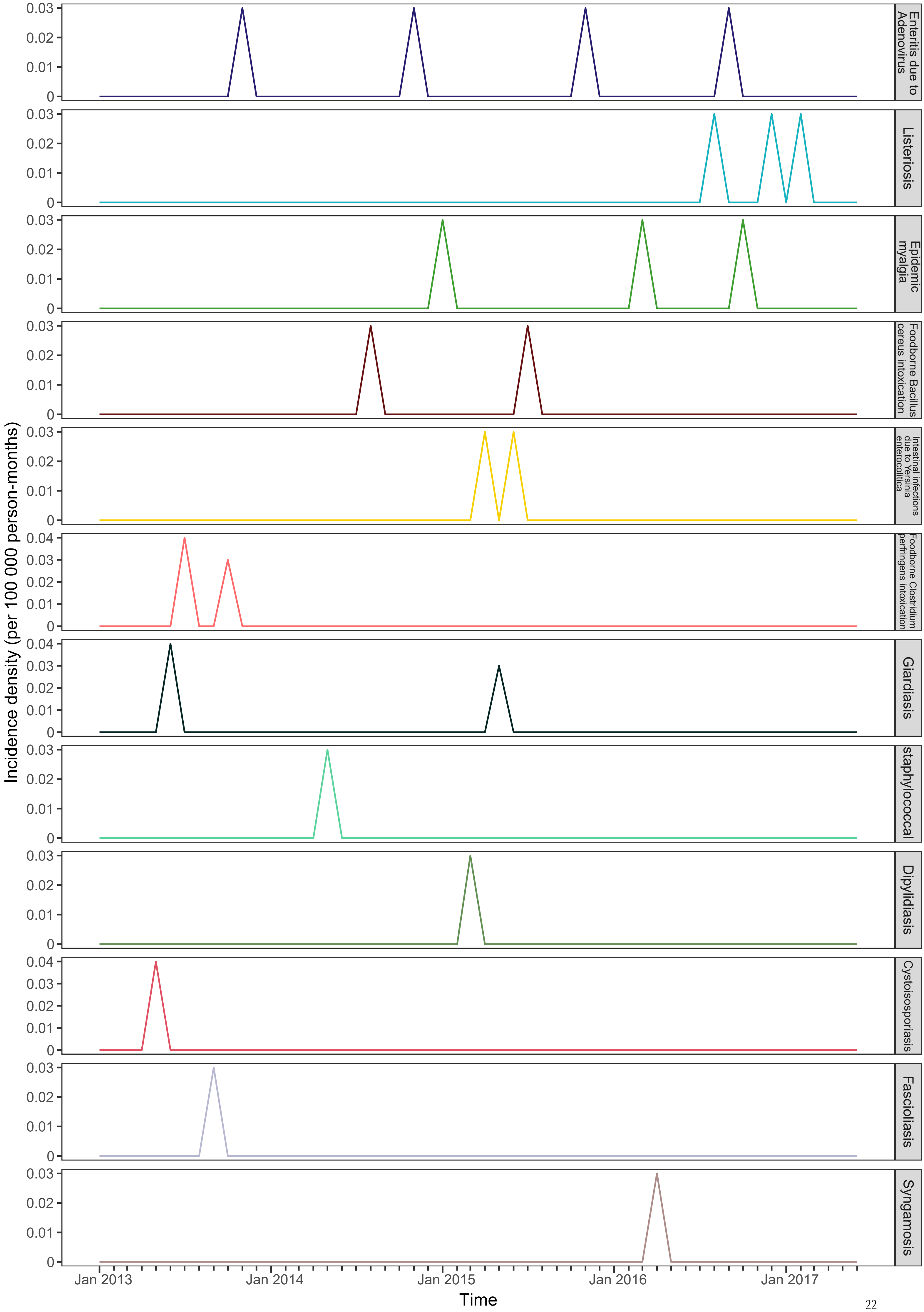

Enteritis due to Adenovirus
Listeriosis
Epidemic myalgia
Foodborne Bacillus cereus intoxication
Intestinal infections due to Yersinia enterocolitica
Foodborne Clostridium perfringens intoxication
Giardiasis
staphylococcal
Dipylidiasis
Cystoisosporiasis
Fascioliasis
Syngamosis
Incidence density (per 100 000 person-months)
Time
Jan 2013
Jan 2014
Jan 2015
Jan 2016
Jan 2017

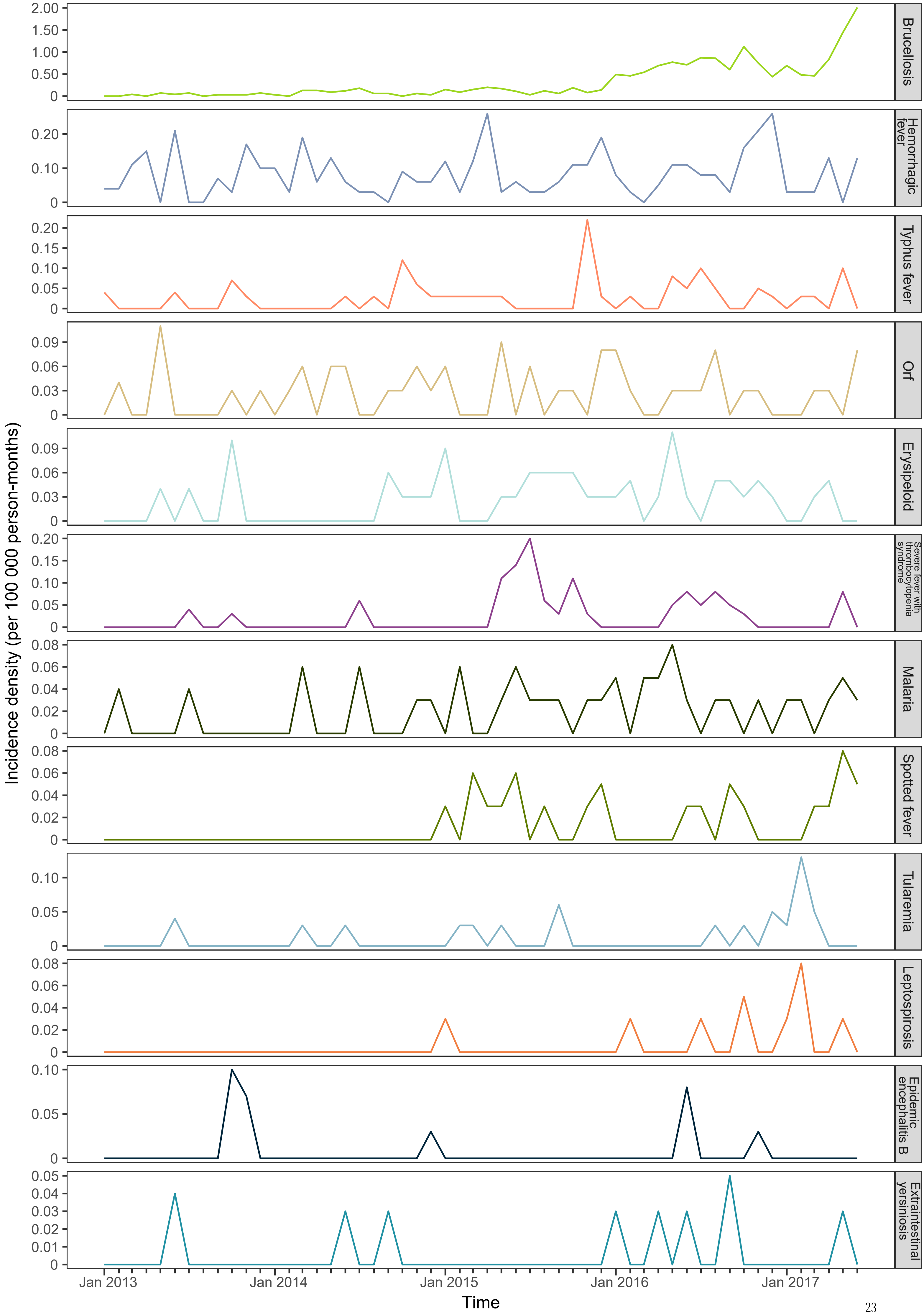
Brucellosis
Hemorrhagic fever
Typhus fever
Orf
Erysipeloid
Severe fever with thrombocytopenia syndrome
Malaria
Spotted fever
Tularemia
Leptospirosis
Epidemic encephalitis B
Extraintestinal yersiniosis
Incidence density (per 100 000 person-months)
Jan 2013
Jan 2014
Jan 2015
Jan 2016
Jan 2017
Time

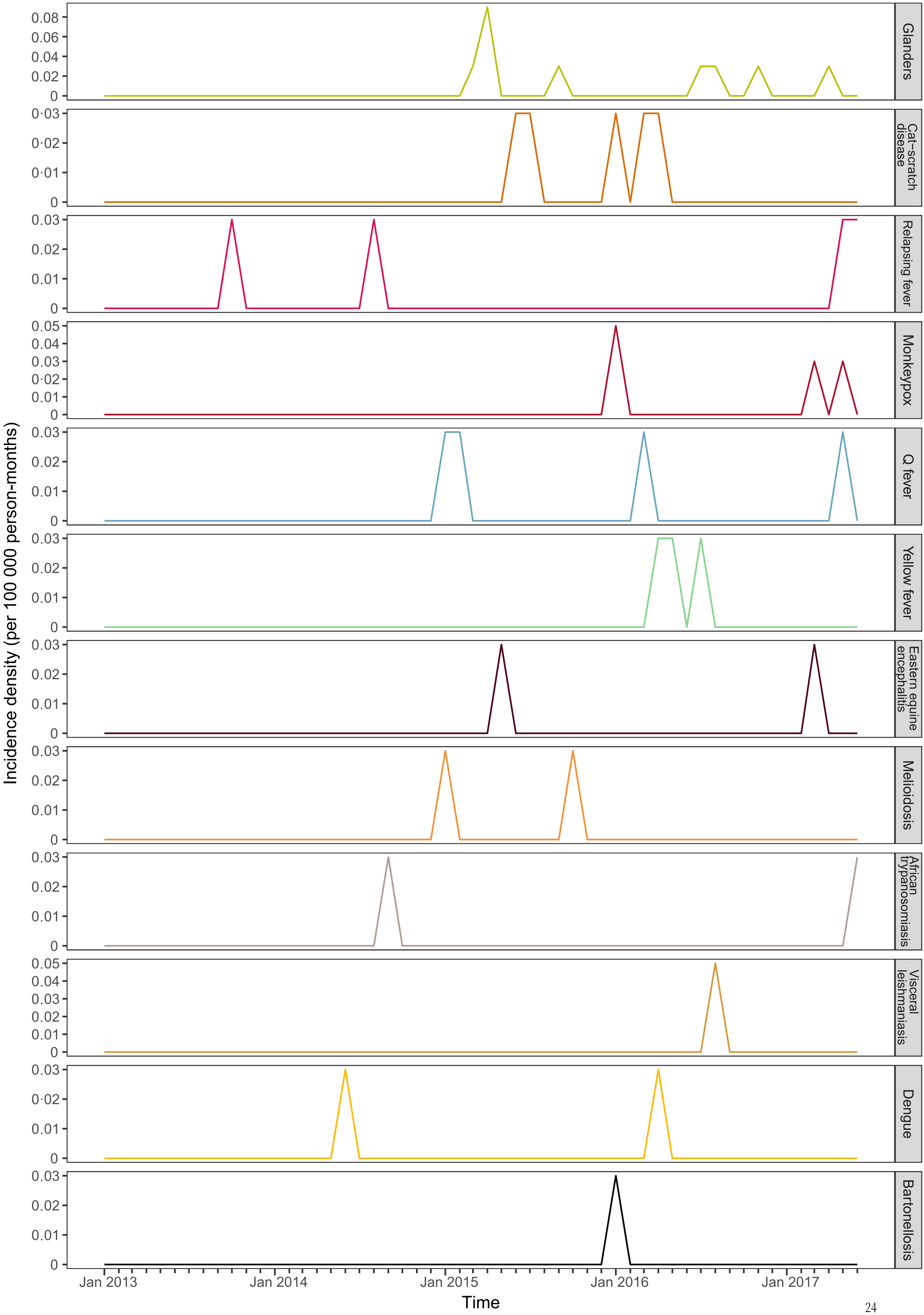
Glanders
Cat–scratch disease
Relapsing fever
Monkeypox
Q fever
Yellow fever
Eastern equine encephalitis
Melioidosis
African trypanosomiasis
Visceral leishmaniasis
Dengue
Bartonellosis
Incidence density (per 100 000 person-months)
Time
Jan 2013
Jan 2014
Jan 2015
Jan 2016
Jan 2017

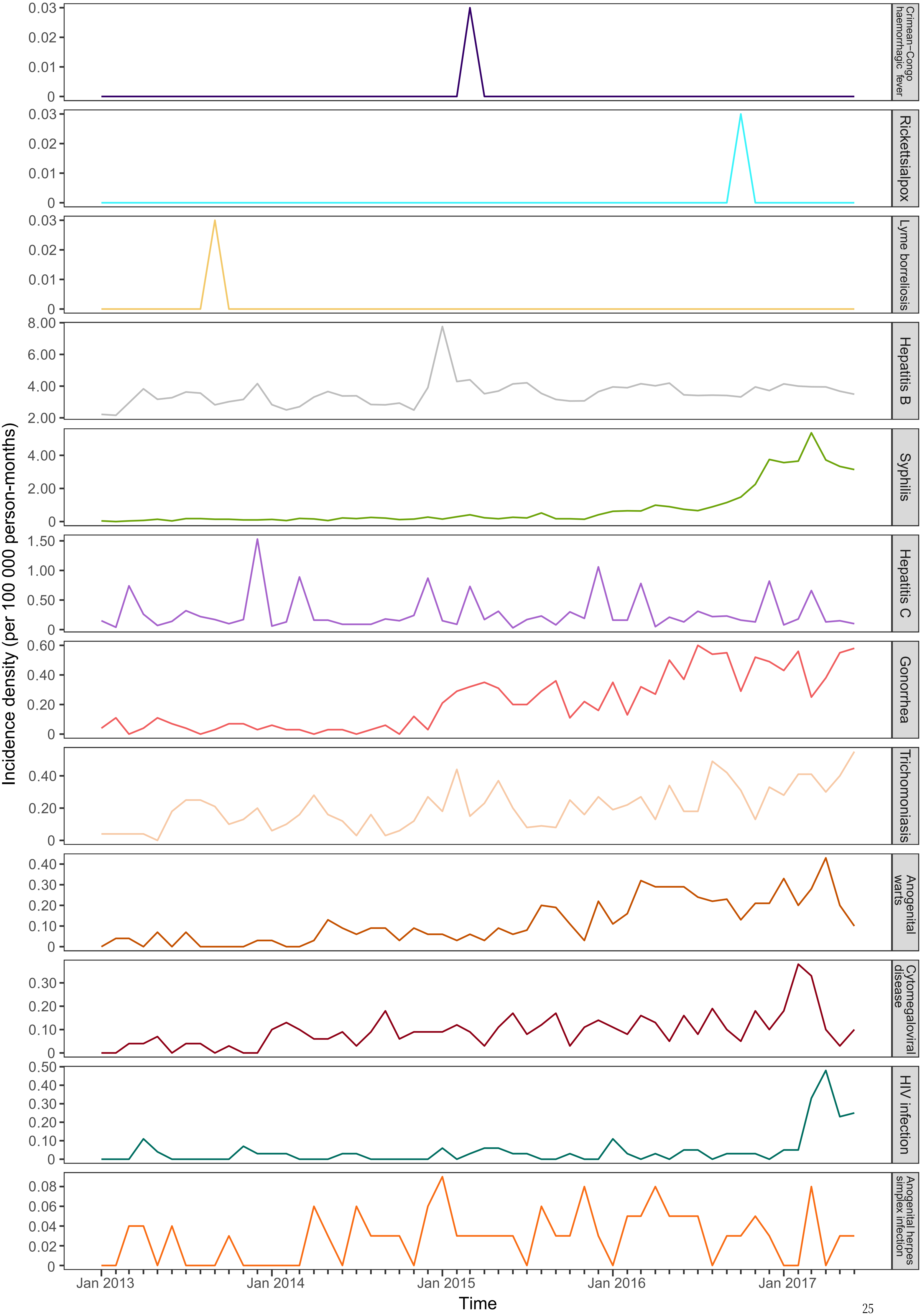
Crimean–Congo haemorrhagic fever
Rickettsialpox
Lyme borreliosis
Hepatitis B
Syphilis
Hepatitis C
Gonorrhea
Trichomoniasis
Anogenital warts
Cytomegaloviral disease
HIV infection
Anogenital herpes simplex infection
Incidence density (per 100 000 person-months)
Time
Jan 2013
Jan 2014
Jan 2015
Jan 2016
Jan 2017

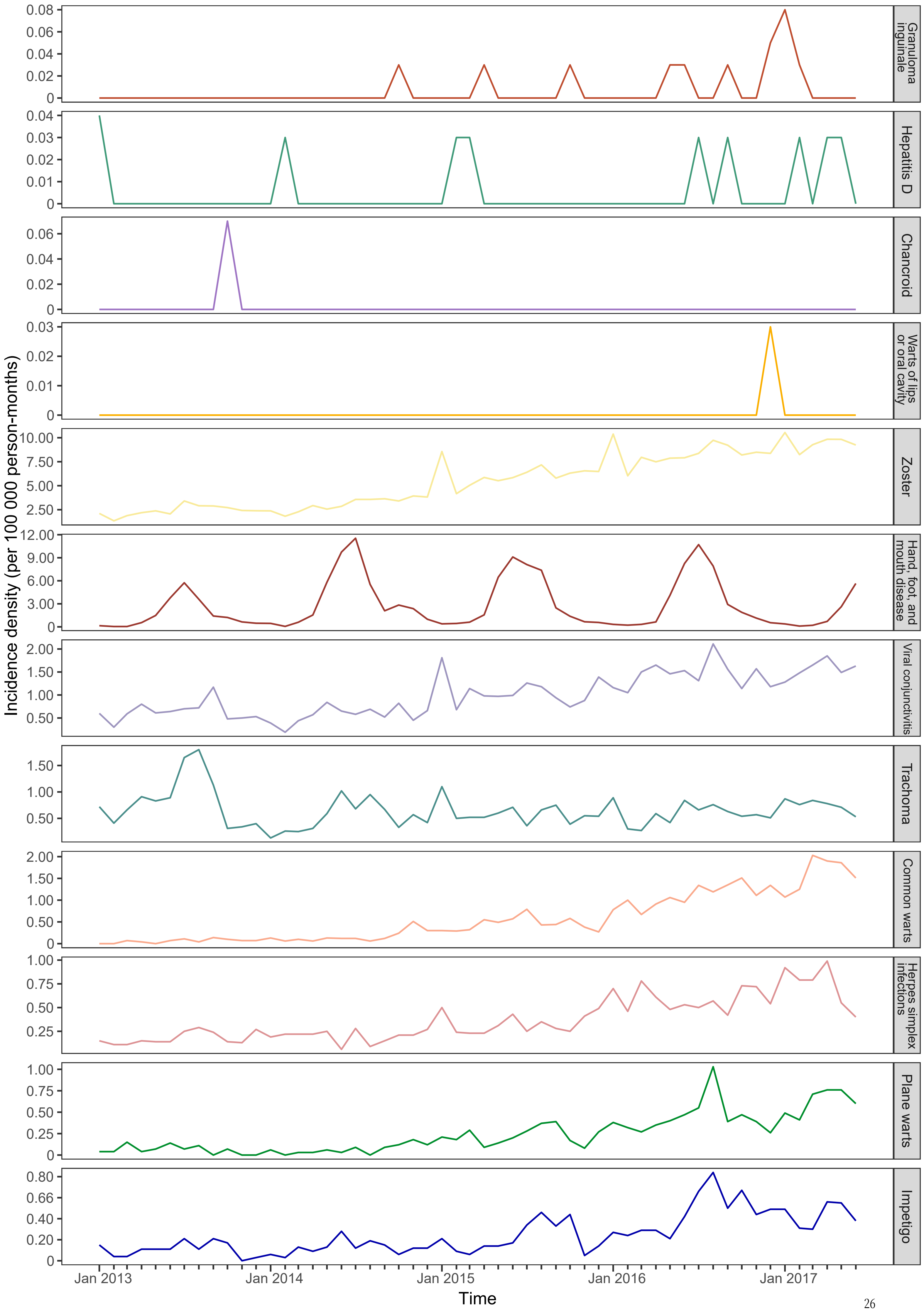
Granuloma inguinale
Hepatitis D
Chancroid
Warts of lips or oral cavity
Zoster
Hand, foot, and mouth disease
Viral conjunctivitis
Trachoma
Common warts
Herpes simplex infections
Plane warts
Impetigo
Incidence density (per 100 000 person-months)
Jan 2013
Jan 2014
Jan 2015
Jan 2016
Jan 2017
Time

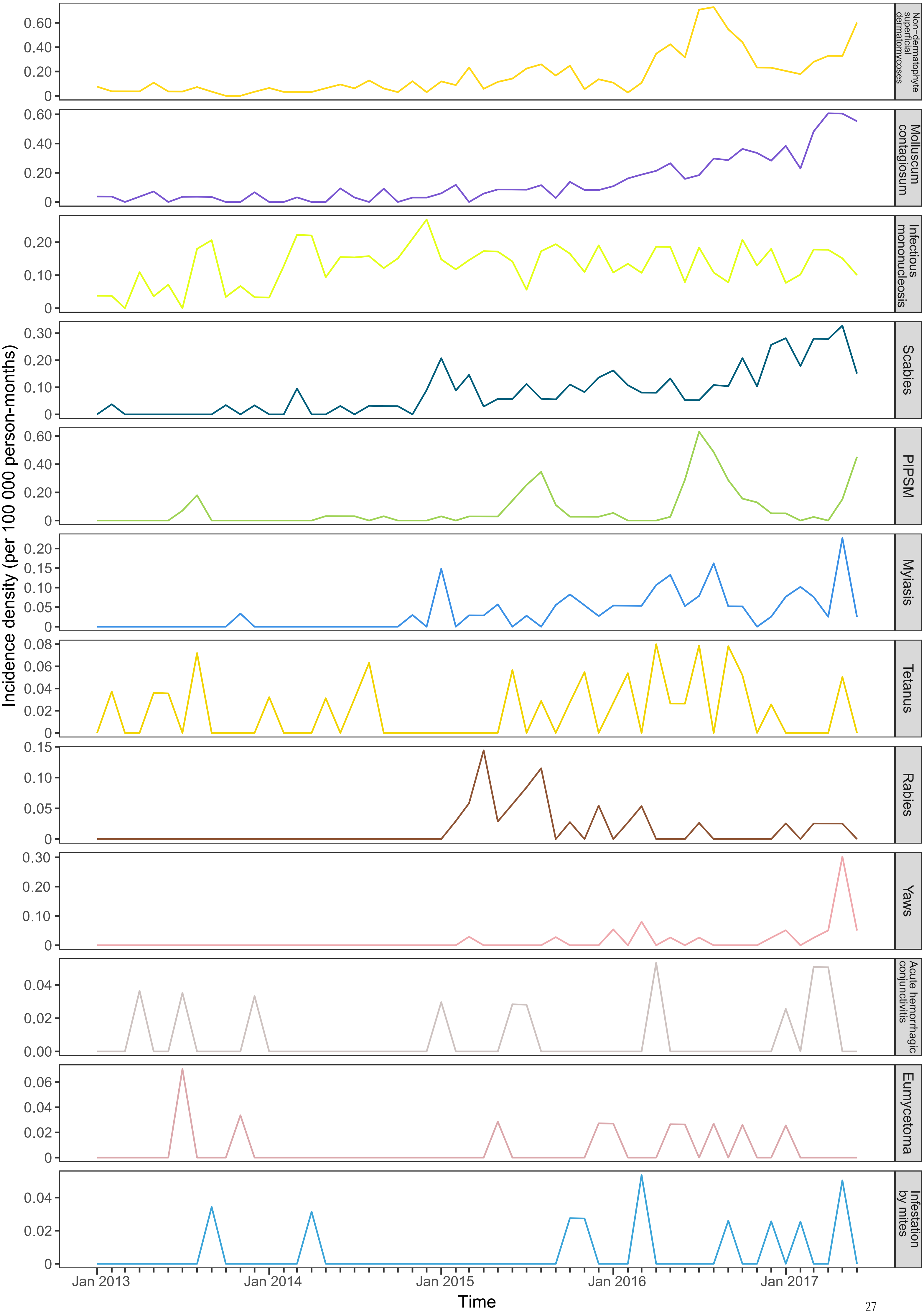
Non−dermatophyte superficial dermatomycoses
Molluscum contagiosum
Infectious mononucleosis
Scabies
PIPSM
Myiasis
Tetanus
Rabies
Yaws
Acute hemorrhagic conjunctivitis
Eumycetoma
Infestation by mites
Incidence density (per 100 000 person-months)
Jan 2013
Jan 2014
Jan 2015
Jan 2016
Jan 2017
Time

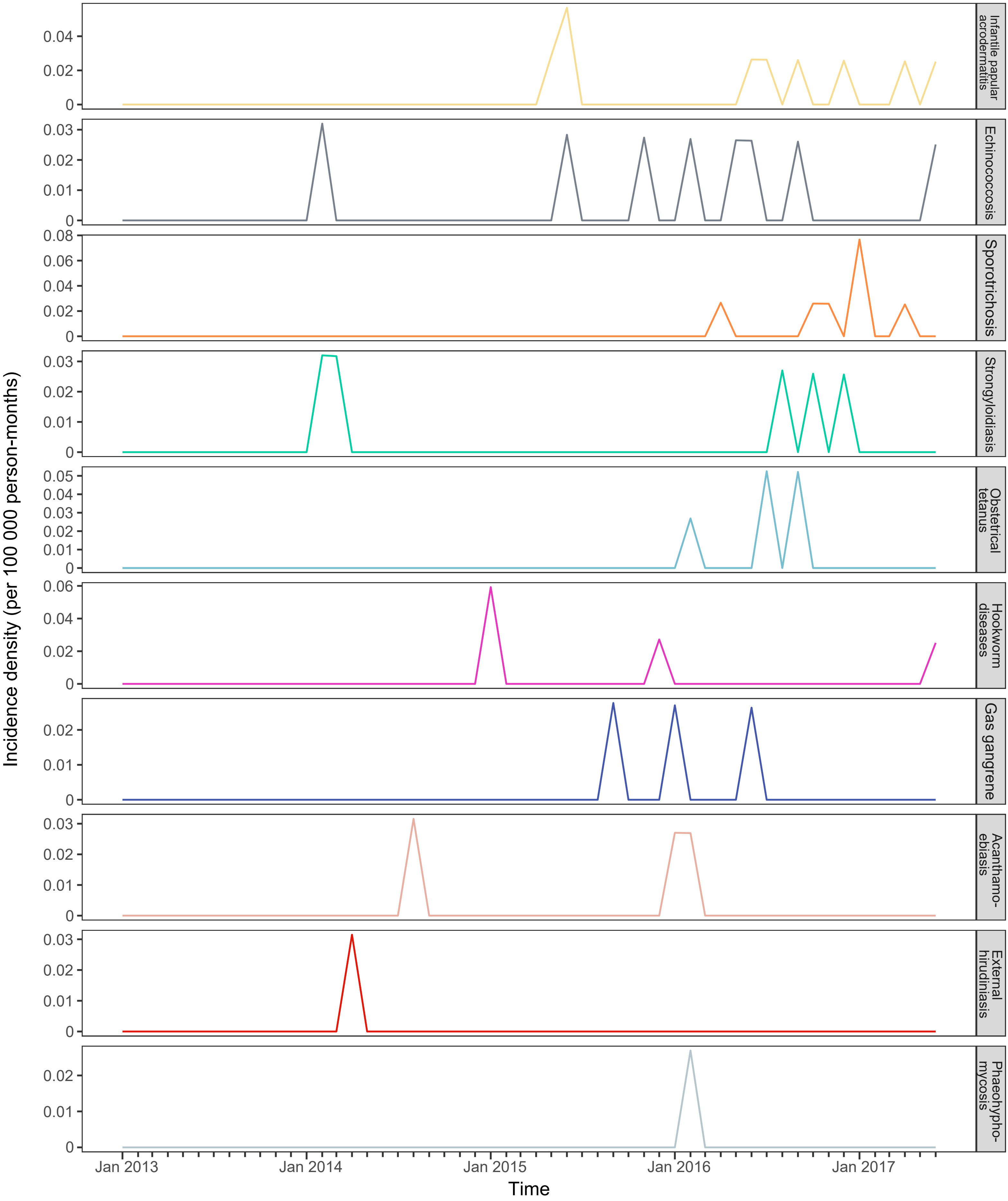


**Figure S3: Monthly incidence density of 130 infectious diseases, from January 2013 to June 2017.**

HIV=human immunodeficiency virus, PIPSM=picornavirus infections presenting in the skin or mucous membranes.

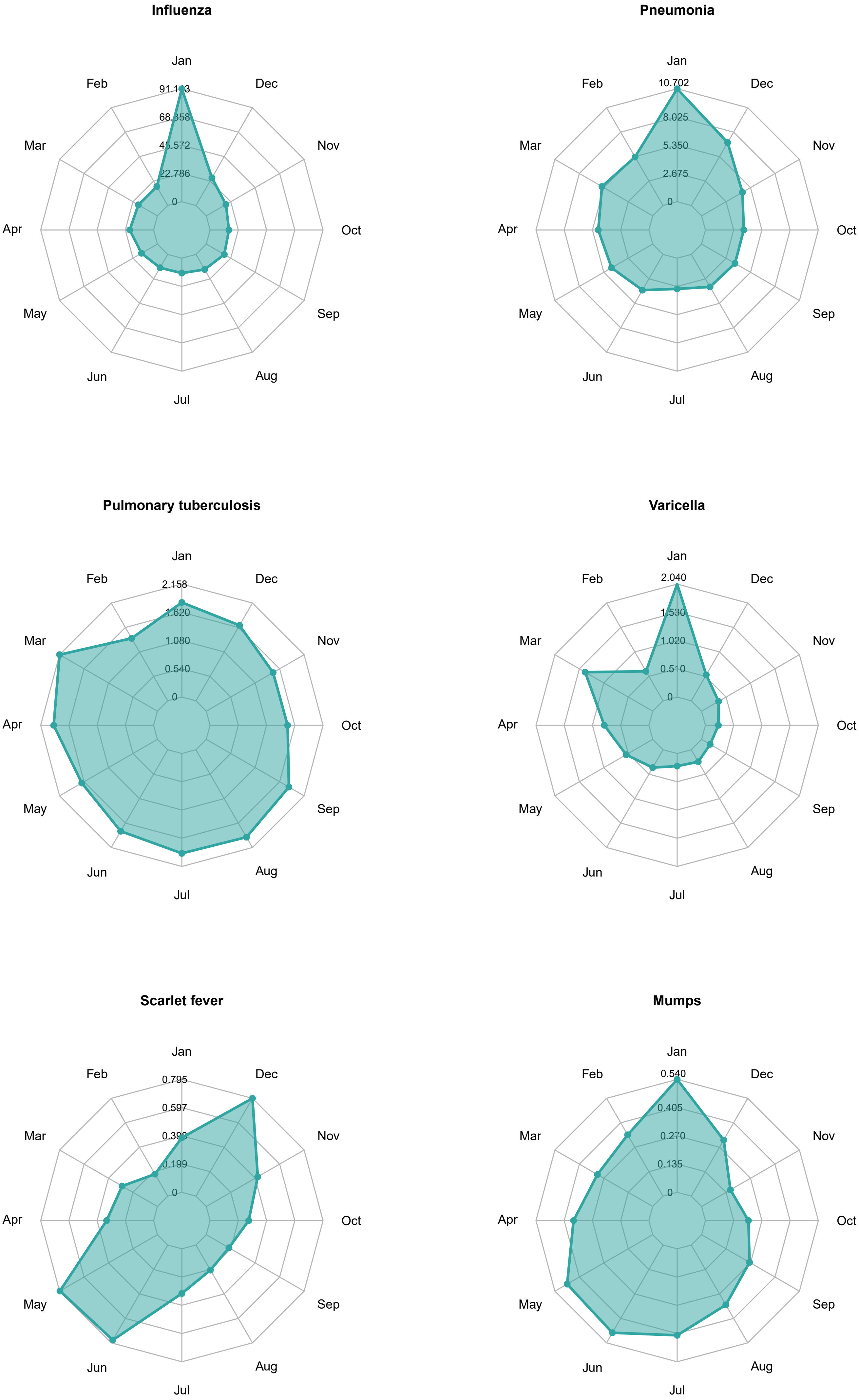
Influenza
Pneumonia
Pulmonary tuberculosis
Varicella
Scarlet fever
Mumps
Jan
Feb
Mar
Apr
May
Jun
Jul
Aug
Sep
Oct
Nov
Dec
22.786
10.702
8.025
5.350
2.675
2.158
1.080
0.540
2.040
1.020
0.795
0.597
0.199
0.540
0.405
0.270
0.135
0

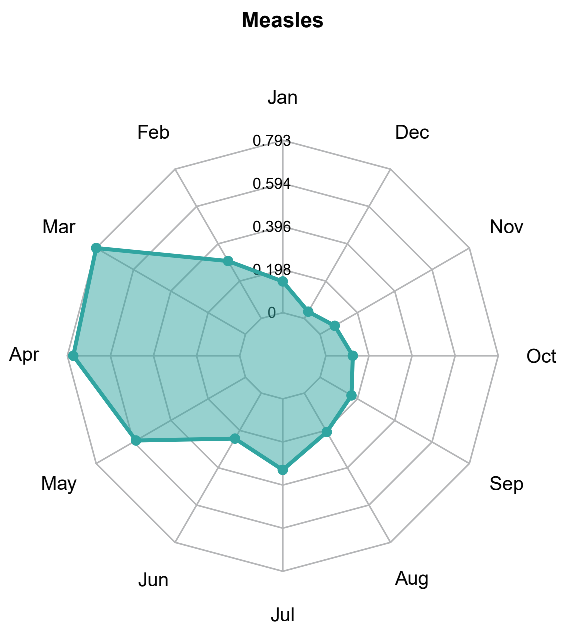
Measles
Jan
Dec
Nov
Oct
Sep
Aug
Jul
Jun
May
Apr
Mar
Feb
0.793
0.594
0.396
0.198
0

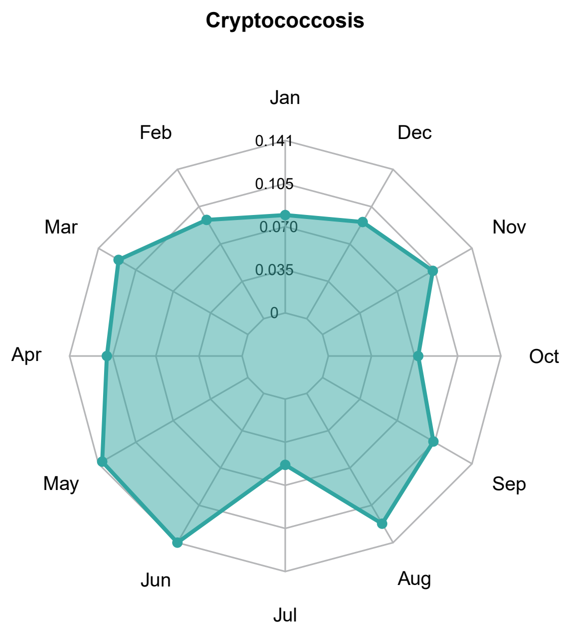
Cryptococcosis
Jan
Dec
Nov
Oct
Sep
Aug
Jul
Jun
May
Apr
Mar
Feb
0.141
0.105
0.070
0.035
0

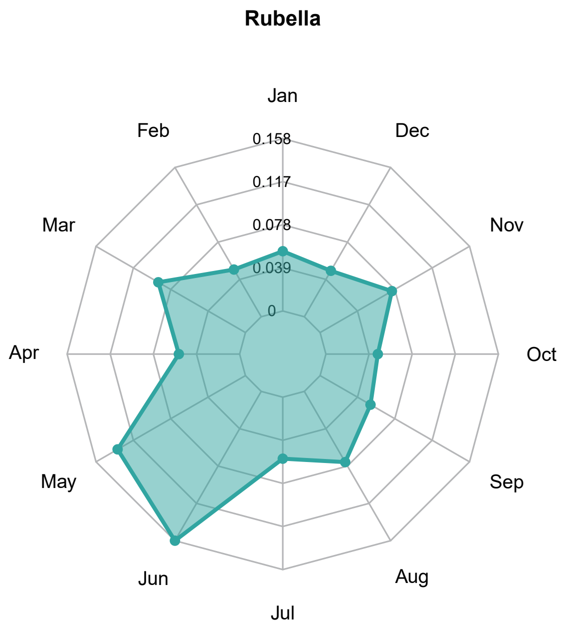
Rubella
Jan
Dec
Nov
Oct
Sep
Aug
Jul
Jun
May
Apr
Mar
Feb
0.158
0.117
0.078
0.039
0

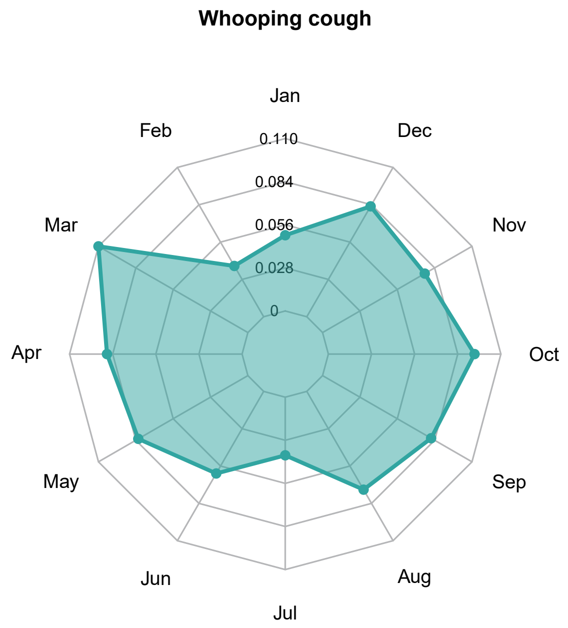
Whooping cough
Jan
Dec
Nov
Oct
Sep
Aug
Jul
Jun
May
Apr
Mar
Feb
0.110
0.084
0.056
0.028
0

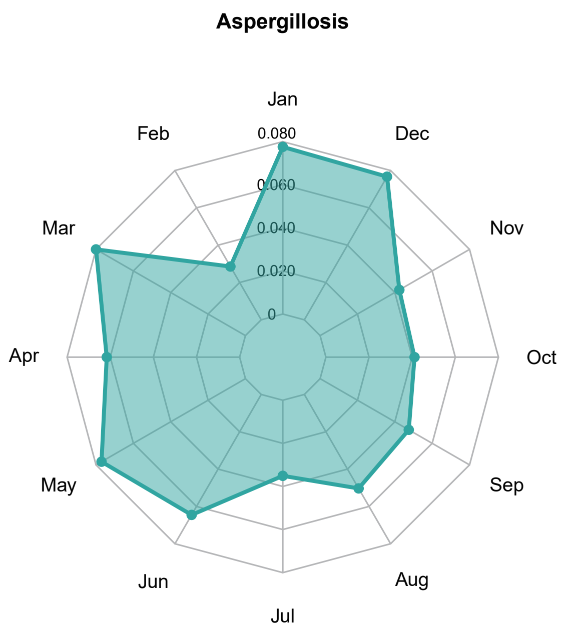
Aspergillosis
Jan
Dec
Nov
Oct
Sep
Aug
Jul
Jun
May
Apr
Mar
Feb
0.080
0.060
0.040
0.020
0

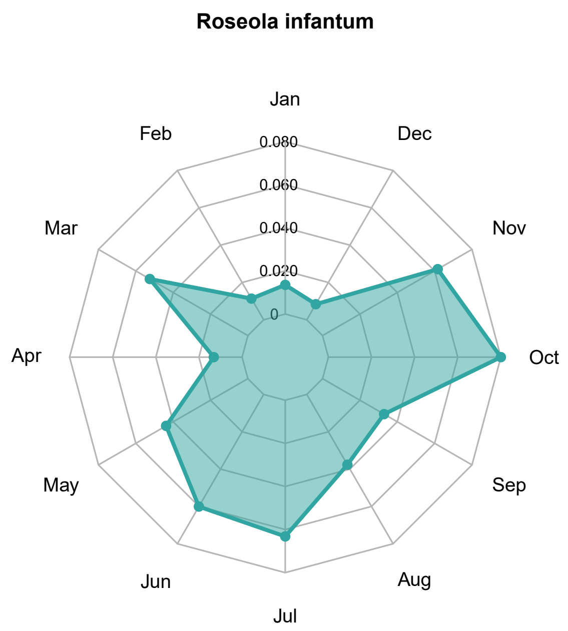
Roseola infantum
Jan
Dec
Nov
Oct
Sep
Aug
Jul
Jun
May
Apr
Mar
Feb
0.080
0.060
0.040
0.020
0

**Influenza A H1N1**

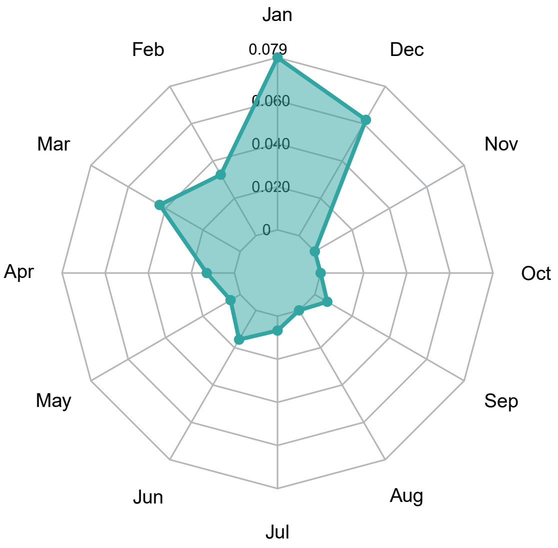


**Blastomycosis**

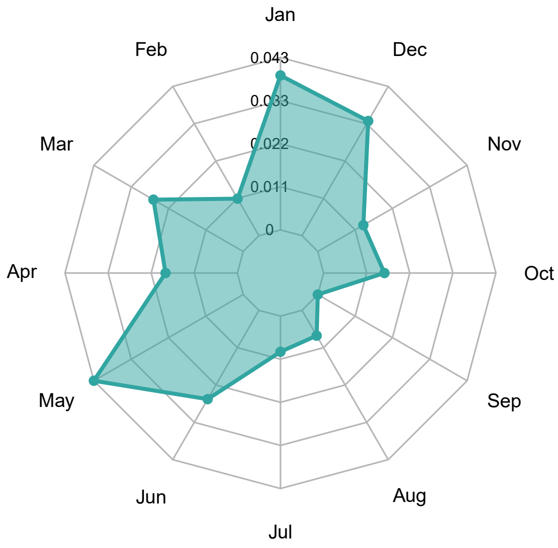


**Meningococcal disease**

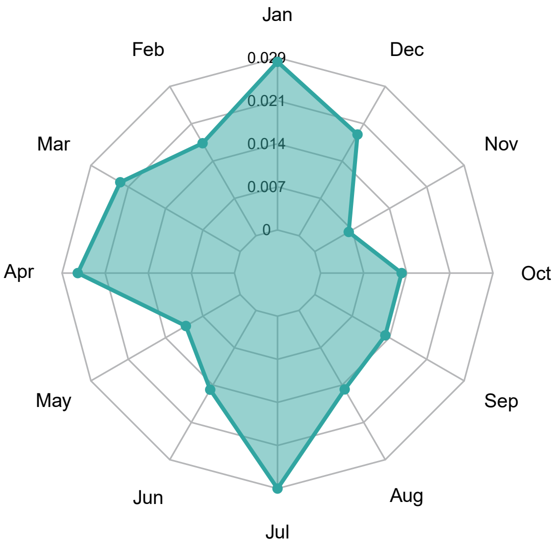


**Leprosy**

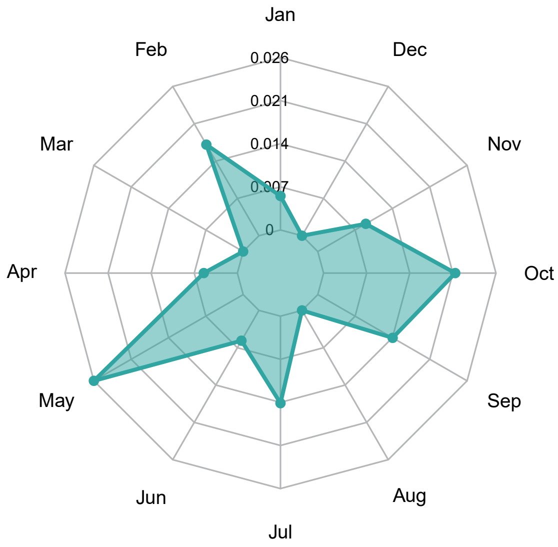


**Streptococcal pharyngitis**

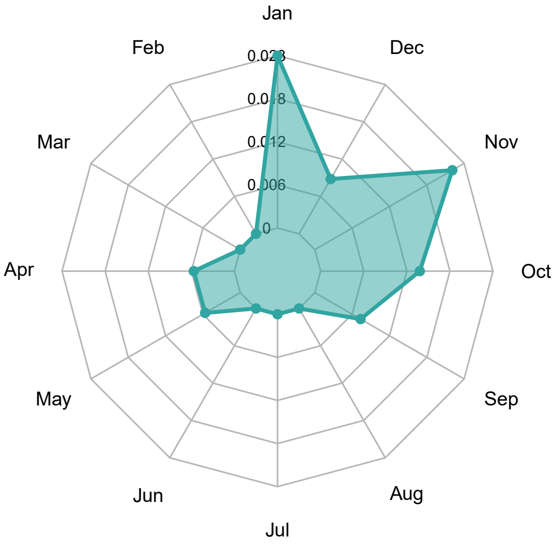


**Legionellosis**

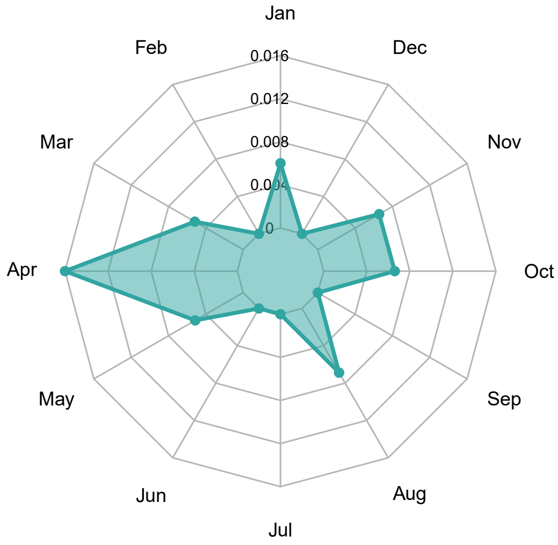

**Epidemic cerebrospinal meningitis**

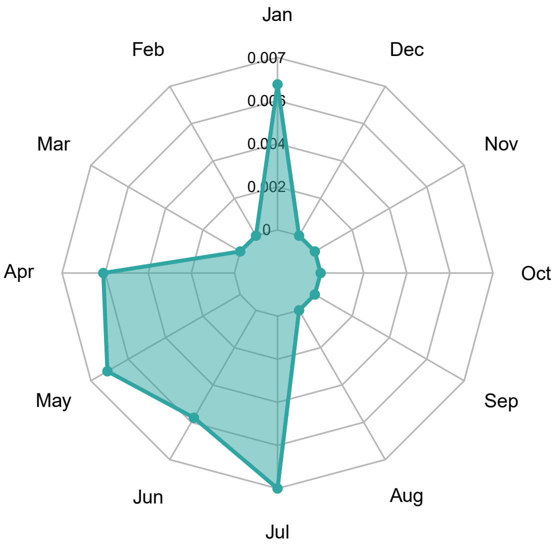


**Nocardiosis**

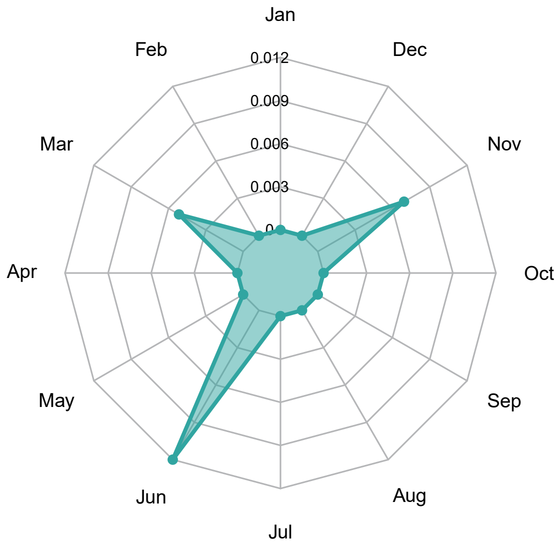


**Histoplasmosis**

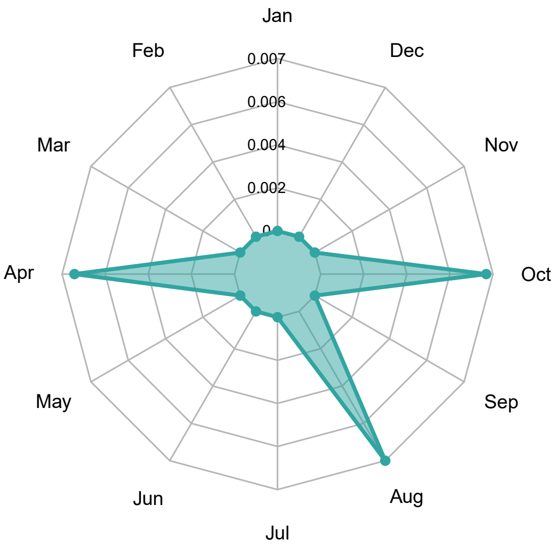


**Erythema infectiosum**

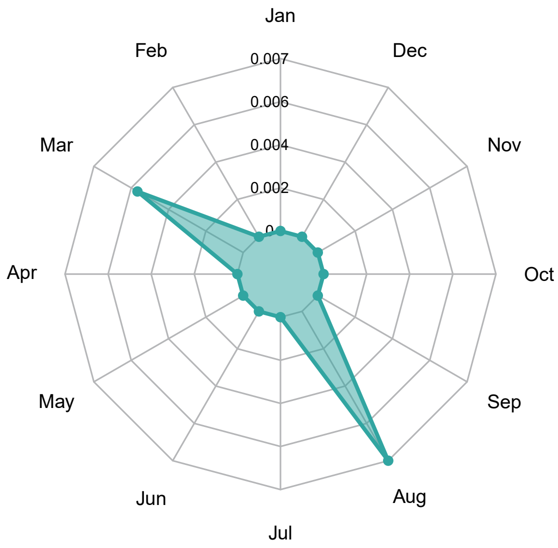


**Coccidioidomycosis**

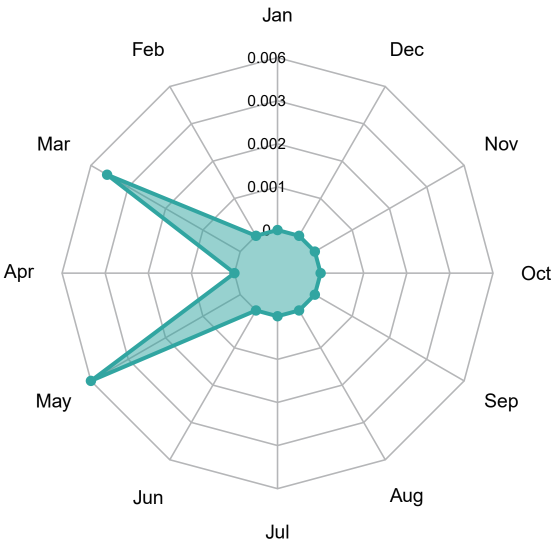


**Typhoid**

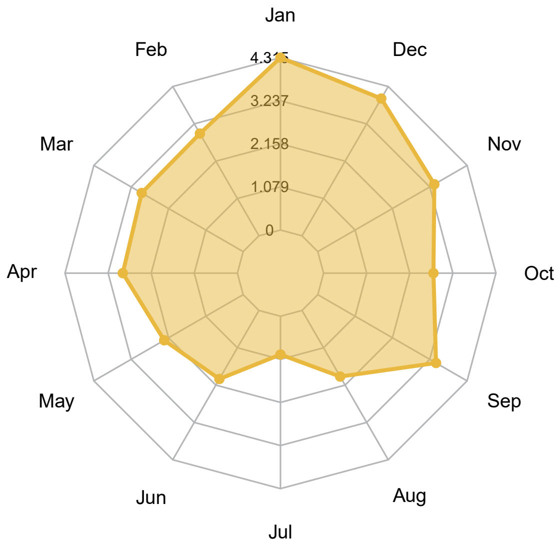

**Other infectious diarrhoea**

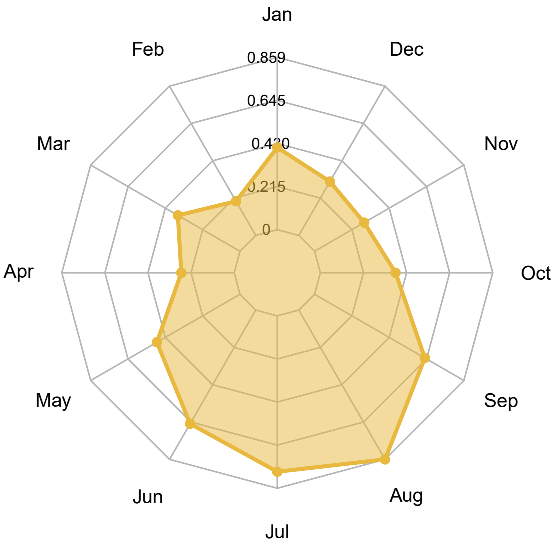


**Ascariasis**

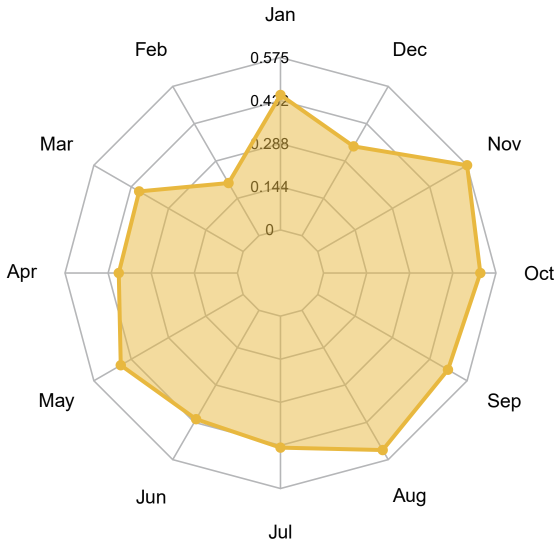


**Bacterial dysentery**

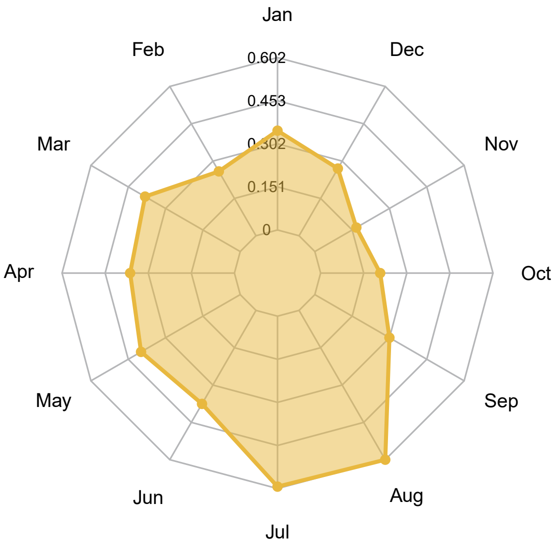


**Paratyphoid**

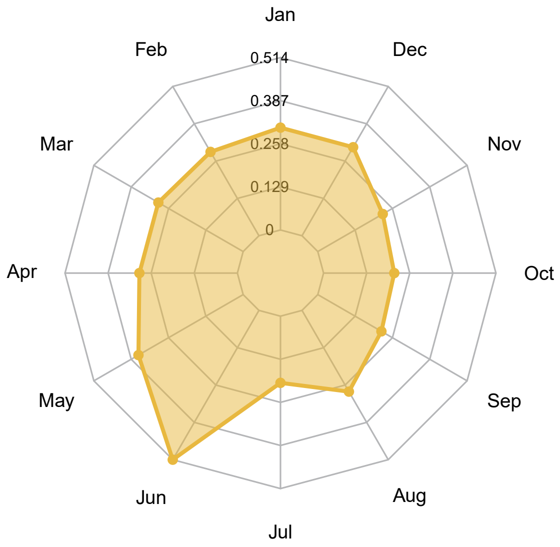


**Amoebic dysentery**

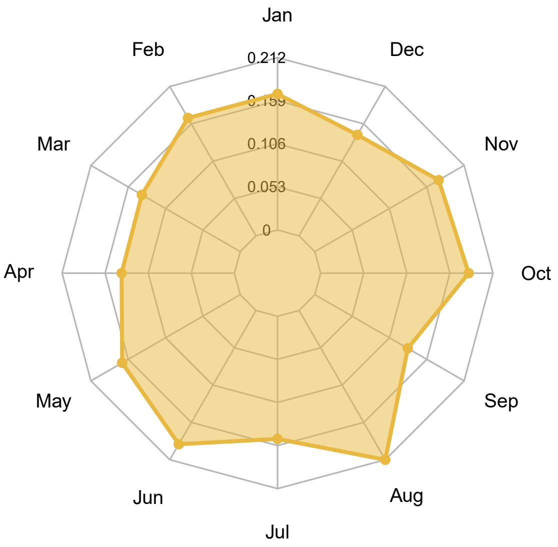


**Gastroenteritis due to Rotavirus**

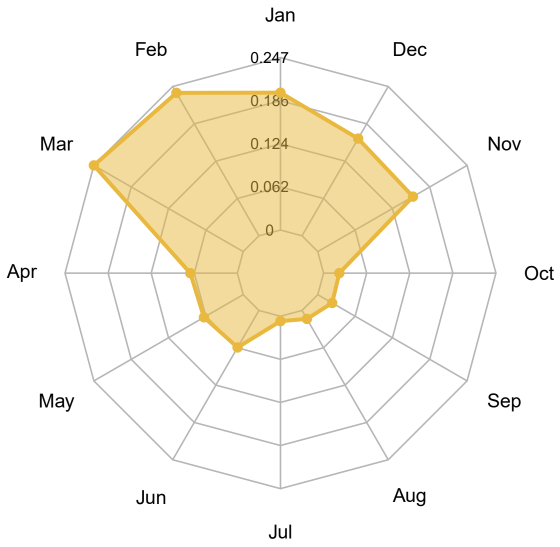

**Intestinal infections due to *Escherichia coli***

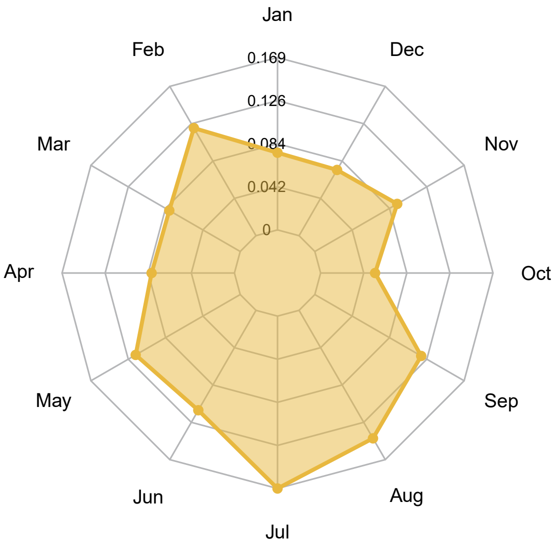


**Oesophagostomiasis**

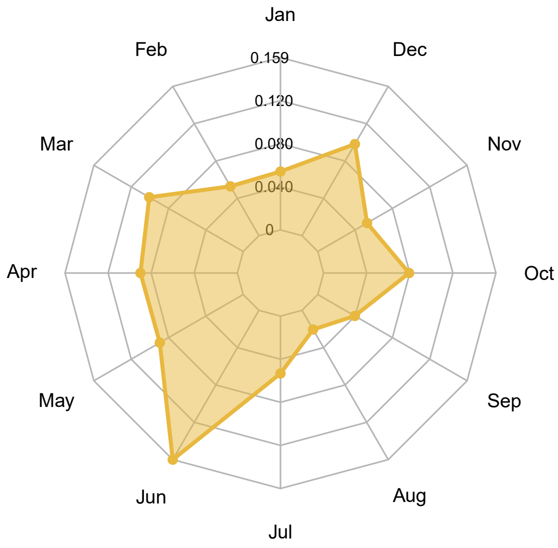


**Hepatitis A**

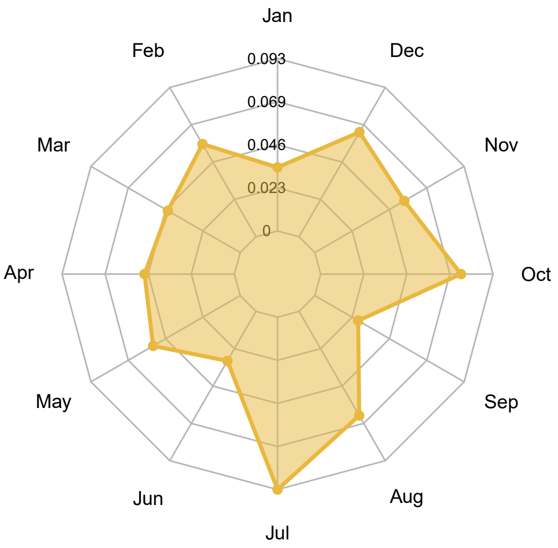


**Enterobiasis**

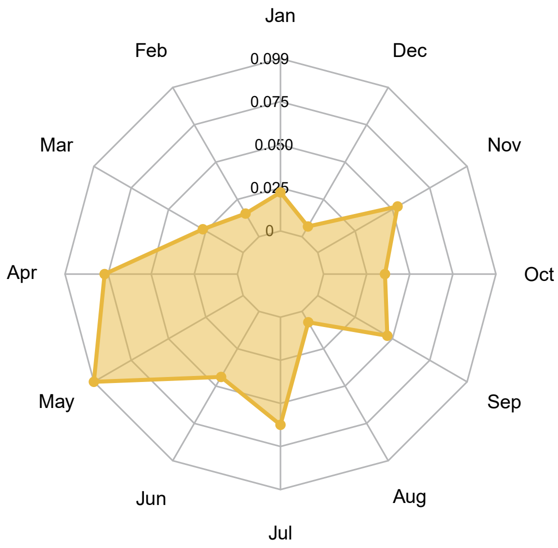


**Hepatitis E**

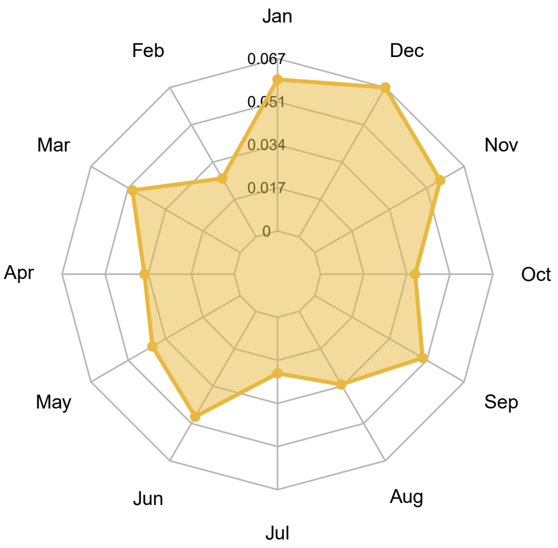


**Bacterial foodborne intoxications, unspecified**

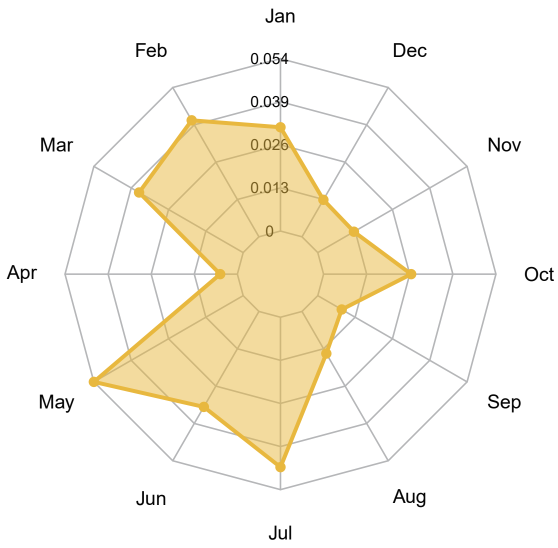

**Enteritis due to Norovirus**

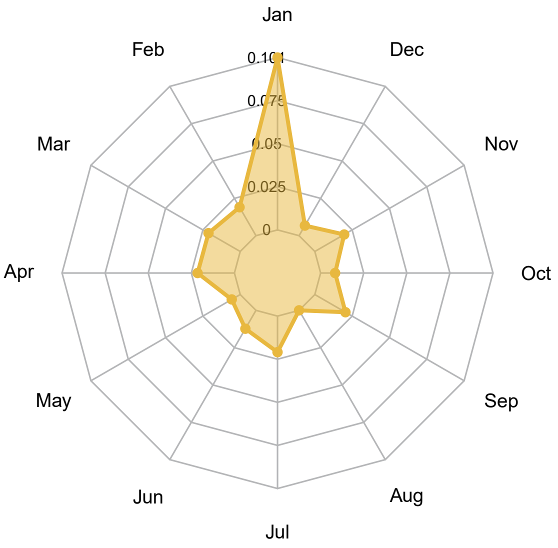


**Cysticercosis**

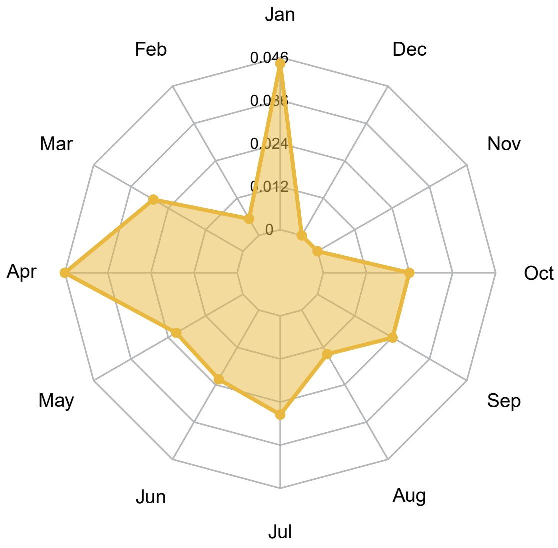


**Infections due to other *Salmonella***

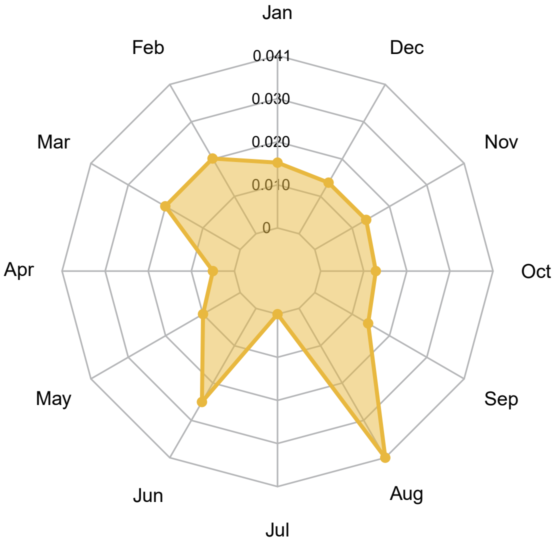


**Taeniasis**

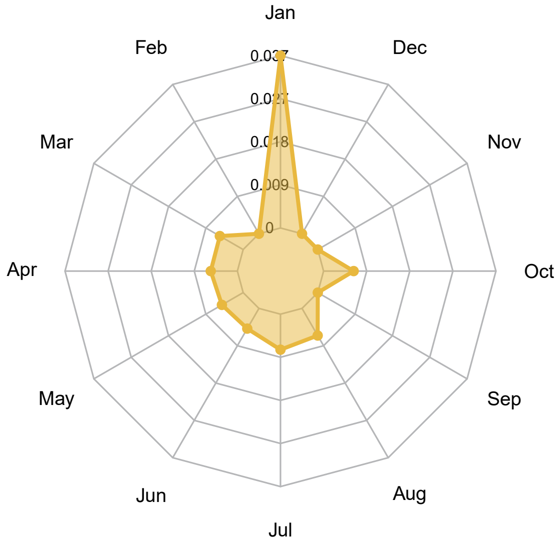


**Campylobacteriosis**

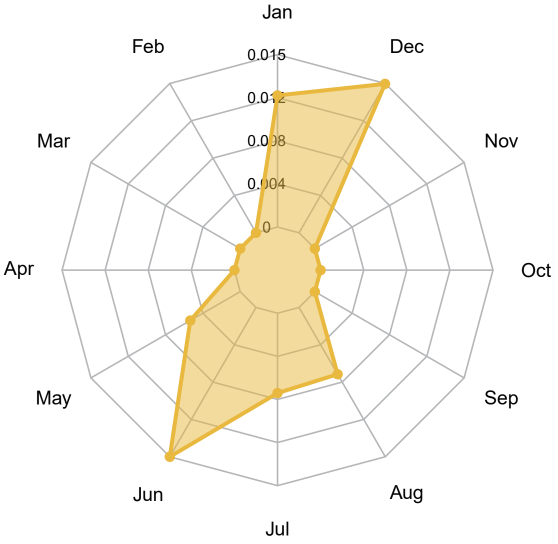


**Toxoplasmosis**

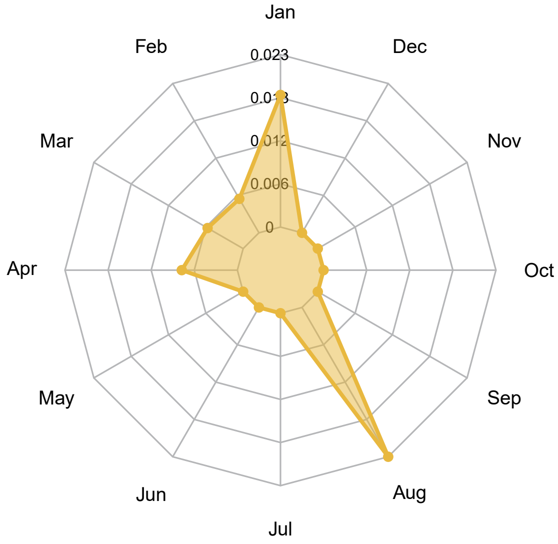

**Clonorchiasis**

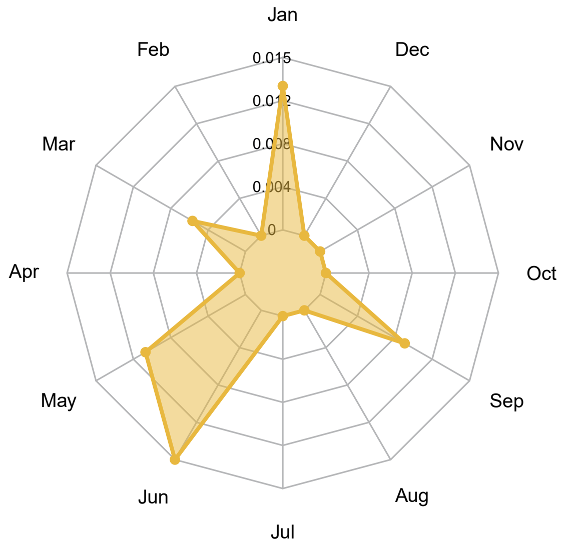


**Botulism**

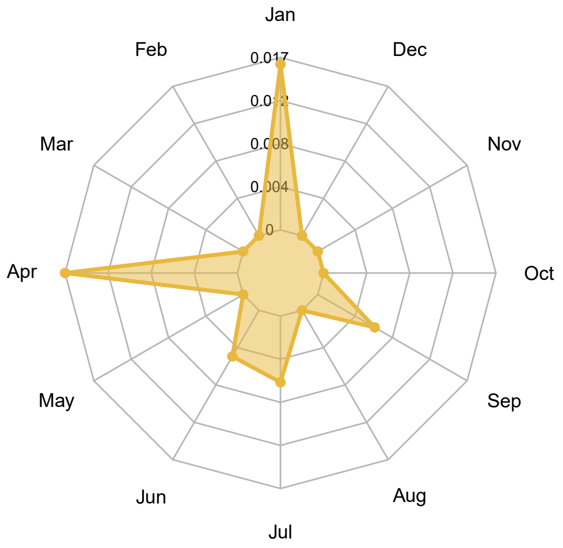


**Paragonimiasis**

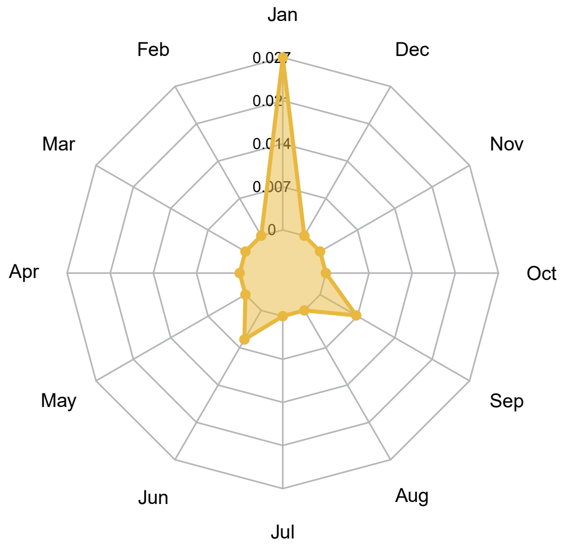


**Trichuriasis**

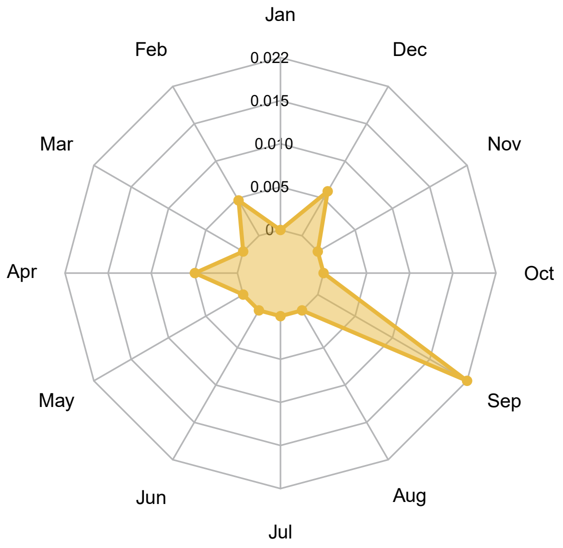


**Cryptosporidiosis**

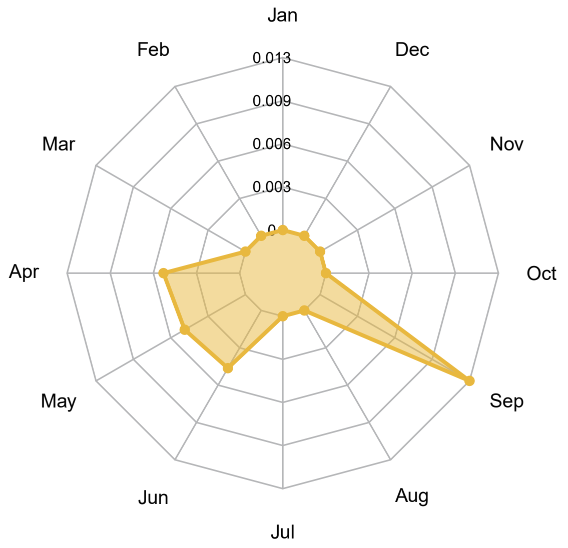


**Capillariasis**

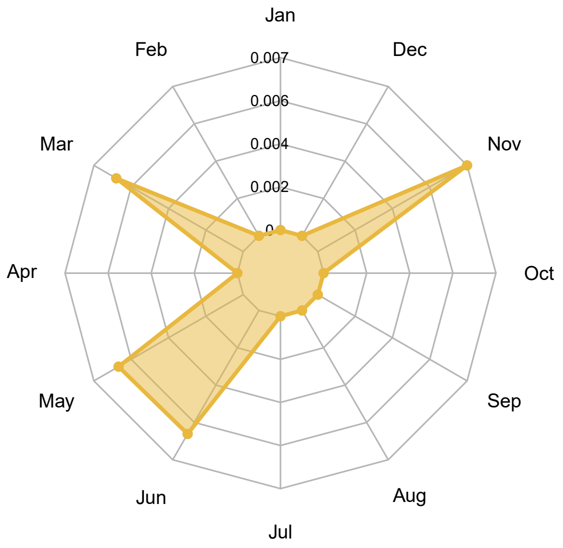

**Enteritis due to Adenovirus**

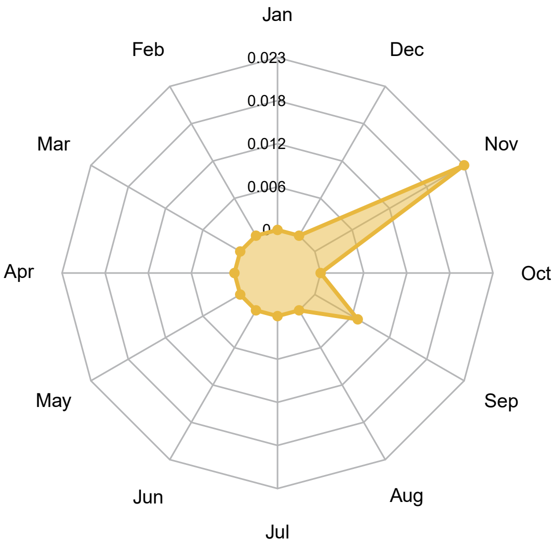


**Listeriosis**

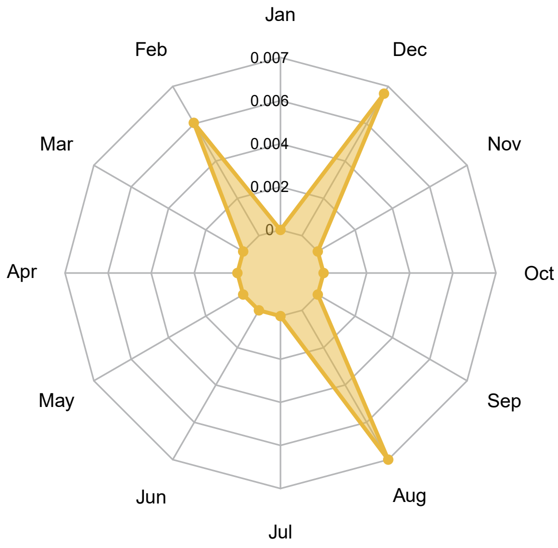


**Epidemic myalgia**

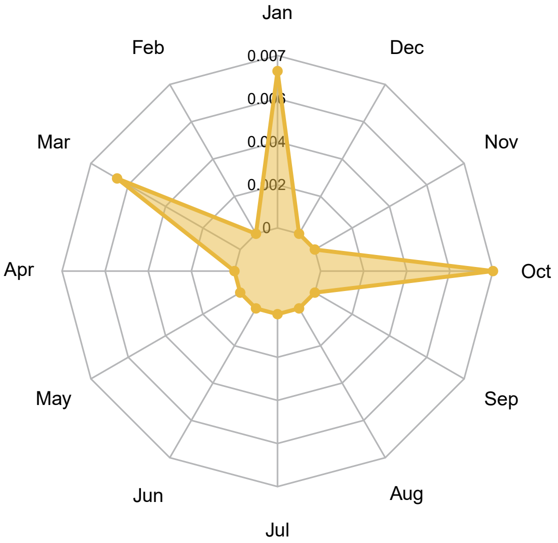


**Foodborne Bacillus cereus intoxication**

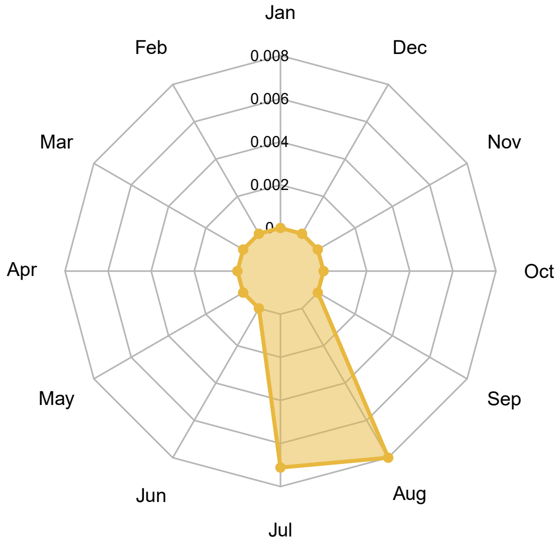


**Intestinal infections due to Yersinia enterocolitica**

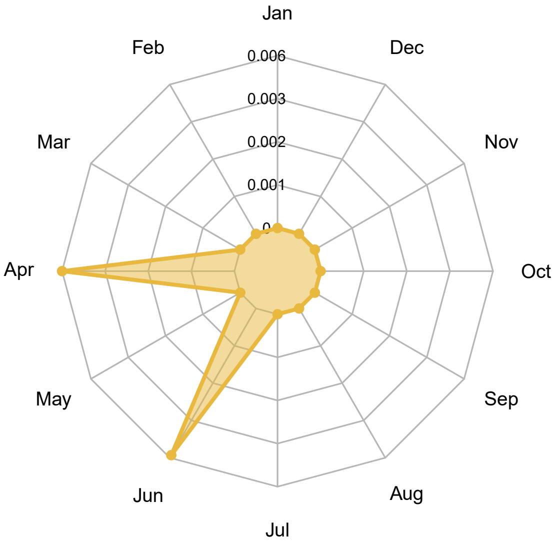


**Foodborne Clostridium perfringens intoxication**

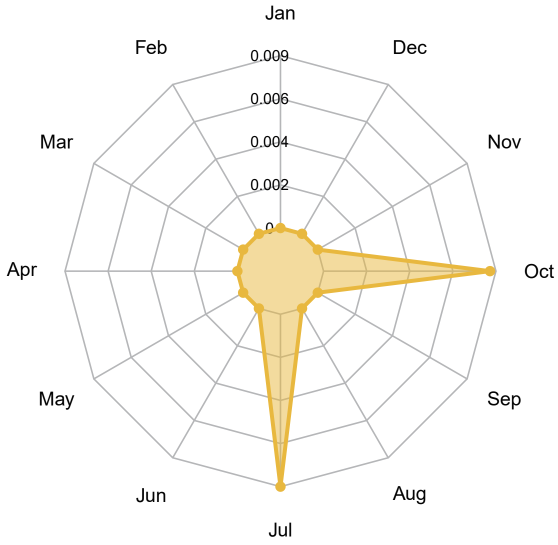

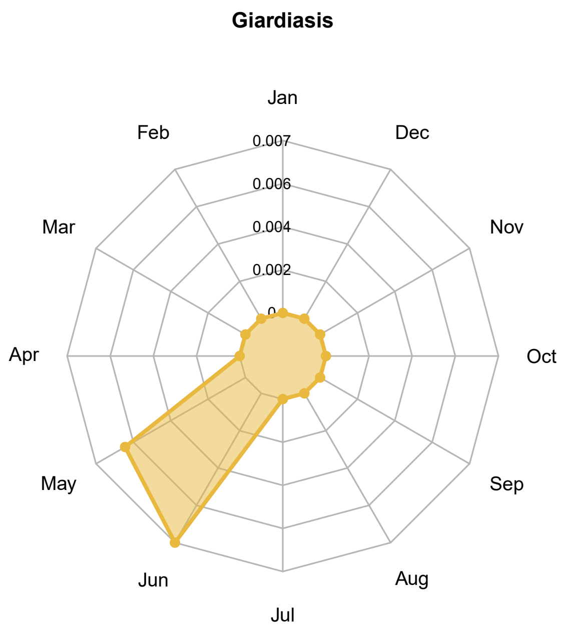
Giardiasis
Jan
Feb
Mar
Apr
May
Jun
Jul
Aug
Sep
Oct
Nov
Dec
0.007
0.006
0.004
0.002
0

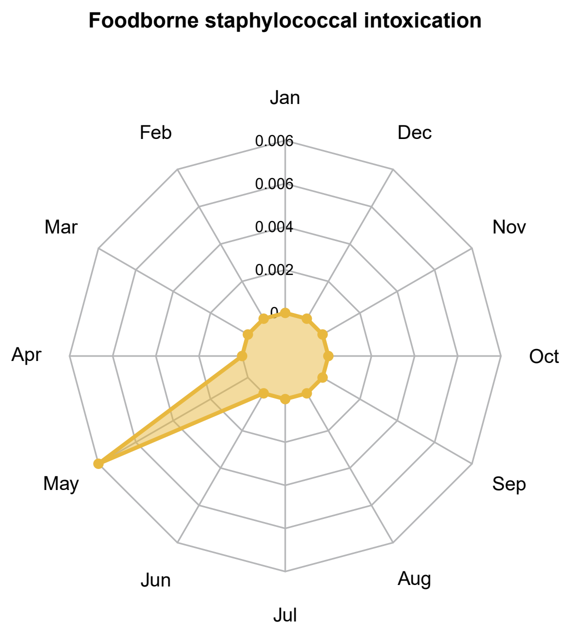
Foodborne staphylococcal intoxication
Jan
Feb
Mar
Apr
May
Jun
Jul
Aug
Sep
Oct
Nov
Dec
0.006
0.006
0.004
0.002
0

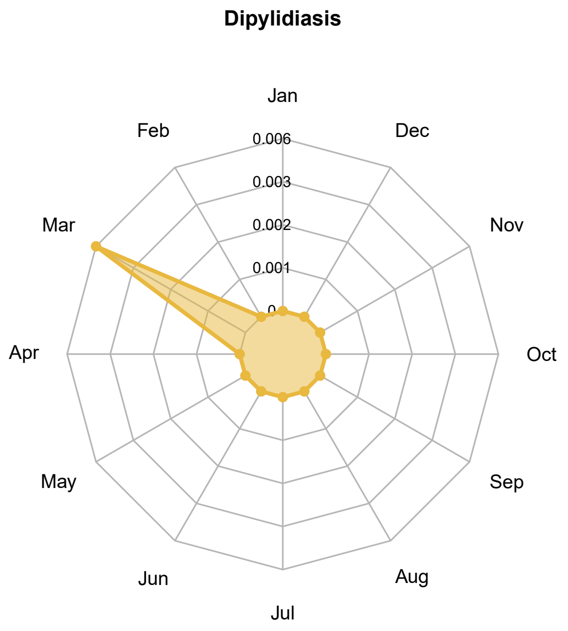
Dipylidiasis
Jan
Feb
Mar
Apr
May
Jun
Jul
Aug
Sep
Oct
Nov
Dec
0.006
0.003
0.002
0.001
0

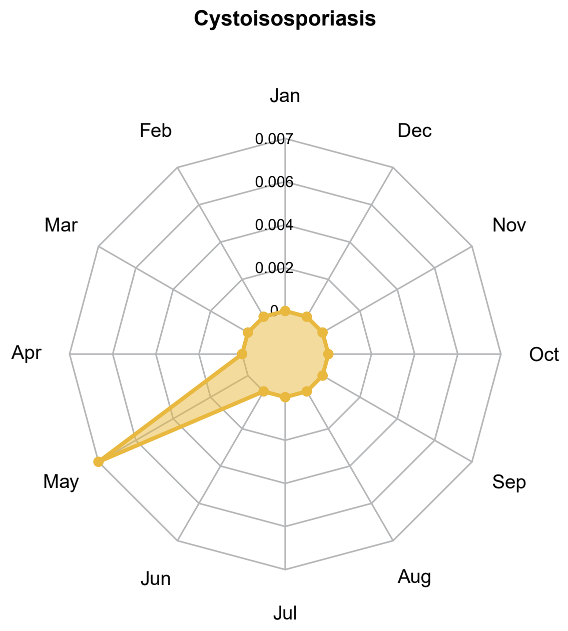
Cystoisosporiasis
Jan
Feb
Mar
Apr
May
Jun
Jul
Aug
Sep
Oct
Nov
Dec
0.007
0.006
0.004
0.002
0

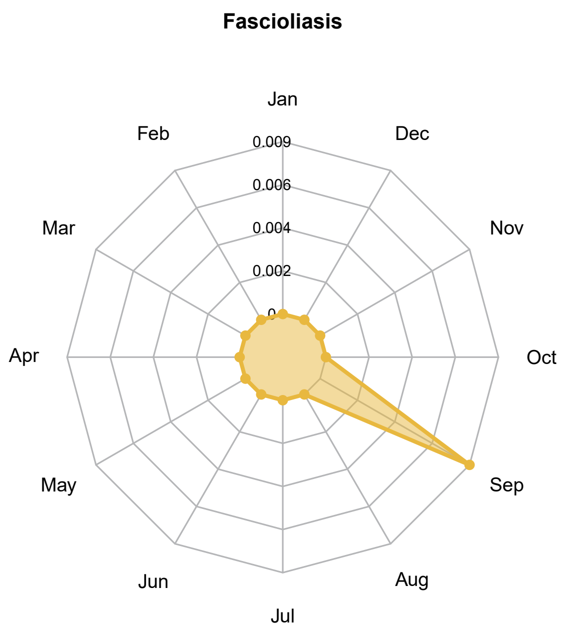
Fascioliasis
Jan
Feb
Mar
Apr
May
Jun
Jul
Aug
Sep
Oct
Nov
Dec
0.009
0.006
0.004
0.002
0

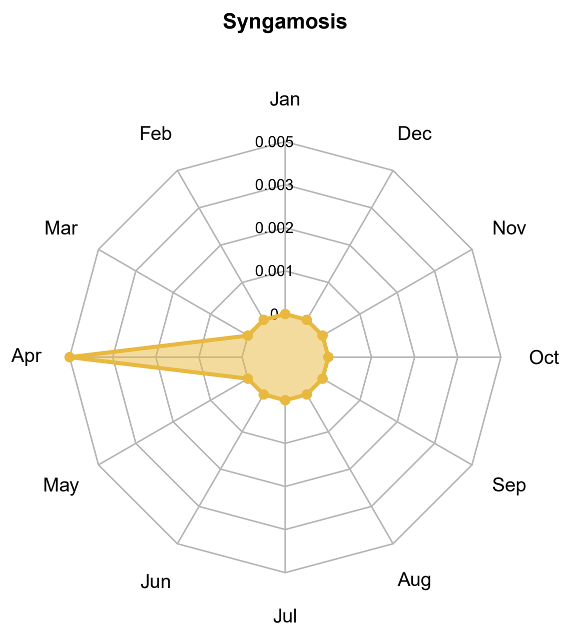
Syngamosis
Jan
Feb
Mar
Apr
May
Jun
Jul
Aug
Sep
Oct
Nov
Dec
0.005
0.003
0.002
0.001
0

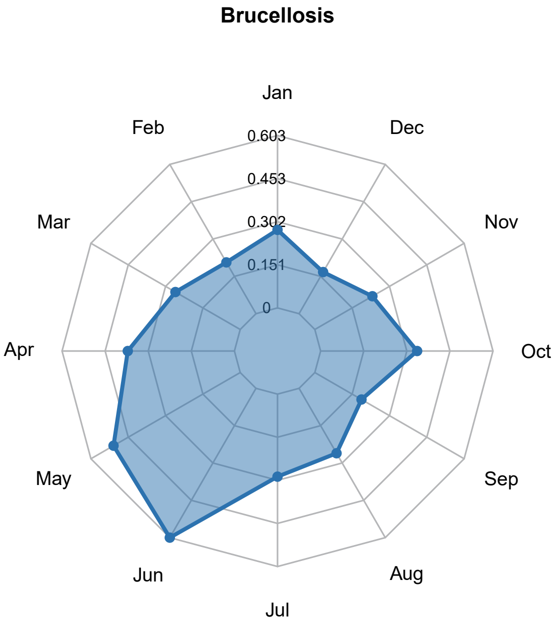
Brucellosis
Jan
Dec
Nov
Oct
Sep
Aug
Jul
Jun
May
Apr
Mar
Feb
0.603
0.453
0.302
0.151
0

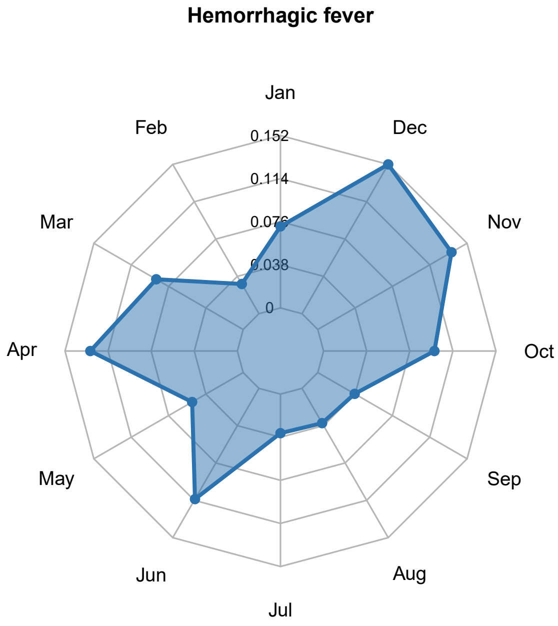
Hemorrhagic fever
Jan
Dec
Nov
Oct
Sep
Aug
Jul
Jun
May
Apr
Mar
Feb
0.152
0.114
0.038
0

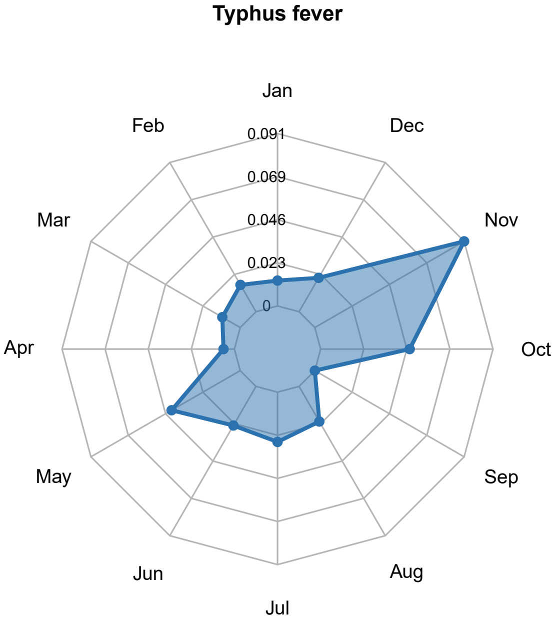
Typhus fever
Jan
Dec
Nov
Oct
Sep
Aug
Jul
Jun
May
Apr
Mar
Feb
0.091
0.069
0.046
0.023
0

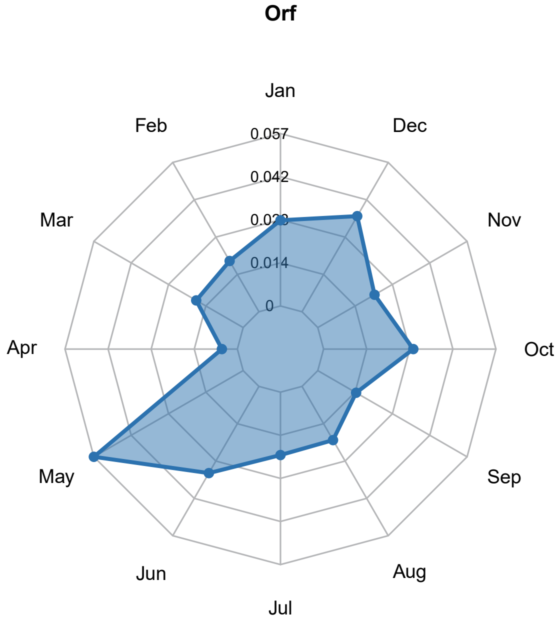
Orf
Jan
Dec
Nov
Oct
Sep
Aug
Jul
Jun
May
Apr
Mar
Feb
0.057
0.042
0.014
0

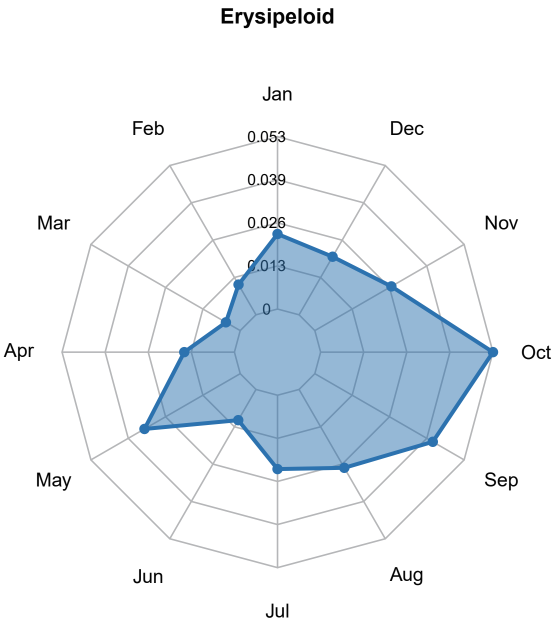
Erysipeloid
Jan
Dec
Nov
Oct
Sep
Aug
Jul
Jun
May
Apr
Mar
Feb
0.053
0.039
0.026
0.013
0

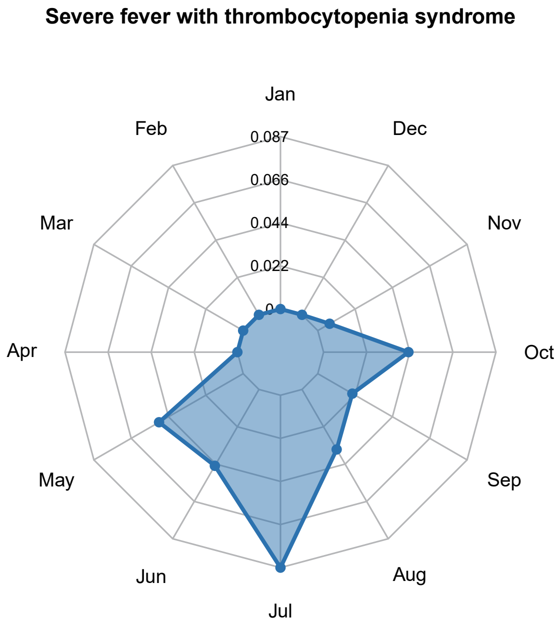
Severe fever with thrombocytopenia syndrome
Jan
Dec
Nov
Oct
Sep
Aug
Jul
Jun
May
Apr
Mar
Feb
0.087
0.066
0.044
0.022

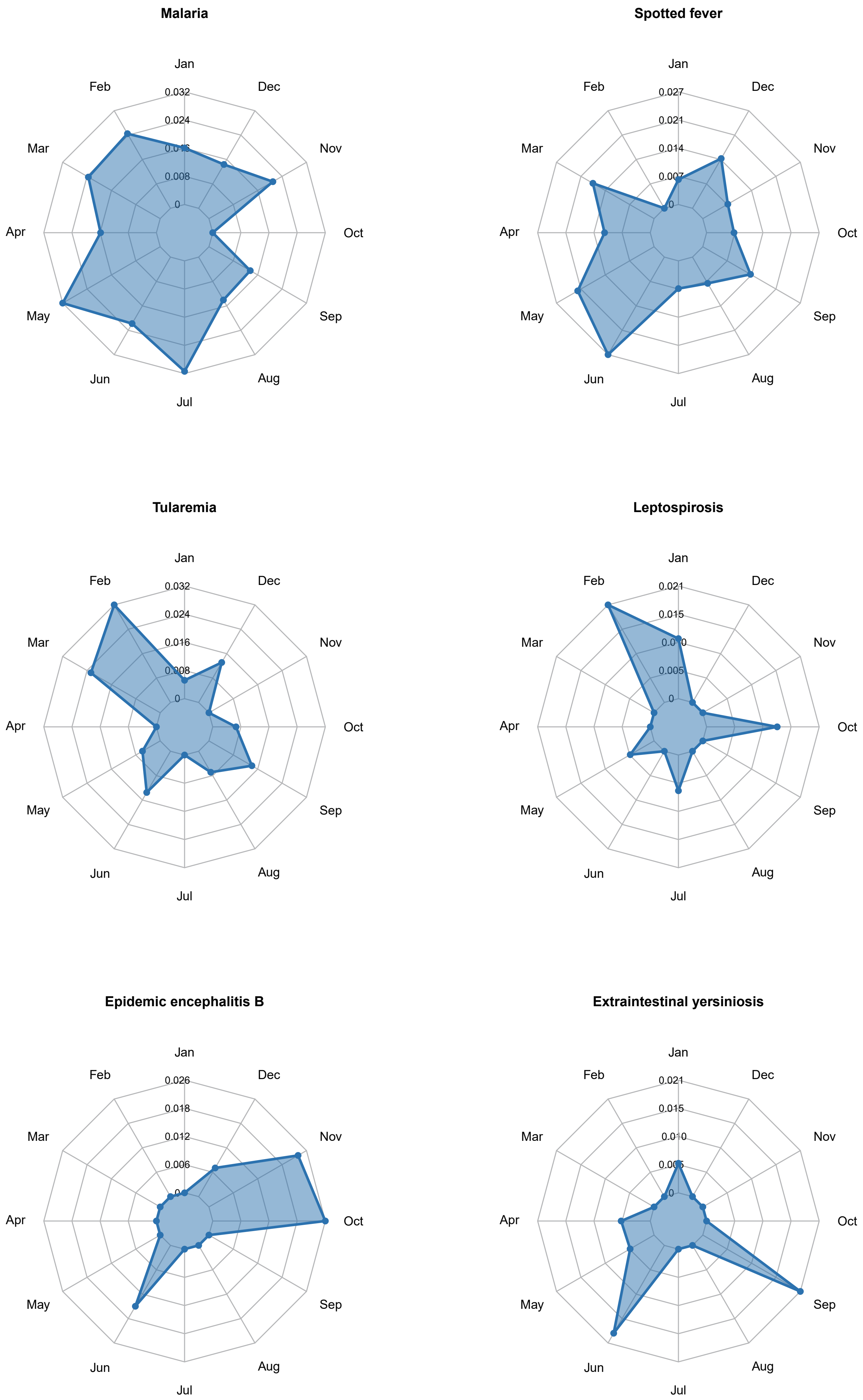
Malaria
Jan
Feb
Mar
Apr
May
Jun
Jul
Aug
Sep
Oct
Nov
Dec
0.032
0.024
0.008
0
Spotted fever
Jan
Feb
Mar
Apr
May
Jun
Jul
Aug
Sep
Oct
Nov
Dec
0.027
0.021
0.014
0.007
0
Tularemia
Jan
Feb
Mar
Apr
May
Jun
Jul
Aug
Sep
Oct
Nov
Dec
0.032
0.024
0.016
0.008
0
Leptospirosis
Jan
Feb
Mar
Apr
May
Jun
Jul
Aug
Sep
Oct
Nov
Dec
0.021
0.015
0.010
0.005
0
Epidemic encephalitis B
Jan
Feb
Mar
Apr
May
Jun
Jul
Aug
Sep
Oct
Nov
Dec
0.026
0.018
0.012
0.006
0
Extraintestinal yersiniosis
Jan
Feb
Mar
Apr
May
Jun
Jul
Aug
Sep
Oct
Nov
Dec
0.021
0.015
0.010
0.005
0

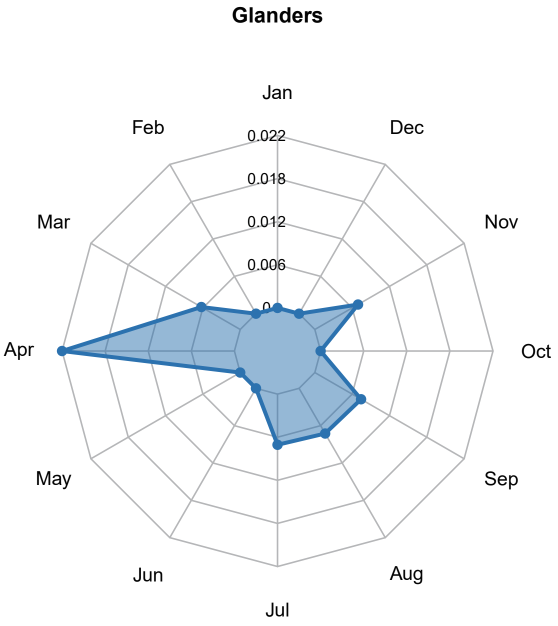
Glanders
Jan
Feb
Dec
Mar
Nov
Apr
Oct
May
Sep
Jun
Aug
Jul
0.022
0.018
0.012
0.006

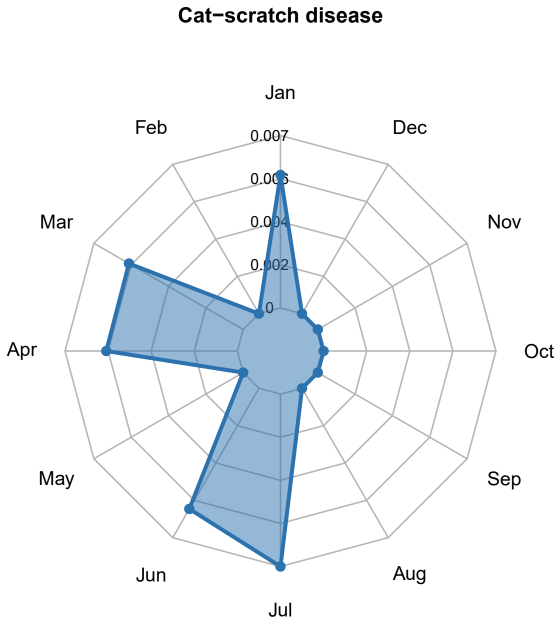
Cat−scratch disease
Jan
Feb
Dec
Mar
Nov
Apr
Oct
May
Sep
Jun
Aug
Jul
0.007
0.002
0

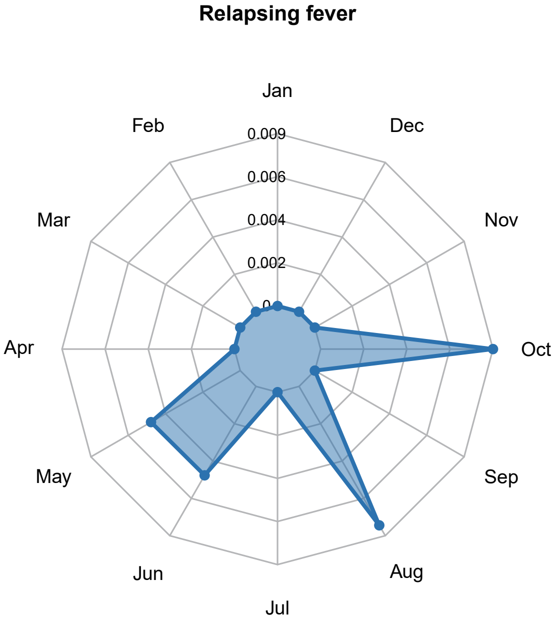
Relapsing fever
Jan
Feb
Dec
Mar
Nov
Apr
Oct
May
Sep
Jun
Aug
Jul
0.009
0.006
0.004
0.002

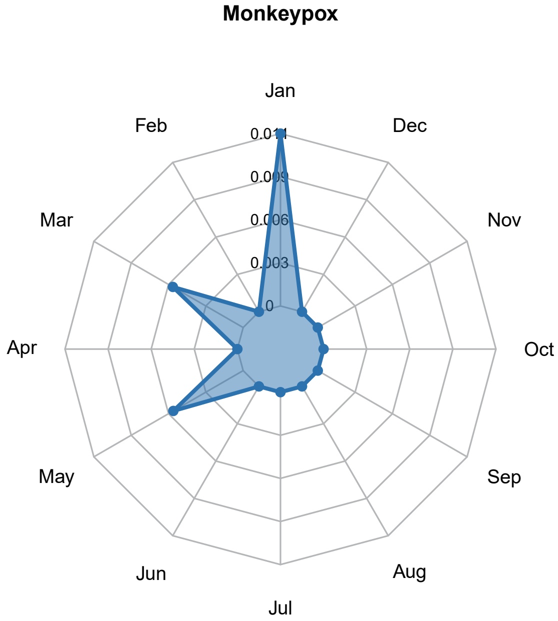
Monkeypox
Jan
Feb
Dec
Mar
Nov
Apr
Oct
May
Sep
Jun
Aug
Jul
0.003
0

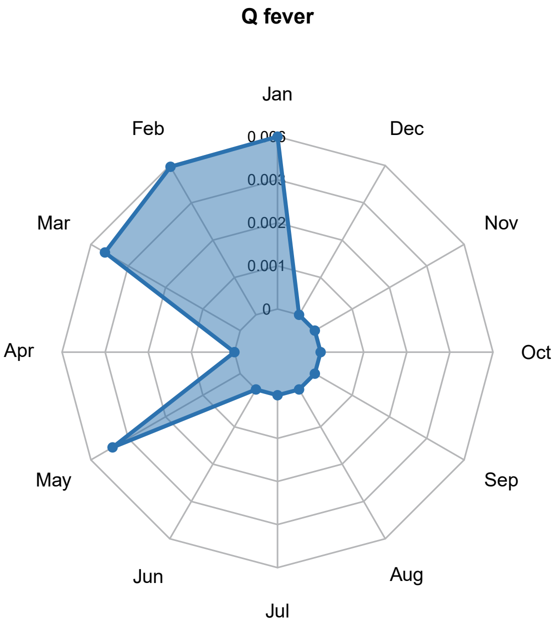
Q fever
Jan
Feb
Dec
Mar
Nov
Apr
Oct
May
Sep
Jun
Aug
Jul
0.002
0.001

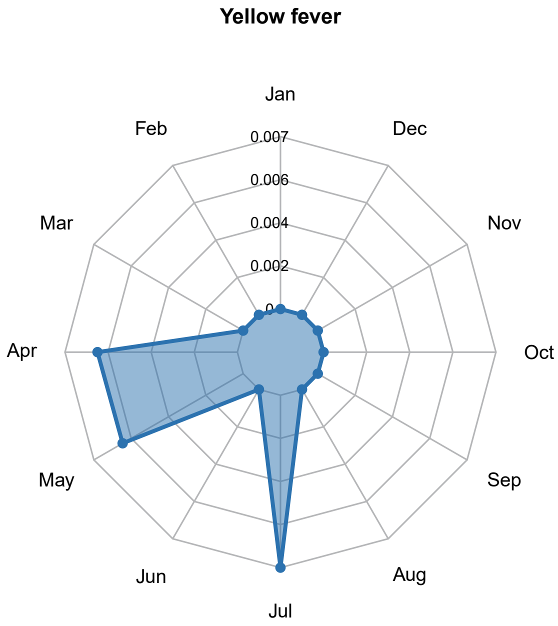
Yellow fever
Jan
Feb
Dec
Mar
Nov
Apr
Oct
May
Sep
Jun
Aug
Jul
0.007
0.006
0.004
0.002

**Eastern equine encephalitis**

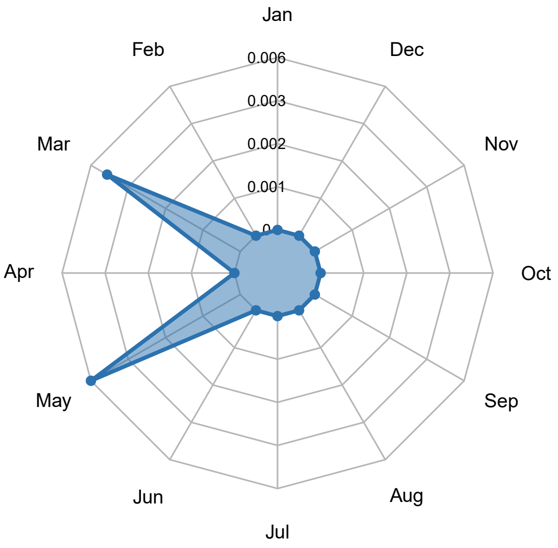


**Melioidosis**

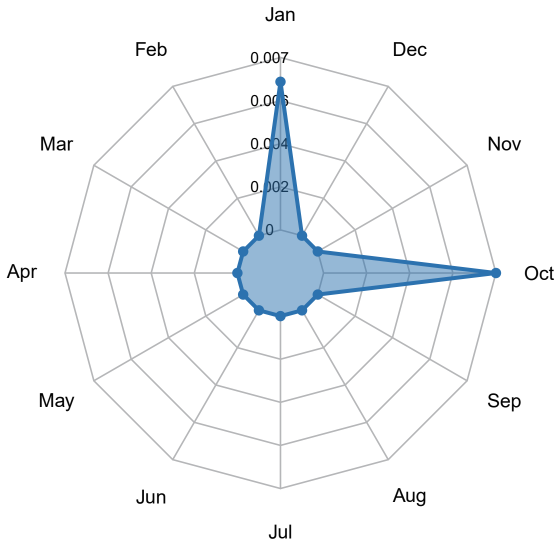


**African trypanosomiasis**

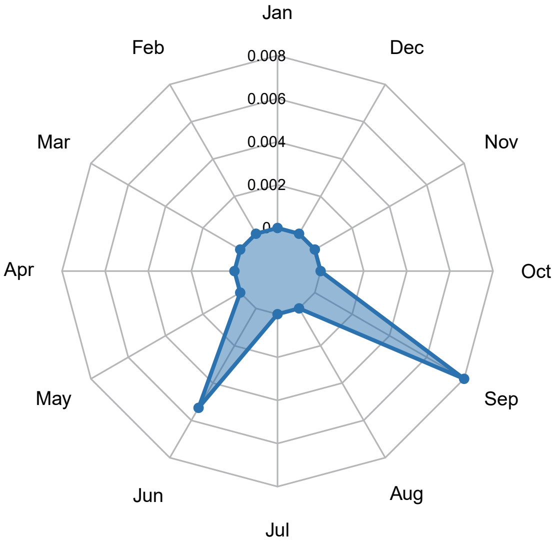


**Visceral leishmaniasis**

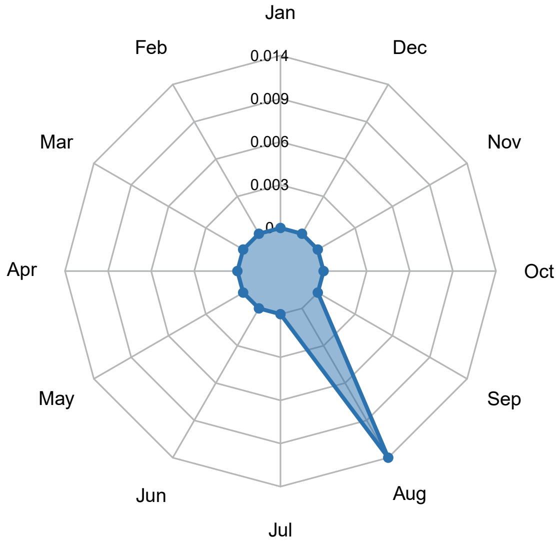


**Dengue**

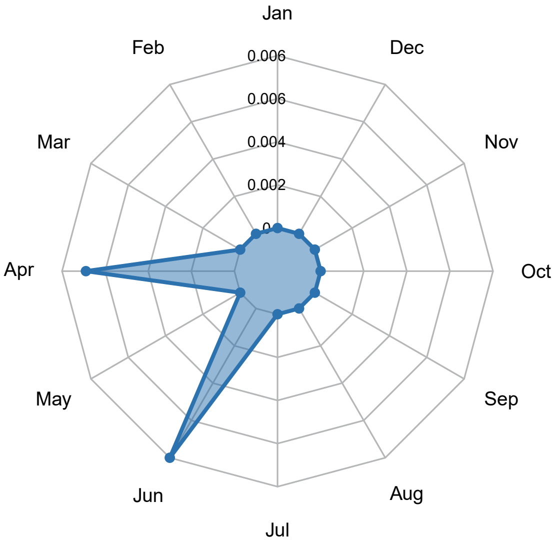


**Bartonellosis**

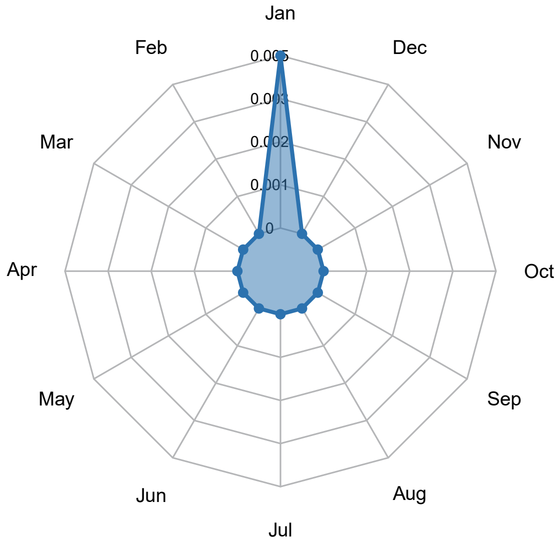

**Crimean−Congo haemorrhagic fever**

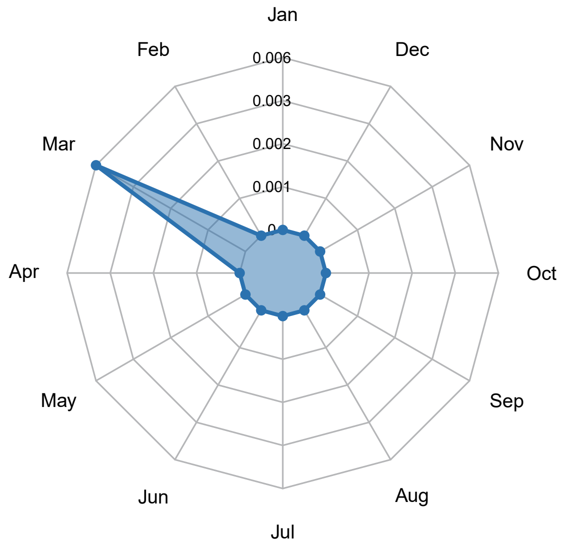


**Rickettsialpox**

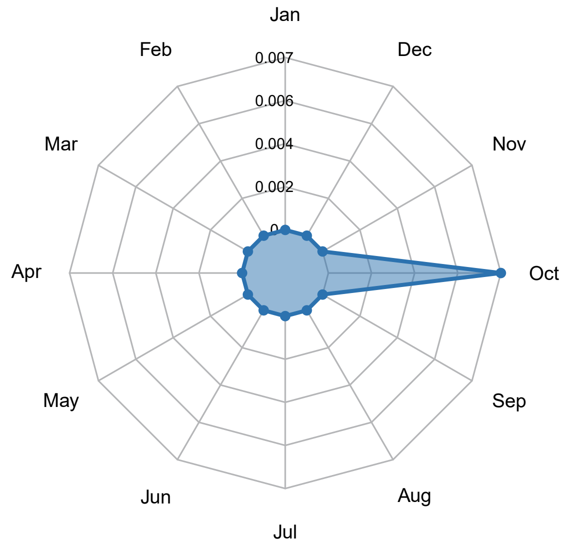


**Lyme borreliosis**

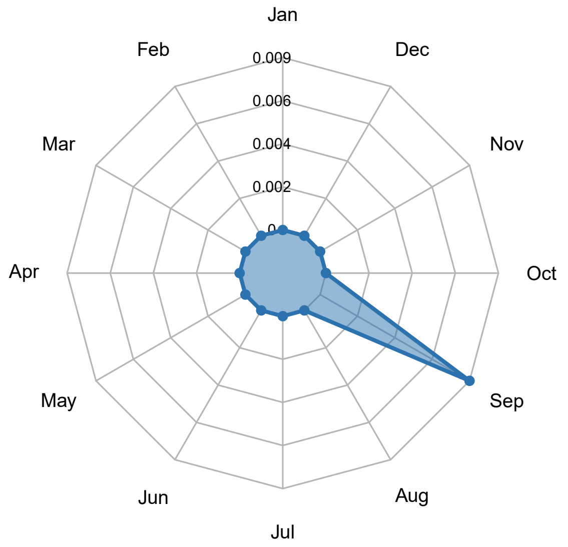


**Hepatitis B**

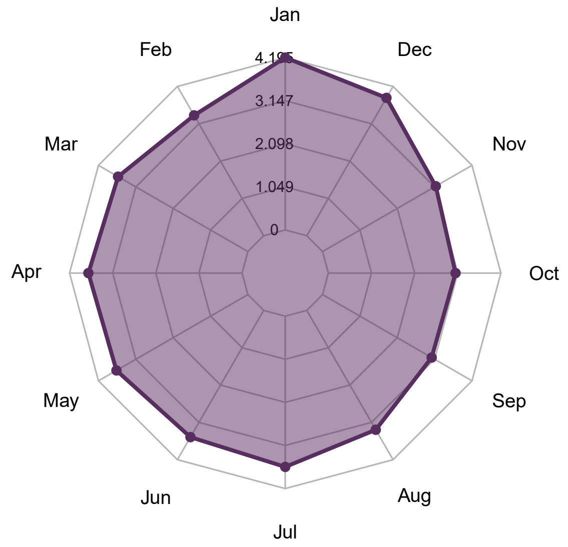


**Syphilis**

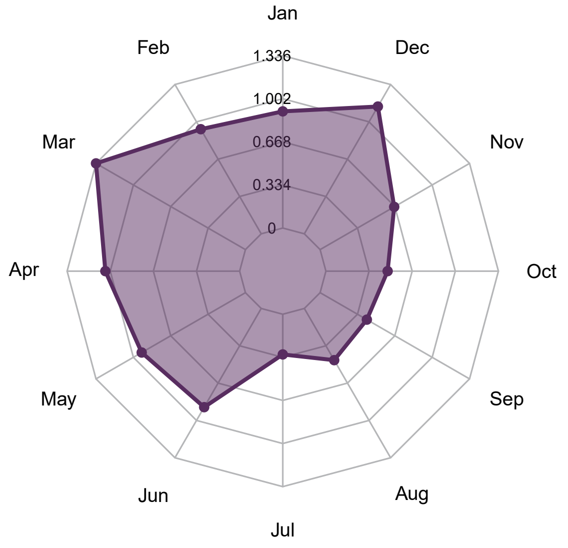


**Hepatitis C**

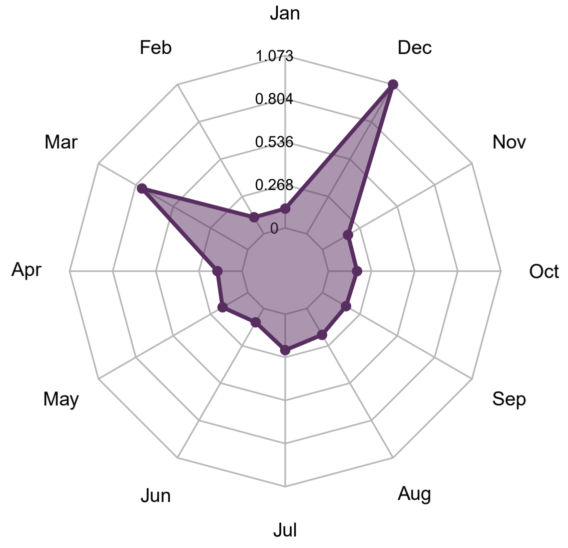

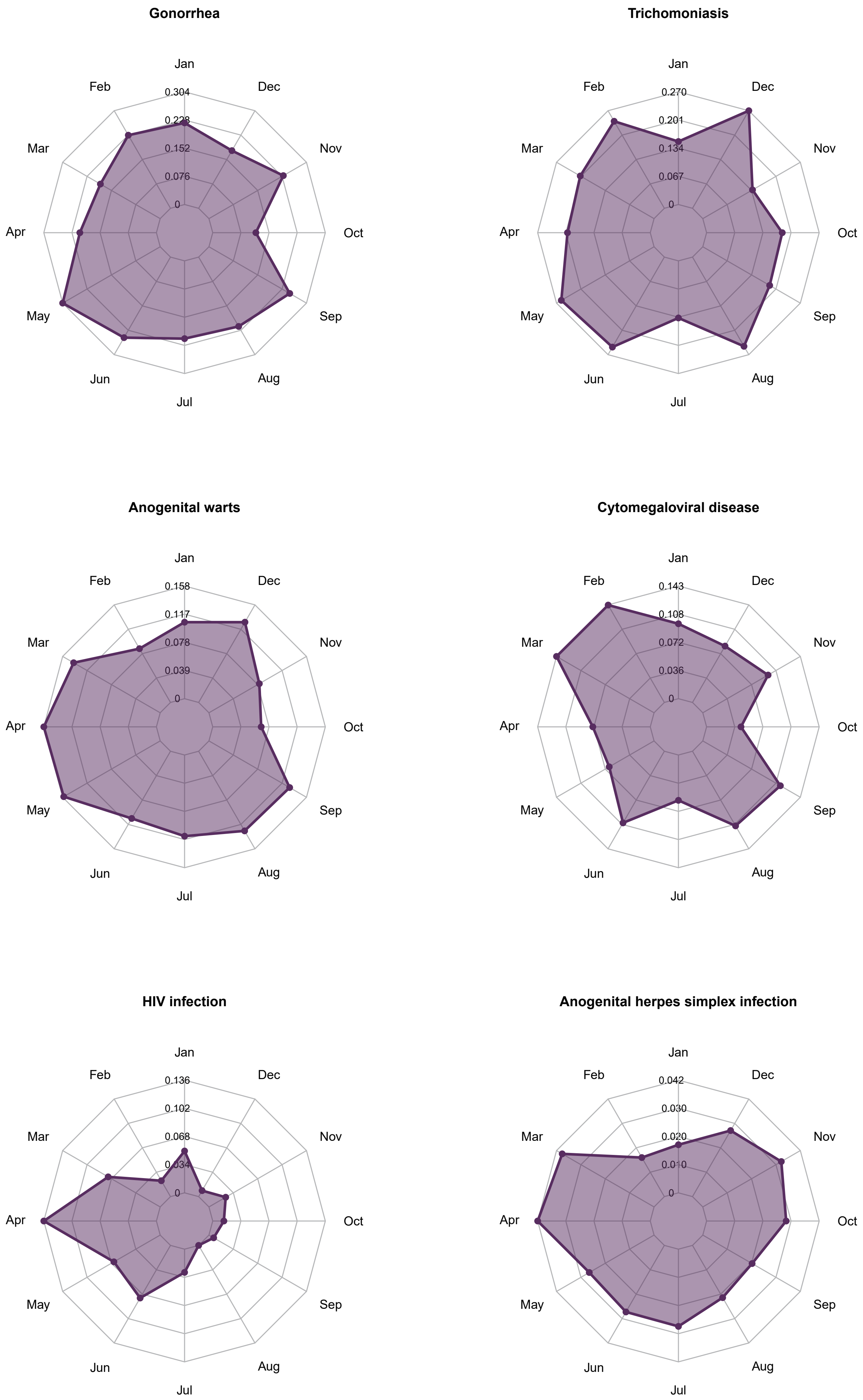
Gonorrhea
Jan
Feb
Mar
Apr
May
Jun
Jul
Aug
Sep
Oct
Nov
Dec
0.304
0.228
0.152
0.076
0
Trichomoniasis
0.270
0.201
0.134
0.067
0
Anogenital warts
0.158
0.117
0.078
0.039
0
Cytomegaloviral disease
0.143
0.108
0.072
0.036
0
HIV infection
0.136
0.102
0.068
0.034
0
Anogenital herpes simplex infection
0.042
0.030
0.020
0.010
0

**Granuloma inguinale**

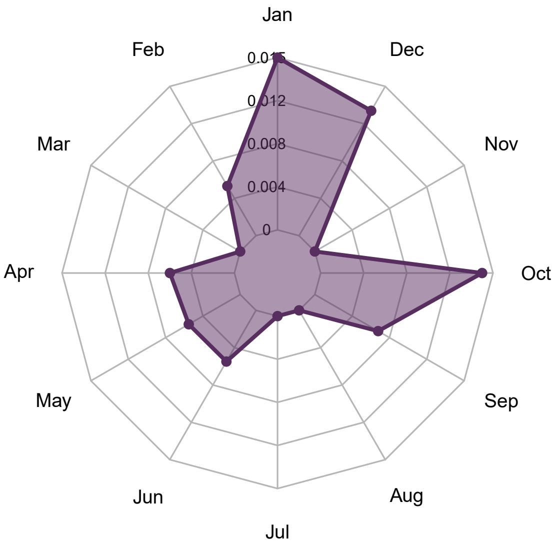


**Hepatitis D**

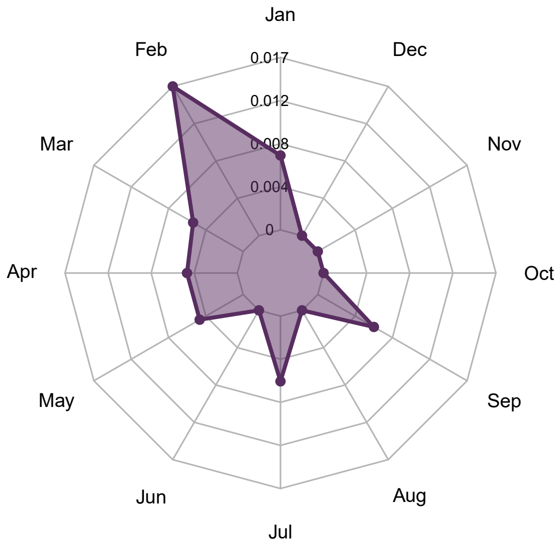


**Chancroid**

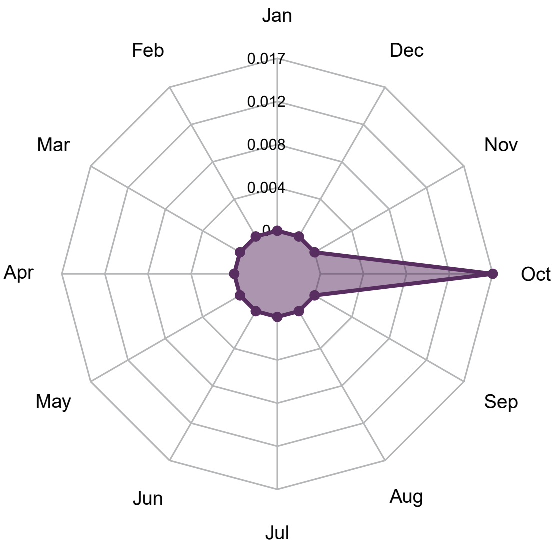


**Warts of lips or oral cavity**

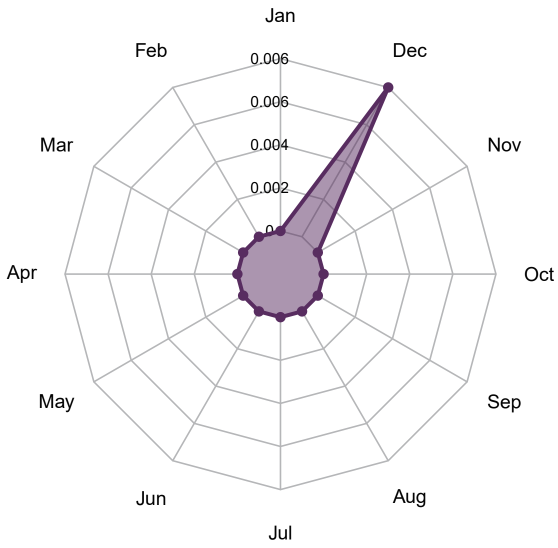


**Zoster**

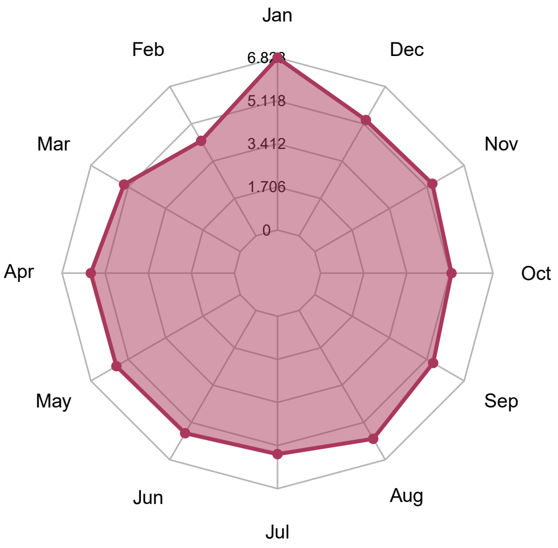


**Hand, foot, and mouth disease**

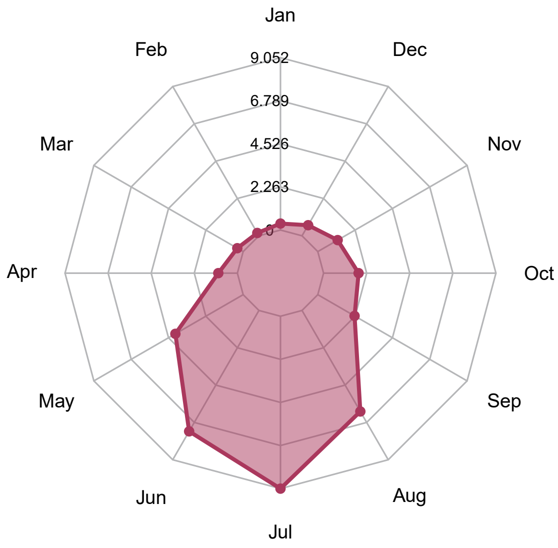

**Viral conjunctivitis**

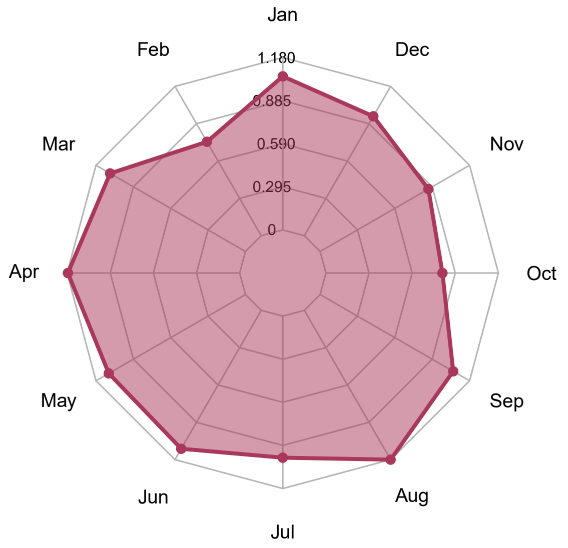


**Trachoma**

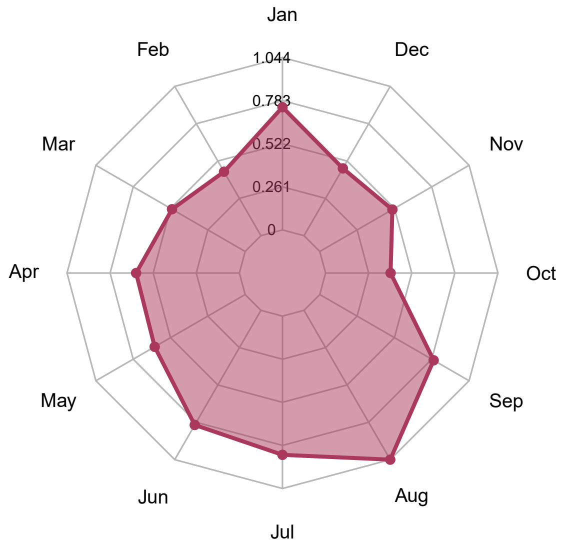


**Common warts**

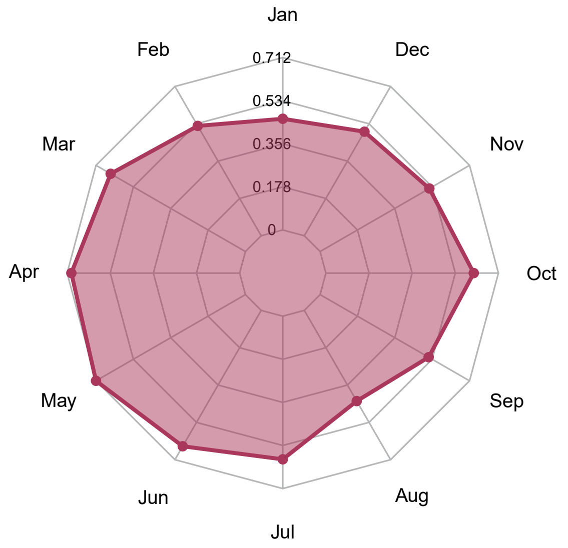


**Herpes simplex infections**

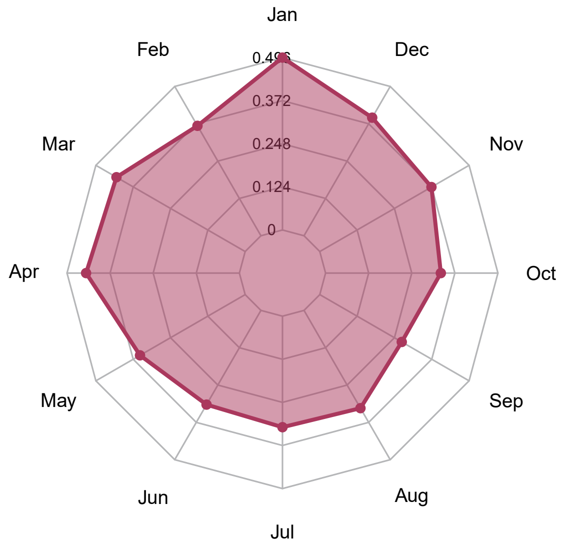


**Plane warts**

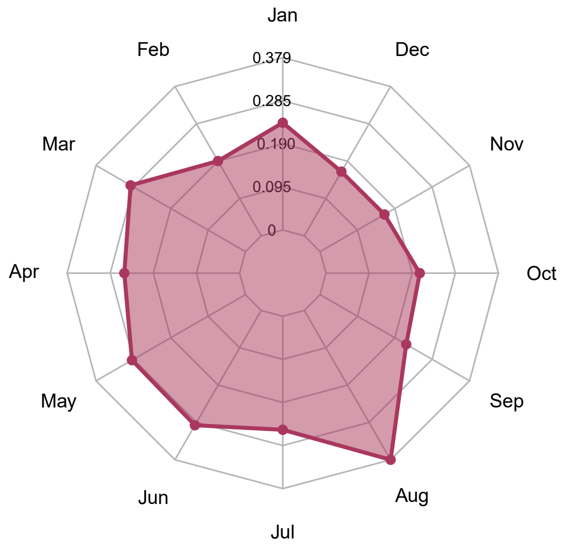


**Impetigo**

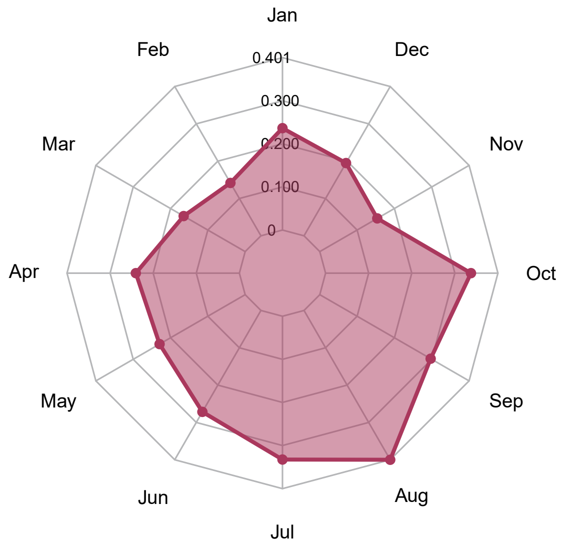

**Non−dermatophyte superficial dermatomycoses**

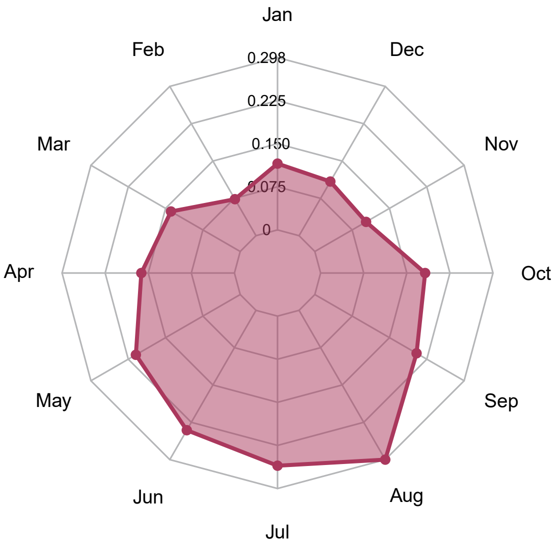


**Molluscum contagiosum**

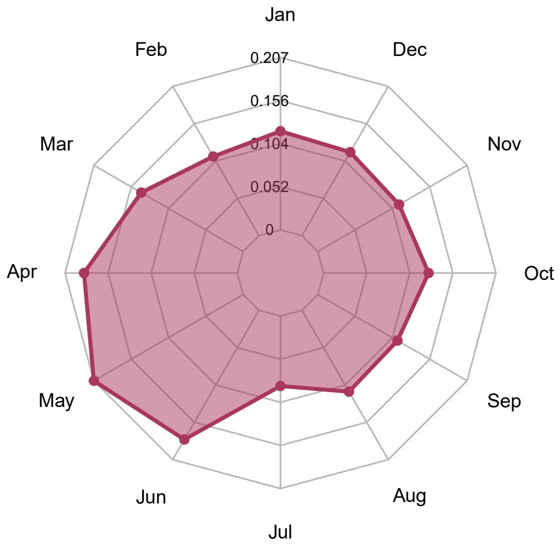


**Infectious mononucleosis**

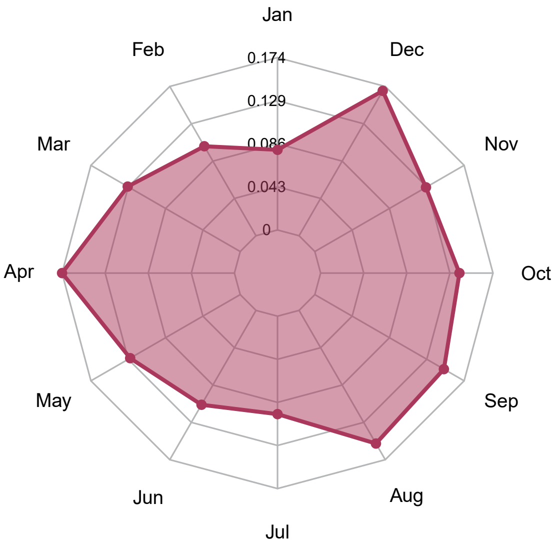


**Scabies**

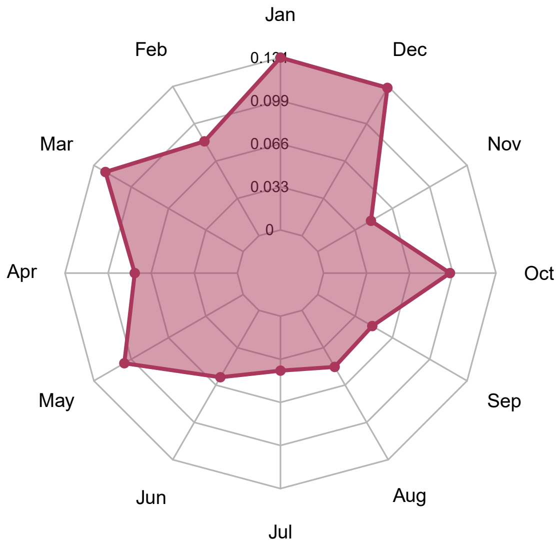


**Picornavirus infections presenting in the skin or mucous membranes**

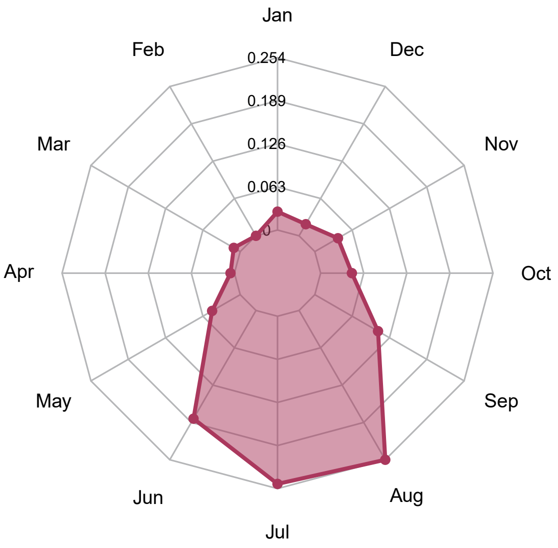


**Myiasis**

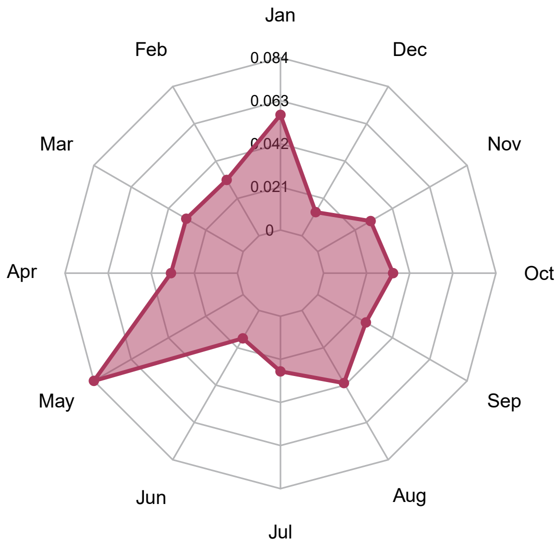

**Tetanus**

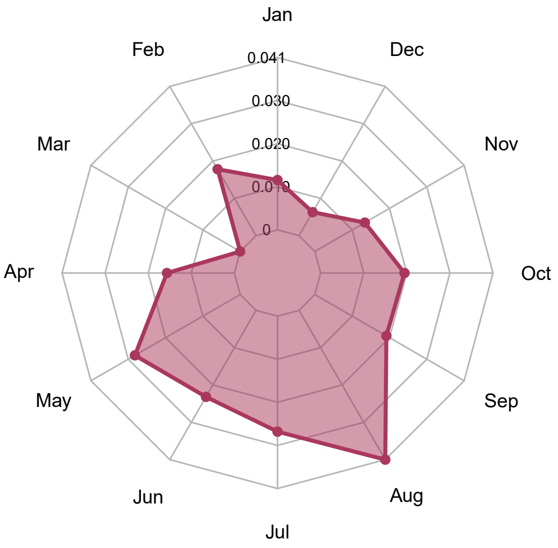


**Rabies**

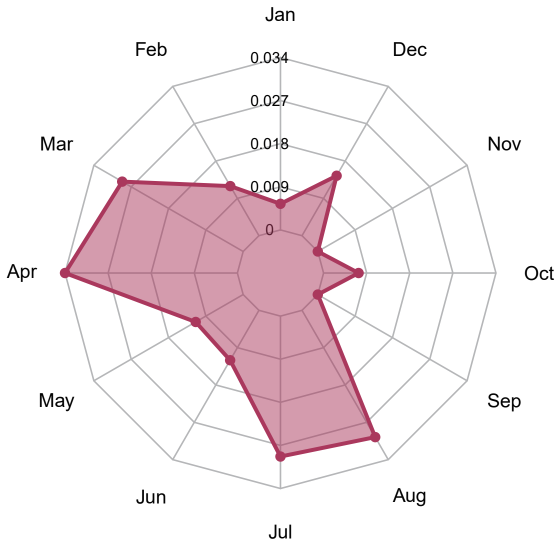


**Yaws**

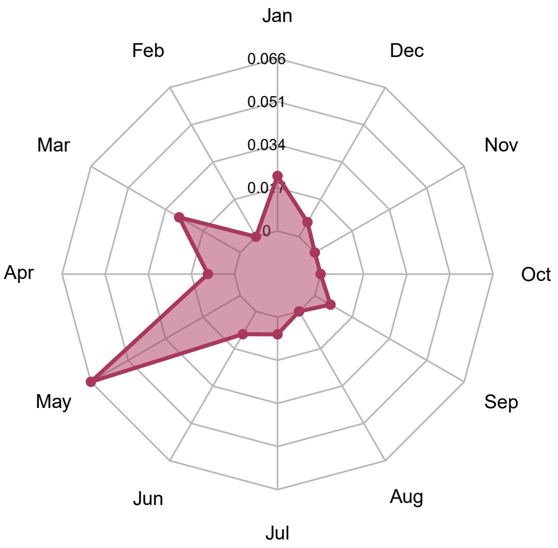


**Acute hemorrhagic conjunctivitis**

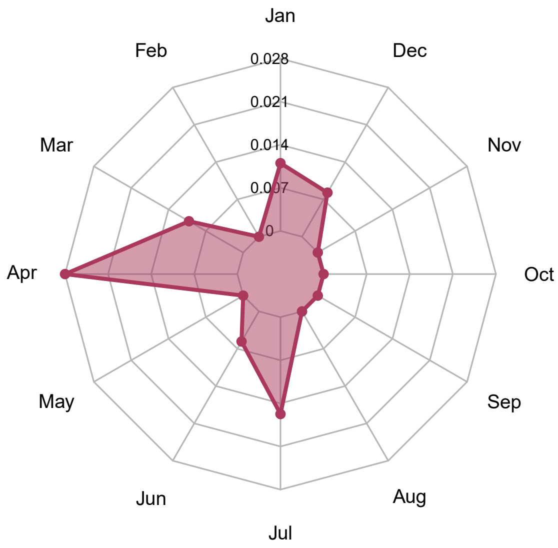


**Eumycetoma**

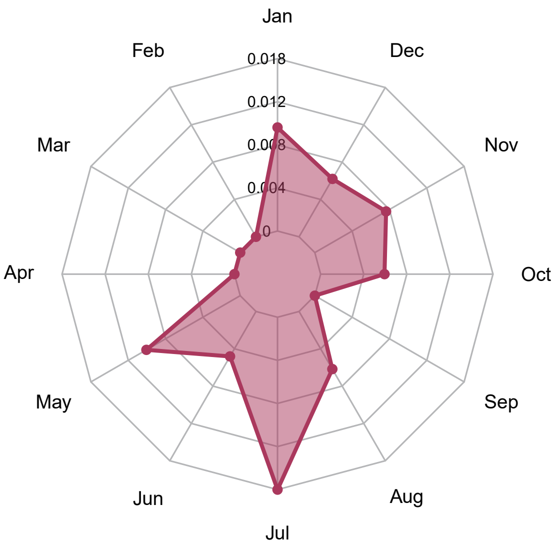


**Infestation by mites**

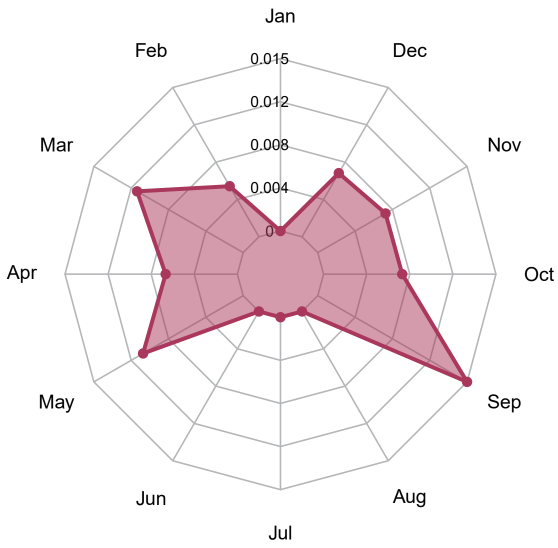

**Infantile papular acrodermatitis**

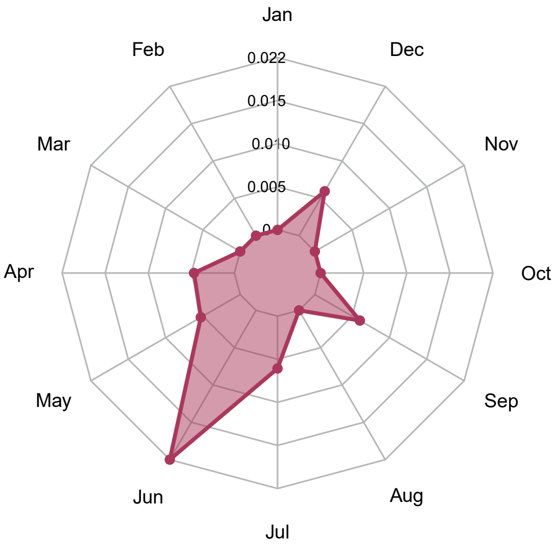


**Echinococcosis**

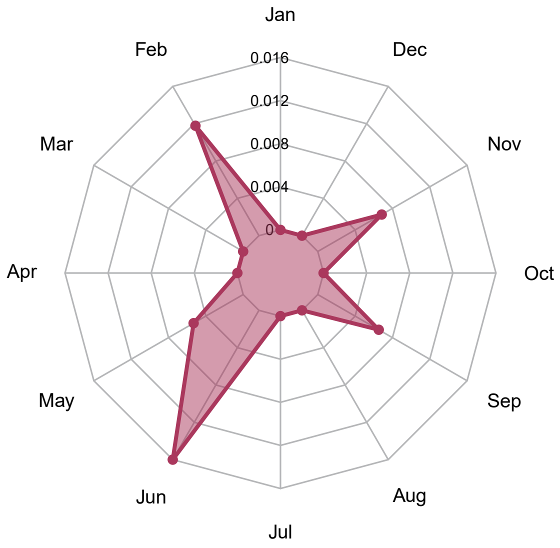


**Sporotrichosis**

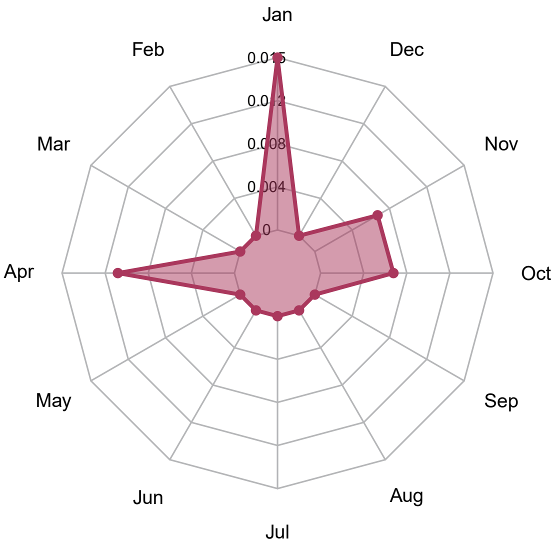


**Strongyloidiasis**

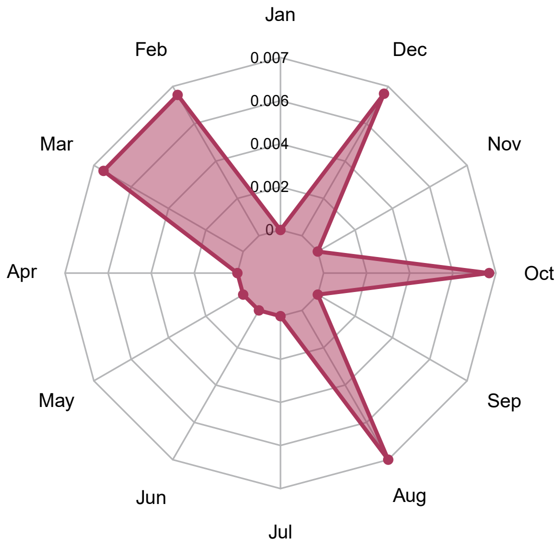


**Obstetrical tetanus**

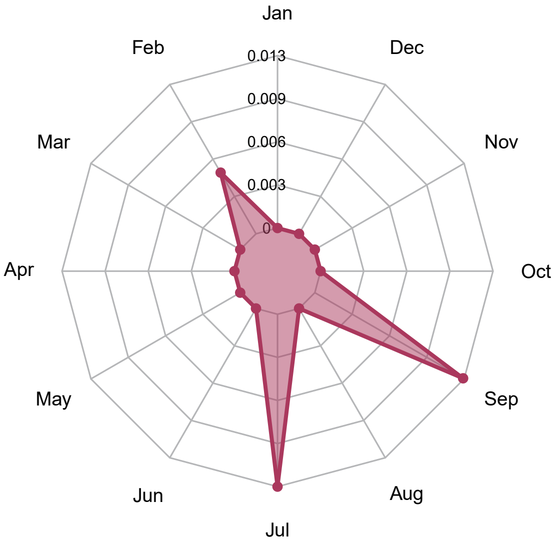


**Hookworm diseases**

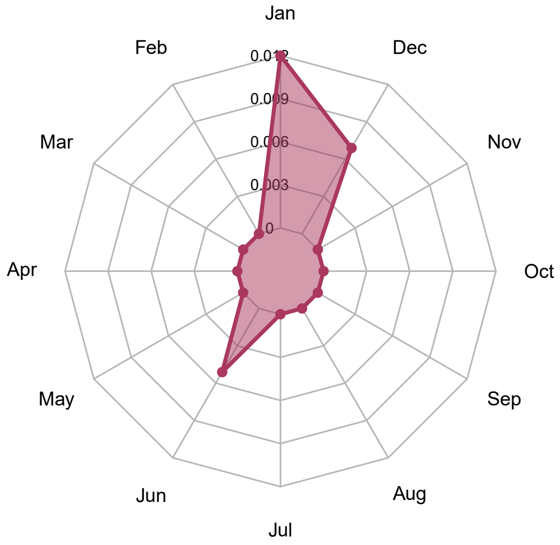

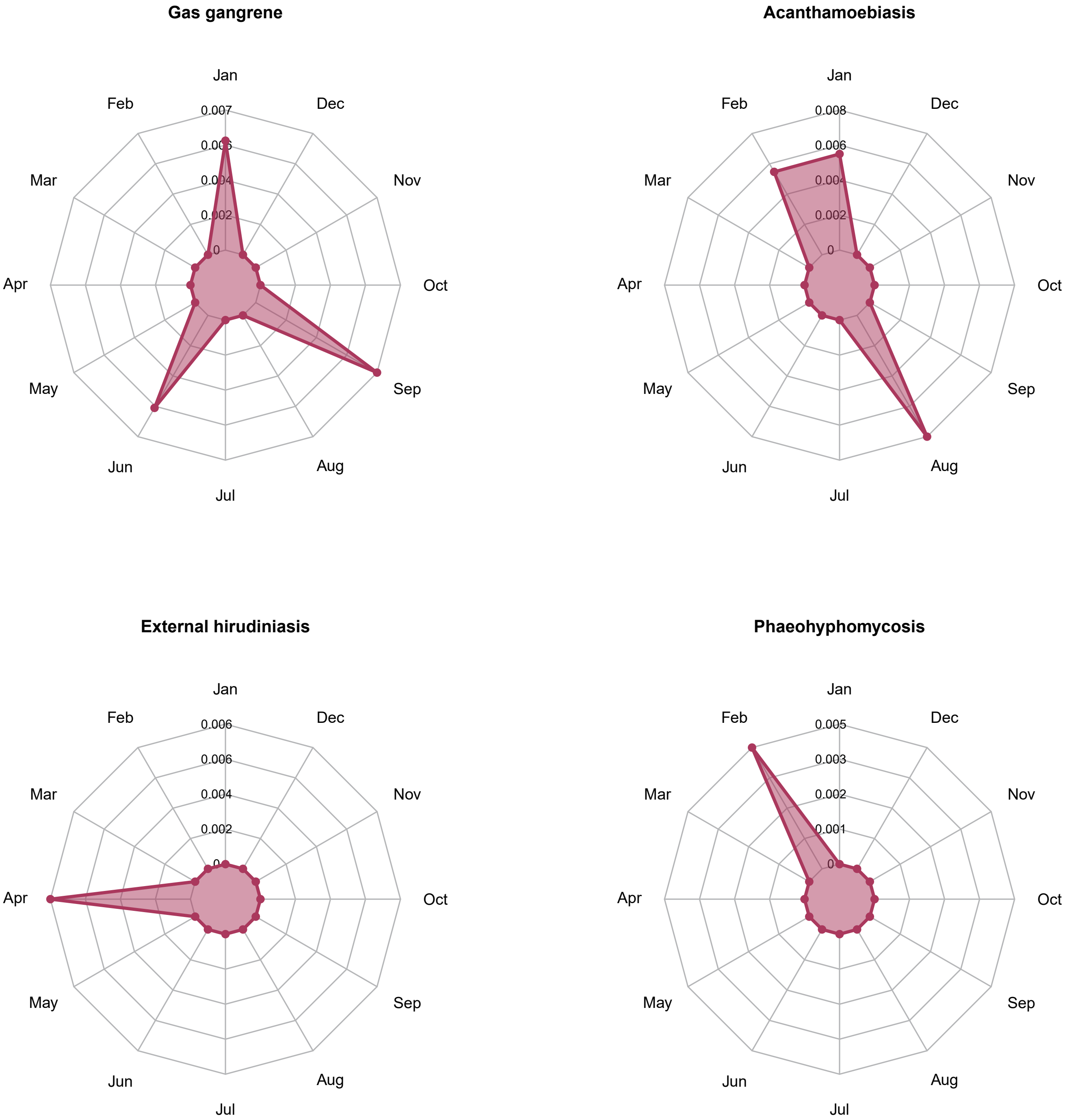


**Figure S4: Seasonal distribution of 130 infectious diseases in Shandong province, China, 2013-2017**
**HIV=human immunodeficiency virus, PIPSM=picornavirus infections presenting in the skin or mucous membranes.**

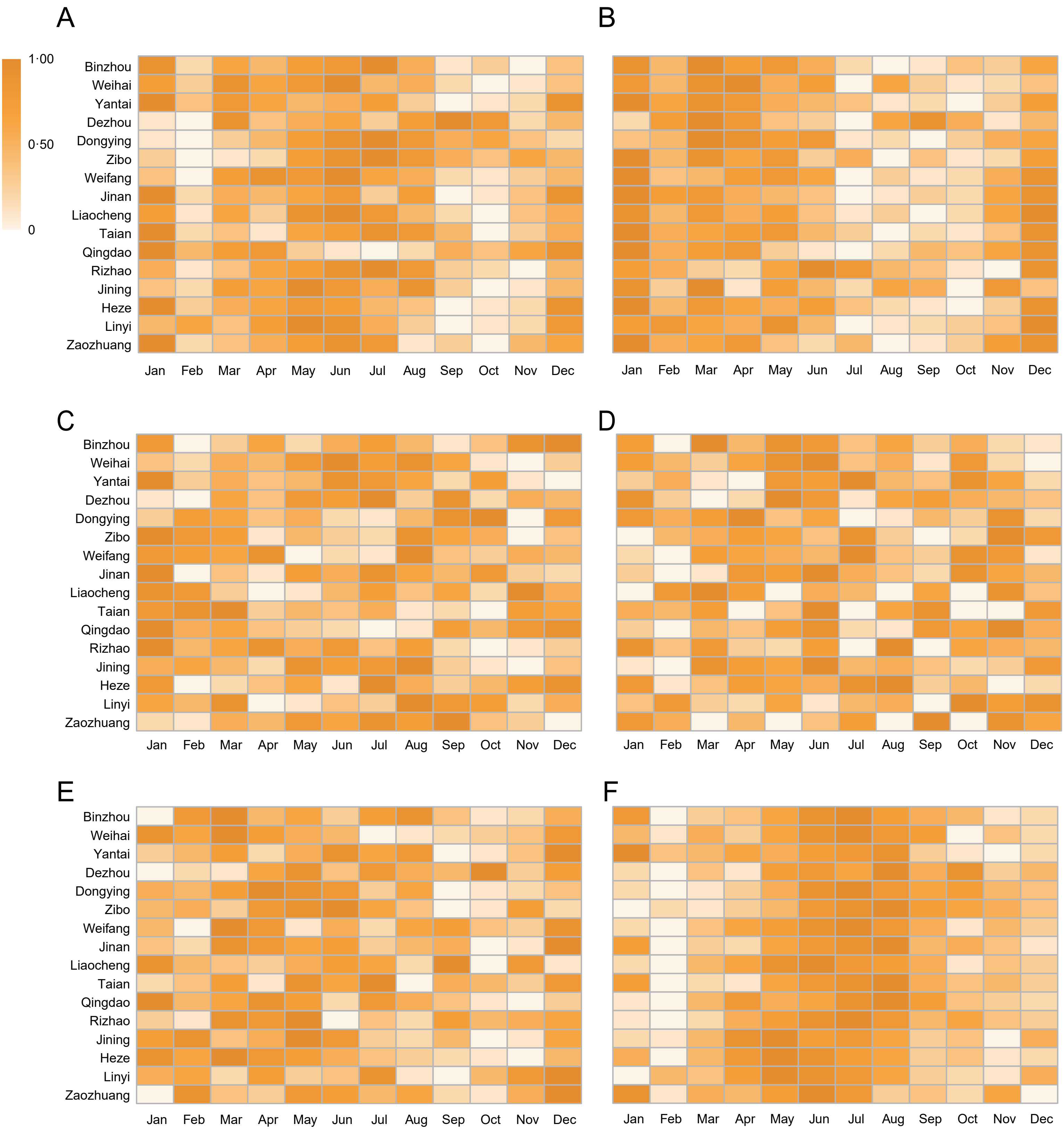


**Figure S5: Seasonal distribution of the five categories of infectious diseases in 16 prefecture-level cities of Shandong province, China**
**(A) Total infections. (B) Respiratory infections. (C) Gastrointestinal infections. (D) Vector-borne infections. (E) Blood- and sexually transmitted infections. (F) Mucocutaneous infections.**

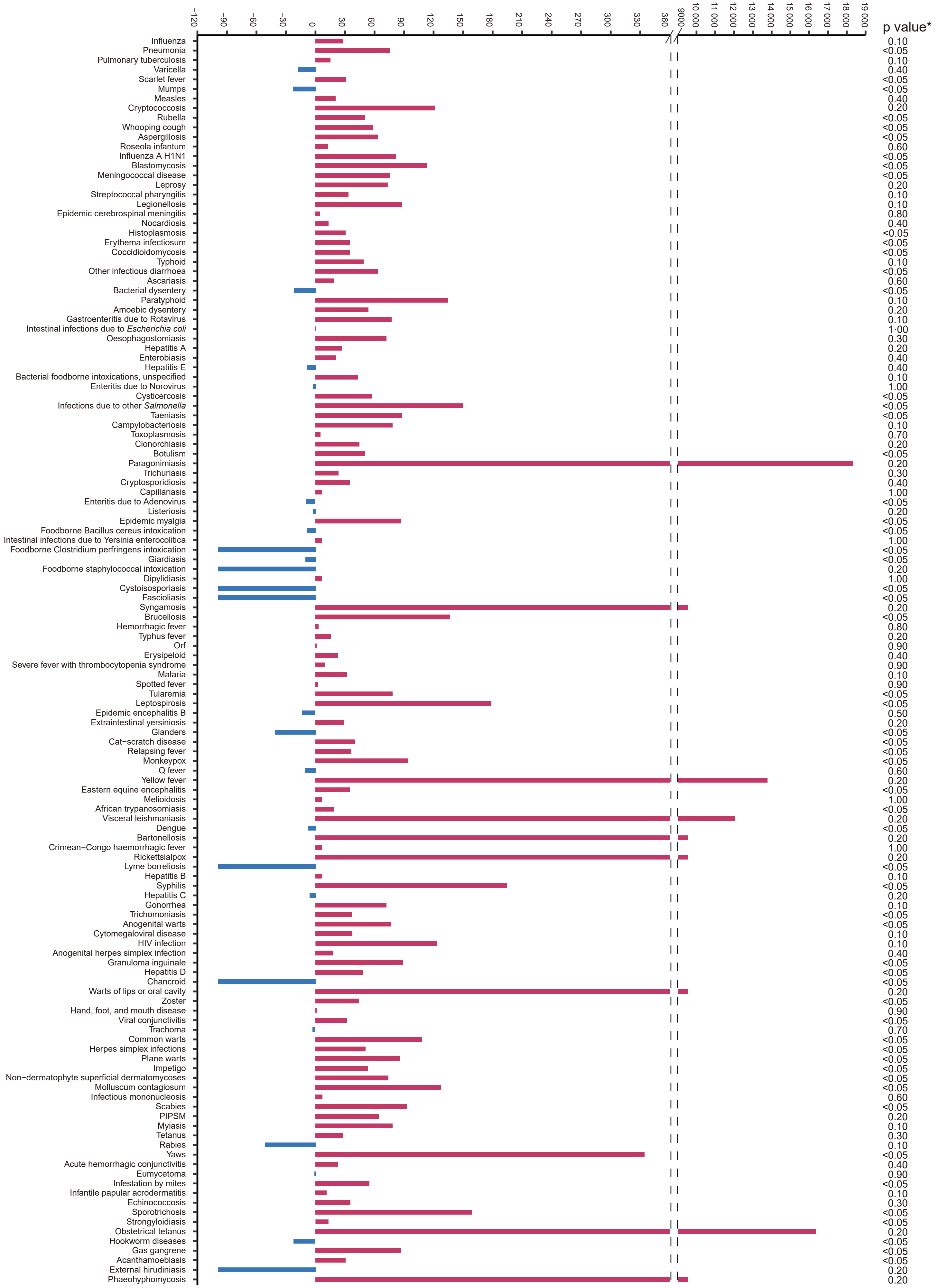


**Figure S6: Annual percentage change in the incidence density of 130 infectious diseases.**
HIV=human immunodeficiency virus, PIPSM=Picornavirus infections presenting in the skin or mucous membranes.

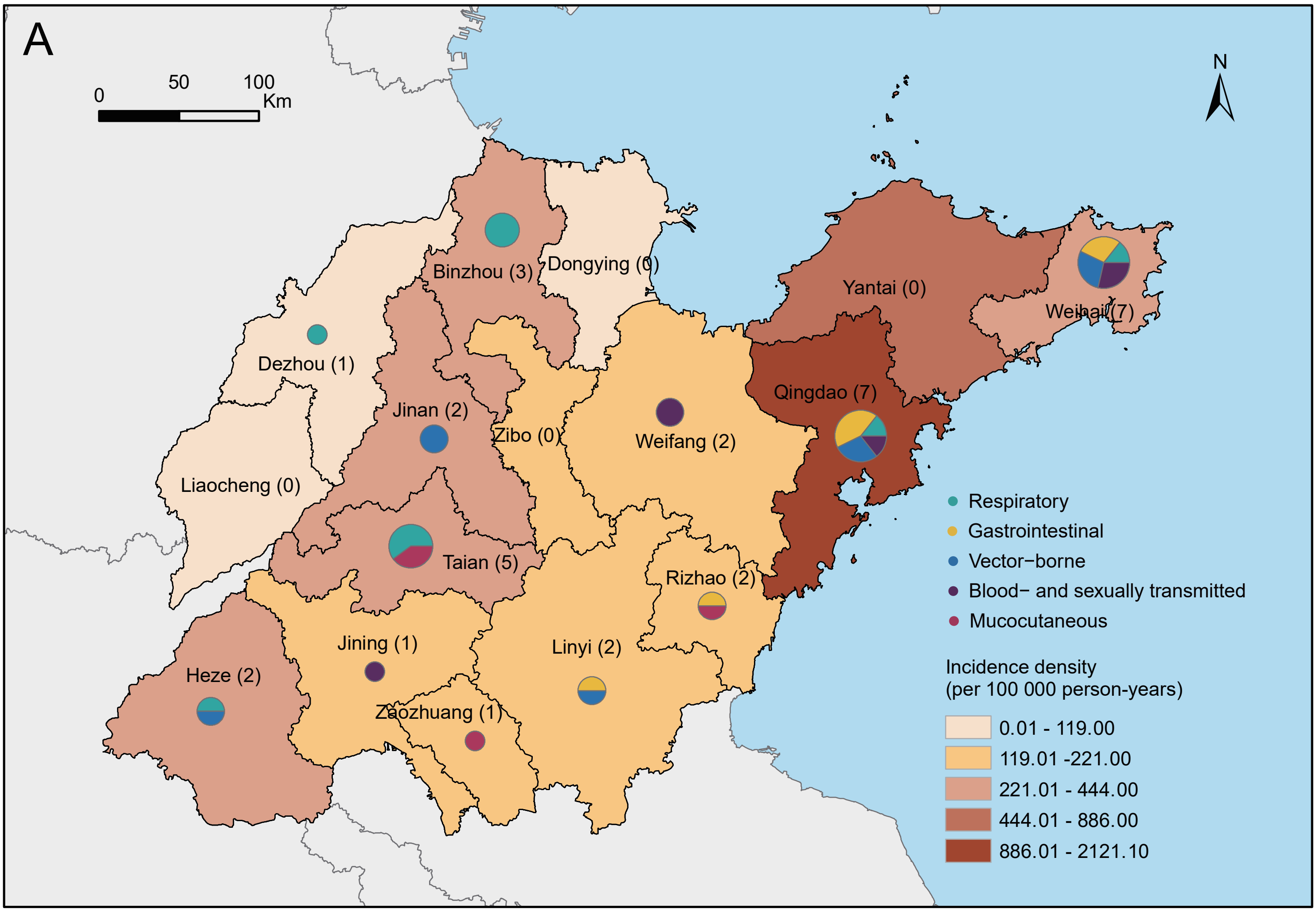


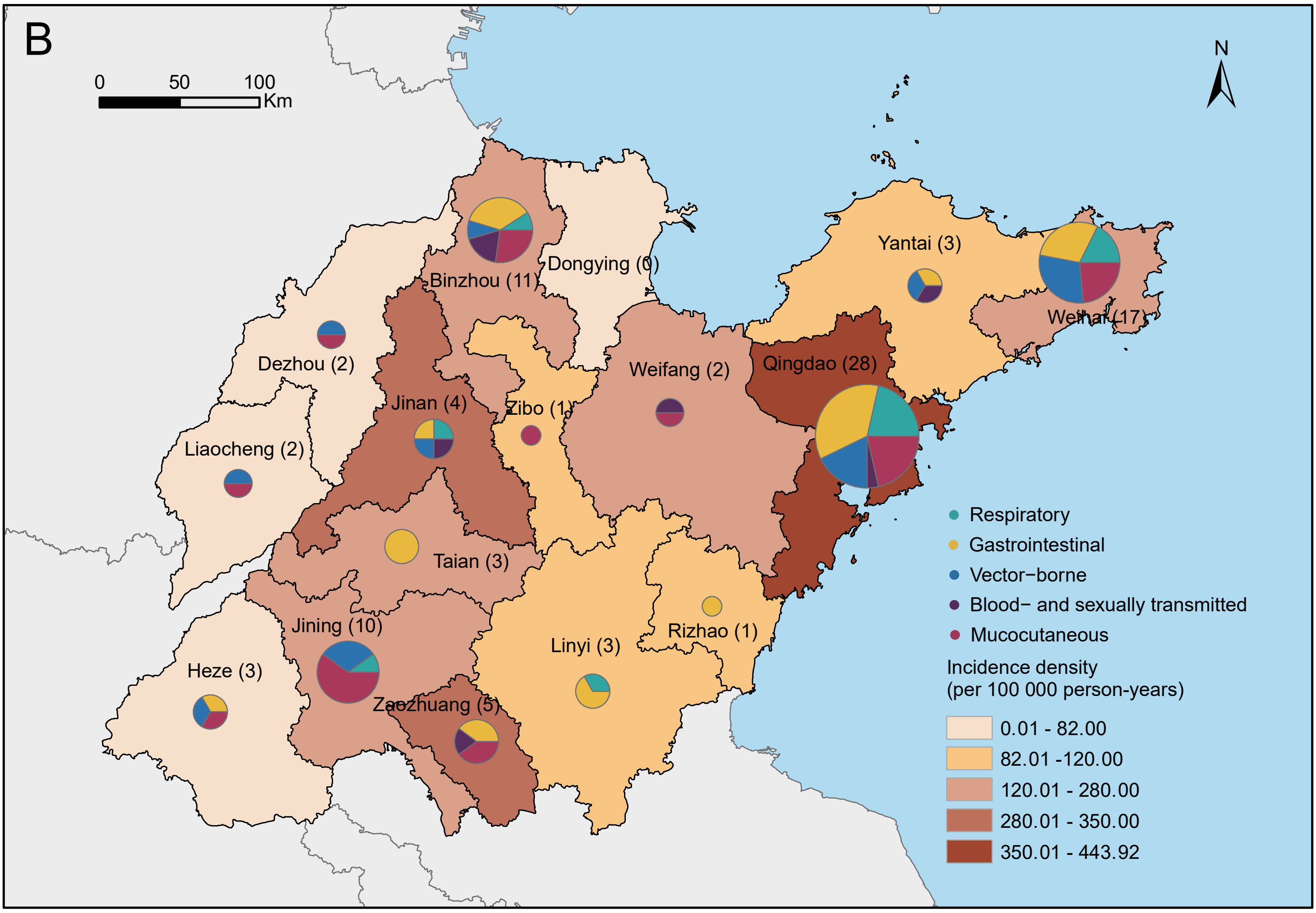


**Figure S7: Geographical distribution of 35 notifiable infectious diseases and 95 non-notifiable infectious diseases in Shandong province, China**

(A) Notifiable infectious disease, and (B) non-notifiable infectious disease. The circle represents cities where the infectious diseases with the highest incidence density distributed, with the circle size and numbers in parentheses indicating the disease number.
